# Pax6 Regulates Gene Expression in the Vertebrate Lens through miR-204

**DOI:** 10.1371/journal.pgen.1003357

**Published:** 2013-03-14

**Authors:** Ohad Shaham, Karen Gueta, Eyal Mor, Pazit Oren-Giladi, Dina Grinberg, Qing Xie, Ales Cvekl, Noam Shomron, Noa Davis, Maya Keydar-Prizant, Shaul Raviv, Metsada Pasmanik-Chor, Rachel E. Bell, Carmit Levy, Raffaella Avellino, Sandro Banfi, Ivan Conte, Ruth Ashery-Padan

**Affiliations:** 1 Department of Human Molecular Genetics and Biochemistry, Sackler Faculty of Medicine, Tel Aviv University, Tel Aviv, Israel; 2 Department of Cell and Developmental Biology, Sackler School of Medicine, Tel Aviv University, Tel Aviv, Israel; 3 Department of Genetics and Department of Ophthalmology and Visual Sciences, Albert Einstein College of Medicine, Bronx, New York, United States of America; 4 Sagol School of Neuroscience, Tel Aviv University, Tel Aviv, Israel; 5 Bioinformatics Unit, Faculty of Life Sciences, Tel Aviv University, Tel Aviv, Israel; 6 Telethon Institute of Genetics and Medicine, Naples, Italy; 7 Medical Genetics, Department of Biochemistry, Biophysics and General Pathology, Second University of Naples, Naples, Italy; New York University, United States of America

## Abstract

During development, tissue-specific transcription factors regulate both protein-coding and non-coding genes to control differentiation. Recent studies have established a dual role for the transcription factor Pax6 as both an activator and repressor of gene expression in the eye, central nervous system, and pancreas. However, the molecular mechanism underlying the inhibitory activity of Pax6 is not fully understood. Here, we reveal that Trpm3 and the intronic microRNA gene *miR-204* are co-regulated by Pax6 during eye development. *miR-204* is probably the best known microRNA to function as a negative modulator of gene expression during eye development in vertebrates. Analysis of genes altered in mouse Pax6 mutants during lens development revealed significant over-representation of *miR-204* targets among the genes up-regulated in the Pax6 mutant lens. A number of new targets of *miR-204* were revealed, among them *Sox11*, a member of the SoxC family of pro-neuronal transcription factors, and an important regulator of eye development. Expression of *Trpm/miR-204* and a few of its targets are also Pax6-dependent in medaka fish eyes. Collectively, this study identifies a novel evolutionarily conserved mechanism by which Pax6 controls the down-regulation of multiple genes through direct up-regulation of *miR-204*.

## Author Summary

The transcription factor Pax6 is reiteratively employed in space and time for the establishment of progenitor pools and the differentiation of neuronal and non-neuronal lineages of the CNS, pancreas, and eye. Execution of these diverse developmental programs depends on simultaneous activation and repression of gene networks functioning downstream of Pax6. MicroRNAs function as inhibitors of gene expression. Many microRNA genes are transcribed through common promoters of host genes. In this study, using wide-scale analysis of changes in gene expression following Pax6 deletion in the lens, we discover that Pax6 regulates the gene *Trpm3* and its hosted microRNA, miR-204. We then show that miR-204 suppresses several target genes in the lens, notably the neuronal gene *Sox11*. Lastly, by conducting parallel experiments in the medaka fish, we show that Pax6 control of miR-204 and its target genes is evolutionarily conserved between mammals and fish, stressing the biological importance of this pathway. Pax6 regulation of *miR-204* explains part of the complex, divergent inhibitory activity of Pax6 in ocular progenitor cells, which is required to establish and maintain the identity and function of ocular tissues.

## Introduction

Lineage-specific transcription factors (TFs) such as *Pax6* direct the development of multiple tissues through the regulation of gene networks that execute discrete developmental programs. *Pax6* is essential for normal development of the central nervous system (CNS), pancreas, olfactory system and eye (reviewed in [1], [2]). *Pax6* is considered a “master regulator” of eye development as it specifies the multiple cell lineages that comprise the eye in vertebrate and invertebrate species [3].

During embryonic development, Pax6 protein is known to activate several target genes using two DNA-binding domains and a proline-serine-threonine transcription activating domain [4]–[6]. Pax6 may also enhance gene expression by recruiting chromatin-remodeling enzymes and alleviating heterochromatin repression [4], [7], [8]. In contrast, Pax6 has been found to function as a repressor of the lens crystallin genes *Cryabb1* and *Crygf,* and of the photoreceptor TF *Crx* [4], [9]–[11]. Since repression of crystallin genes was shown to be independent of the transactivation domain of Pax6, it was proposed that inhibition is mediated by competition for promoter occupancy with other TFs [4], [9], [10]. However, additional mechanisms of Pax6-dependent gene repression remain to be identified.

MicroRNAs (miRNAs) direct post-transcriptional repression of a wide array of genes by adhering to miRNA-specific sequences in the 3′ untranslated region (UTR) of mRNAs [12]. In this way, a single type of miRNA can essentially bind dozens of different mRNA transcripts [13]. Therefore, regulation of a miRNA gene by a tissue-specific TF can facilitate the down-regulation of batteries of genes.

To ascertain the roles of miRNAs in eye development, somatic mutations of the miRNA-maturation enzyme gene *Dicer1* were examined in the mouse lens and retinal progenitors cells (RPCs, [14]–[16]). When *Dicer1* was knocked out at the lens placode (LP) stage, lens development proceeded to primary lens fiber cell differentiation; however, secondary lens fiber cell differentiation was aborted and lens epithelium (LE) cells ceased to divide, undergoing apoptosis. Therefore, it is evident that miRNAs play an important role in the late stages of lens development. Somatic mutation of *Dicer1* in RPCs revealed multiple activities of miRNAs in their specification, differentiation and survival [15], [16].

**Figure 1:**
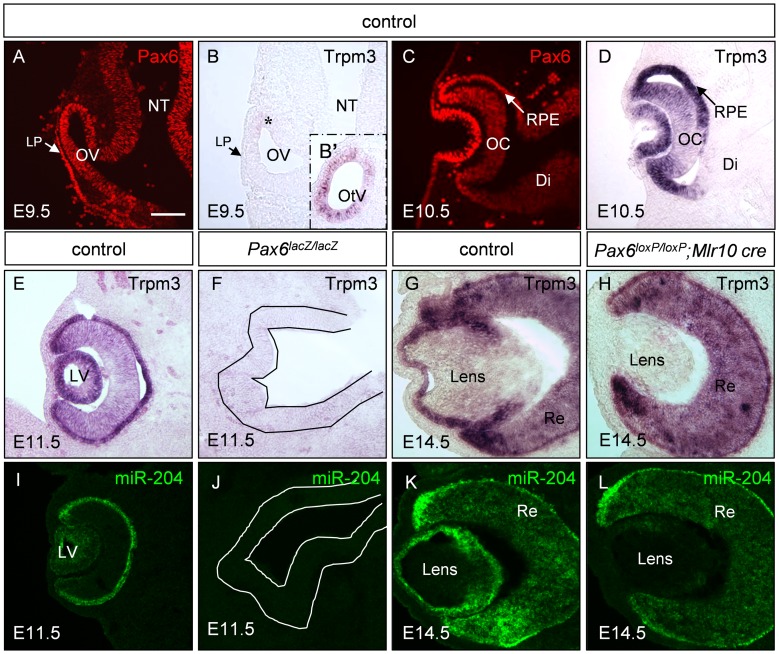
*Trpm3/miR-204* expression is dependent on Pax6 activity during eye development. Paraffin sections of control (A–E,G,I,K), *Pax6^lacZ/lacZ^* (F,J) and *Pax6^loxP/loxP^;Mlr10-cre* (H,L) stained for *Trpm3* mRNA (B,D,E–H), Pax6 protein (A,C, red) and *miR-204* (I–L, green). *Trpm3* expression begins after E9.5 in the developing eye (B,D), although it is already active in the otic vesicle on E9.5 (B′ inset). *Trpm3* and *miR-204* are lost from the optic rudiment of *Pax6^lacZ/lacZ^* embryos (F,J; optic cup rudiment is traced with a line) and in the lens of *Pax6^loxP/loxP^;Mlr10-cre* mutants (H,L). Di, diencephalon; LP, lens placode; LV, lens vesicle; OV, optic vesicle; NT, neural tube; OC, optic cup; OtV, otic vesicle; RPE, retinal pigmented epithelium; Re, developing retina. Scale bar = 100 µM.

To date, there is limited information on the function of specific miRNAs in the eye. Probably the most extensively studied example is *miR-204*. In medaka fish (*Oryzias latipes; ol*), *ol-miR-204* was shown to affect lenticular and retinal development via repression of *Meis2* and its transcriptional target *Pax6* [17]. In addition, *miR-204* was found to contribute to the epithelial physiology of human retinal pigmented epithelium (RPE) [18], [19]. However, the activity and regulation of *miR-204* in the mammalian lens and retina remain unknown. The coding region for the mouse *miR-204* resides in intron 6 of the transient receptor potential cation channel M3 gene (*Trpm3* [20]). *miR-204* appears to be concomitantly expressed with *Trpm3* in the eye and CNS [18], [21], [22]. In the post-natal mouse eye, its pattern resembles that of Pax6 (Figure 1;[21], [23]).

The present study was aimed at elucidating the molecular mechanism of Pax6-dependent transcriptional repression through unbiased analysis of up-regulated genes in *Pax6-*mutant lenses. We show that inhibition of gene expression by *Pax6* is at least partly mediated through direct activation of *miR-204*. In addition, we identify *Sox11* as a novel target for *miR-204* in lens and retinal development. Finally, both regulation of *Trpm/miR-204* by Pax6 and inhibition of *Sox11* are shown to be conserved in vertebrates. This study is the first to reveal that miRNAs are part of the Pax6 genetic network in different vertebrate species, adding to the known repertoire of Pax6 activities in the course of organ development.

## Results

### Large-scale changes in the lens transcriptome as a result of *Pax6* deletion

To identify new *Pax6* genetic targets in the developing lens, an expression microarray was performed on embryonic day 14.5 (E14.5) lenses from controls and somatic mutants of *Pax6* (*Pax6^loxP/loxP^*;*Mlr10-cre*, [24]). Out of 28,853 mouse genes, 315 were altered at a cutoff of 2.0-fold and a *P*-value below 0.05. Of these, the expression levels of 235 genes were increased upon *Pax6* deletion, while only 83 genes were reduced (*P*-values and fold change are shown Figure S1 and the genes are listed in Table S1). A bias toward up-regulated genes - 2.8∶1 in the lens, has also been reported in the embryonic neocortex (1.8∶1, 600 up-regulated vs. 339 down-regulated genes; [25]). This raised the possibility of *Pax6*'s role in directly or indirectly repressing the expression of a large number of genes.

To identify common targets of *Pax6*, we compared the list of altered genes in the lens and embryonic neocortex [25]. Interestingly, only 22 genes were found to be regulated similarly in both systems, with 18 up-regulated and 4 down-regulated in both the lens and neocortex (Table 1). The four genes that were down-regulated in both lens and neuronal lineages are potential direct transcriptional targets of *Pax6*. One of these - *Trpm3* - contains both a coding region for Trpm3 and a non-coding miR-204 sequence. Trpm3 is a melastatin-like cation channel which is sensitive to steroids, active in insulin-producing beta cells and a chemo- and thermosensor in the somatosensory system [20], [26], [27], while miR-204 has been documented to play a role in ocular lineages in fish and mammals [17], [18] and thus may mediate Pax6's inhibitory activity in the eye.

**Table 1:** Pax6 targets that are reduced in developing lens (E14.5) and forebrain (E12.5).

Gene symbol	Fold-change	*P*-value
*Jph1*	−2.17472	0.002144
*Lix1*	−3.12141	0.013542
*Sema5a*	−2.32115	0.02874
***Trpm3***	−2.03299	0.002904

### Pax6 is required for *Trpm3/miR-204* expression during lens development

*Trpm3* transcript distribution in the post-natal eye shows some overlap with *Pax6* expression [18], [21], [22]. Considering the possibility that Pax6 regulates *Trpm3* expression directly during eye development, we compared the spatio-temporal expression patterns of Pax6 protein and *Trpm3* mRNA during eye morphogenesis and differentiation (Figure 1). Pax6 expression initiates in the neural tube on around E8.5 [28]. On E9.5, Pax6 is detected in the ocular progenitors of the optic vesicle (OV) and LP (Figure 1A). On E9.5, *Trpm3* is not yet detected in most ocular progenitors cells, and is barely observed in a small population of cells in the dorsal OV (Figure 1B, asterisk). Expression of *Trpm3* on E9.5 was, however, evident in the otic vesicle, the primordium of the inner ear (Figure 1B′ inset). In contrast, from E10.5 onwards, *Trpm3* mRNA was co-expressed with Pax6 protein in the lens and optic cup (OC) lineages. Both were strongly expressed in the invaginating lens vesicle and in the inner and outer layers of the OC (Figure 1C, 1D). *Trpm3* expression seemed to be restricted to the developing eye, inner ear and choroid plexus and did not extend to Pax6-positive cells of the developing diencephalon (Figure 1D, Figure S2A). The expression of *Trpm3* continued to follow that of *Pax6* during later stages of eye development and was detected in the inner nuclear layer and ciliary body after birth (Figure 1E, 1G; Figure S2B). Therefore, *Trpm3* expression in the developing eye began only after the establishment of ocular progenitor domains. From that stage onwards, *Trpm3* expression recapitulated the complex ocular pattern of *Pax6* expression, suggesting that *Pax6* regulates *Trpm3* expression in the developing lens and OC derivatives.

To determine whether Pax6 simultaneously regulates *Trpm3* and its hosted non-coding *miR-204*, Pax6 systemic knockout (*Pax6^lacZ/lacZ^*, E11.5) and Pax6 lens-specific conditional mutants (*Pax6^loxP/loxP^;Mlr10-cre*, E14.5) were examined (Figure 1). On E11.5, both *Trpm3* and *miR-204* were highly expressed in the lens vesicle and in the outer layer of the OC that contains the progenitors of the RPE, whereas lower levels were detected in the inner OC, where the RPCs reside (Figure 1E, 1I). Upon Pax6 ablation in *Pax6^lacZ/lacZ^* knock-out mice, *Trpm3/miR-204* expression on E11.5 was lost from the optic rudiment (Figure 1F, 1J). On E14.5, *Trpm3* and *miR-204* were both detected in the LE and RPE. In the inner layer of the OC, expression was more prominent in the distal regions containing the ciliary body and iris primordia (Figure 1G, 1K). When *Pax6* was removed specifically in the lens lineage on E14.5 (*Pax6^loxP/loxP^;Mlr10-cre*, [24]), both *Trpm3* and *miR-204* were lost from the mutant lens (Figure 1H, 1L). A reduction in *Trpm3* expression was also detected in *Pax6*-deficient OC (*Pax6^loxP/loxP^*;*a-cre* [29], see below) and in progenitors of the iris and ciliary body (*Pax6^loxP/loxP^;Dct-Cre* mutants, Figure S3). These findings strongly support genetic regulation of *Trpm3/miR-204* by Pax6 in several ocular tissue types.

### Pax6 directly binds *Trpm3* regulatory sequences *in vitro* and *in vivo*

**Figure 2:**
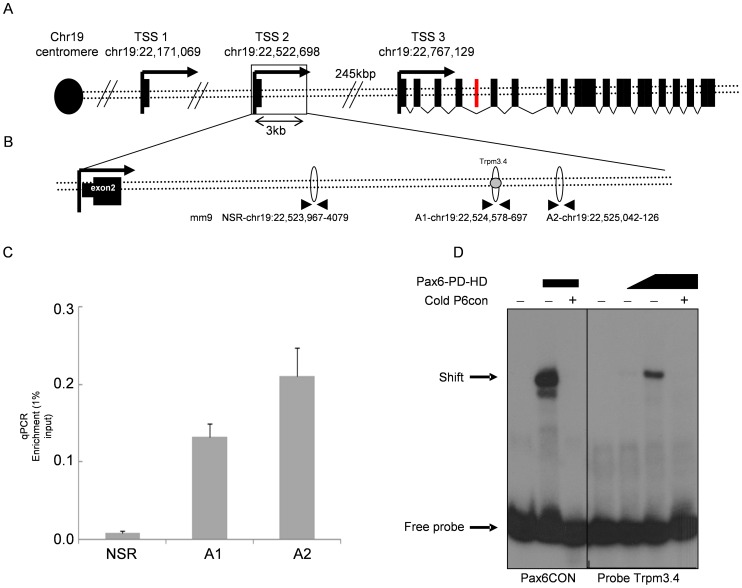
Pax6 directly binds a *Trpm3* enhancer sequence *in vitro* and *in vivo.* (A) Schematic diagram of the murine *Trpm3* locus. Three transcription start sites (TSS, black arrows) are distributed across 600 kb. The black rectangles represent *Trpm3* exons. The red rectangle represents the *miR-204*-encoding sequence in intron 6. (B) Area downstream of TSS2. Ellipses represent real-time qRT-PCR amplicons. Gray circle represents the location of EMSA probe Trpm3.4. (C) Results of real-time qRT-PCR on DNA from newborn lens ChIP experiment. Y-axis represents relative quantity of template DNA divided by the amount of template of the same reaction in 1% of input DNA. *P*-values for A1 = 0.0043, A2 = 0.0098, error bars indicate SD. (D) Acrylamide gel of radioactive EMSA probe Trpm3.4. Pax6CON is an oligonucleotide with the consensus binding site of Pax6.. For the Trpm3.4 probe, the first lane is Trpm3.4 probe only, lane 2 is probe+1∶10 flag-Pax6, lane 3 is probe+Pax6-flag, lane 4 is probe with Pax6-flag and competition by cold probe.

To determine whether Pax6 regulation of the *Trpm3/miR-204* gene is direct, we utilized chromatin immunoprecipitation (ChIP) assay to identify the binding region, in conjunction with *in-silico* TF binding site searches, followed by electrophoretic mobility shift assay (EMSA) to precisely locate Pax6 binding sites. The mammalian *Trpm3* gene contains three transcription start sites based on the RefSeq data presented in the UCSC genome browser. A preliminary ChIP-on-chip experiment performed using chromatin prepared from E13.5 whole eyes indicated Pax6 binding within the region shown in Figure 2B. To examine whether Pax6 indeed binds to these *Trpm3* regulatory sequences *in vivo*, quantitative ChIP PCR was performed on wild-type P0 lenses using anti-Pax6 antibodies. Two amplicons: A1 (chr19:22,524,578–697) and A2 (chr19:22,525,042–126) within the candidate regulatory region were enriched 16.3-fold (*P* = 0.0043) and 25.9-fold (*P* = 0.0098), respectively, compared to an upstream non-specific region (NSR-chr19:NSR-chr19:22,523,967–4079).

We tested four sequences (Trpm3.1–4) containing putative Pax6 binding sites using EMSA (prediction of target sites was performed as described in [30], EMSA described in [31], and the sequence similarities of Trpm3.1–Trpm3.4 to Pax6 binding matrices are presented in Table S2, Table S3). The probe for Trpm3.4 showed strongest binding to a flag-Pax6 protein containing both the paired domain and homeodomain (Pax6-PD-HD, Figure 2D). Increased concentrations of Pax6 protein produced stronger complex formation, while the presence of cold oligonucleotide competitors inhibited the binding (P6CON, Figure 2D). Therefore, we concluded that Pax6 directly binds *Trpm3* regulatory sequences located downstream of the second promoter of *Trpm3.*

### *miR-204* binds the 3′ UTR of *Sox11* and down-regulates its expression

Since *miR-204* is down-regulated in ocular tissues depleted in *Pax6*, we next examined which downstream genes might be regulated by *miR-204* in the developing mouse eye. Since most miRNA target sites can be found in the 3′ UTR segment of the target genes, we searched for putative *miR-204* binding sites in the 3′ UTRs of all mouse genes (TargetScan Mouse, www.targetscan.org). From the list of genes containing conserved putative binding sites for *miR-204,* 35 were found to be up-regulated in the *Pax6* mutant lens with at least a 1.5-fold change (Table 2). Statistical analysis revealed significant over-representation of miR-204 target genes among the genes negatively regulated by Pax6 (hypergeometric test, *P* = 0.019), making them good candidates for *miR-204*-mediated repression in the developing lens. Of these, *Sox11* was selected due to its reported roles in ocular development [32]. Interestingly, *Sox11* is encoded by a single exon and a long, 5,301-bp 3′ UTR, and was the only gene from this list that had three highly conserved putative *miR-204* target sequences at positions 545–551, 916–923 and 3848–3855 (TargetScan Mouse). *Sox11* is a member of the SoxC group of TFs, which are required for neurogenesis in the embryo and adult [33]–[36].

**Table 2:** Putative miR-204 targets found to be upregulated in Pax6 CKO lenses.

*Gene Symbol*	*Fold-change*	*P-value*	*Conserved sites*	*Poorly conserved sites*
Abca1	1.64	4.24E-02	1	0
Arhgap11a	2.83	1.33E-02	1	1
Bcl11a	2.88	4.53E-04	0	1
Cdc25b	1.69	1.57E-03	1	0
Chn2	1.50	1.11E-04	1	0
Cpne8	1.58	4.13E-02	1	1
Csrnp3	2.07	1.08E-02	1	0
Dtx1	1.54	4.54E-02	1	0
Efnb3	1.52	5.76E-03	1	0
Elavl3	2.04	4.86E-03	1	0
Fam126a	1.51	2.96E-02	0	1
Fbn2	2.11	2.60E-02	1	0
Gpm6a	1.79	1.53E-02	1	0
Khdrbs3	1.91	3.30E-05	1	0
Klf3	1.68	3.09E-04	1	0
Lrp2	3.21	8.46E-03	1	0
Mapt	1.67	1.88E-03	0	3
Meis1	1.82	1.87E-02	0	2
Myo10	1.91	2.07E-02	1	0
Nfia	1.87	1.09E-03	1	1
Nptx1	3.03	1.58E-02	1	0
Nrarp	1.52	5.07E-03	1	1
Ntrk2	1.78	2.69E-03	1	0
Ppic	1.72	4.39E-04	0	1
Prrx1	1.71	4.91E-02	2	0
Rgs8	1.62	6.54E-04	1	1
Satb2	1.52	1.57E-02	1	0
Sgip1	1.52	9.62E-03	0	1
Sh3pxd2a	1.84	2.52E-02	0	2
Slc43a1	1.80	1.84E-03	1	1
Sox11	2.30	2.83E-02	3	0
Tmod3	1.54	2.87E-02	2	0
Zdhhc15	1.51	1.93E-02	0	1
Zfp36l1	2.73	4.43E-04	1	0
Zfp423	1.54	3.77E-04	1	0

**Figure 3:**
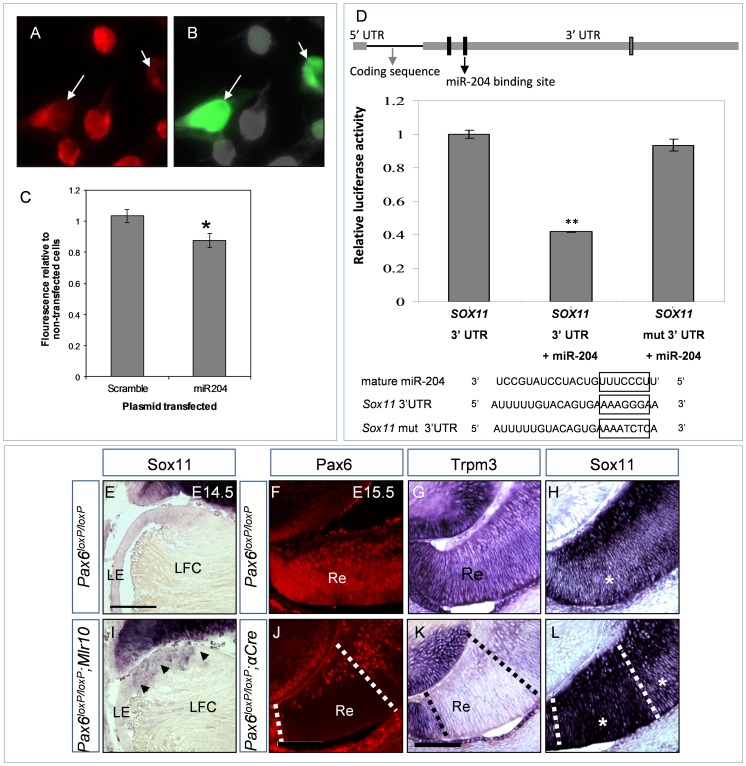
*miR-204* down-regulates the expression of *Sox11*. (A–B) Sox11 immunofluorescence (A, red) and EGFP (B, green) in Neu-2a cells transfected with *miR-204-miRVec* plasmid and pCAG-GFP. White arrows indicate GFP-positive transfected cells with visibly reduced Sox11 immunofluorescence. (C) Quantification of Sox11 immunofluorescence in cultures transfected with either *scramble-miRVec* plasmid or a *miR-204*-*miRVec* control plasmid. Y-axis is Sox11 fluorescence of transfected cells divided by that of non-transfected cells in the same image field. Error bars are SEM (*P* = 0.0104, n = 163;150 cells). (D) Illustration of *Sox11* cDNA including the untranslated and coding regions drawn in relative proportion. The three predicted *miR-204* binding sites in the 3′ UTR are indicated as rectangles. Black-labeled rectangles were tested in the luciferase reporter assay. The mutated site is marked with a black arrow. Graph presents the relative luciferase luminescence in cells transfected with wild-type *Sox11* 3′ UTR, wild-type 3′ UTR and *miR-204*, or mutated 3′ UTR and *miR-204*. Error bars represent SEM (*P* = 0.0011, n = 3). Below are alignments of *miR-204* RNA sequence, with wild-type and mutated *Sox11* 3′ UTR regions used in transfections. (E–L) Cryosections of control (E–H), *Pax6^loxP/loxP^;Mlr10-cre* (I) and *Pax6^loxP/loxP^;a-Cre* distal optic cup (J–L) stained with riboprobe against *Sox11* (E,I,H,L) or *Trpm3* (G,K) and Pax6 immunofluorescence (F,J, red). Arrowheads in (I) indicate up-regulation in the enlarged transition zone of the *Pax6^loxP/loxP^;Mlr10-cre* lens. (F–H) Adjacent sections of the same control animal: asterisk in (H) marks intermediate level of *Sox11* staining in outer retina. (J–L) Adjacent sections of the same *Pax6^loxP/loxP^;a-Cre* distal retina: dotted lines demarcate area of *Pax6* deletion, asterisks mark outer retina with elevated levels of Sox11 (H). LE, lens epithelium; LFC, lens fiber cell; Re, retina. Scale bar = 100 µm.

To examine whether *miR-204* down-regulates *Sox11* expression, we utilized mouse neuroblastoma 2a (Neu-2a) cells, which show high expression of Sox11 [37]. The cells were transfected with either a *miR-204* mimic plasmid (*miR-204-miRVec*) or a scrambled vector (*scramble-miRVec*) and were examined for Sox11 immunofluorescence. On average, cells transfected with scrambled miRNA had fluorescence levels similar to those of non-transfected cells (1∶1.03, n = 163), whereas cells transfected with *miR-204* had significantly lower levels (1∶0.87, n = 150, *P* = 0.0104, Figure 3A–3C). This was confirmed at the mRNA level in a human lens cell line (H36CE) and mouse Neu-2a transfected with a mimic *miR-204* (see below and Figure S8). Therefore, *miR-204* is able to down-regulate the level of Sox11 transcript and protein in culture.

To determine whether Sox11 down-regulation is a result of direct binding, part of the wild-type 3′ UTR of *Sox11*, containing the first two putative *miR-204* binding sites, was cloned downstream of the luciferase gene and compared to a mutated version of the 3′ UTR. Upon co-transfection of the wild-type *Sox11* 3′ UTR with *miR-204*, luciferase activity was reduced by 59% (*P* = 0.0012). In contrast, when we used a mutated version of the site that had the highest probability of conserved targeting (based on TargetScan version 6.2, position 914–921 on the 3′ UTR), luciferase levels were restored to control levels (96%, *P* = 0.20, Figure 3D arrow). Therefore, *miR-204* binds the 3′ UTR of *Sox11* and down-regulates its expression. Several miRNA binding sites on the gene's 3′ UTR usually exhibit additive effects ([38]). Yet, in the case of Sox11, mutating one site was sufficient to rescue most of the effect of miR-204. This reveals the importance of this site for the regulation of *Sox11*, although the other sites may contribute as well.

### Pax6 negatively controls *Sox11* expression *in vivo* in the lens and retina

*Sox11* has been shown to be required for ocular development in mice. In *Sox11* ^−/−^ mutant embryos, the OC fails to close, resulting in a coloboma, a folded retina and a small lens that remains attached to the cornea [32]. This phenotype is reminiscent of heterozygous *Pax6* mutations, suggesting a positive interaction between *Pax6* and *Sox11* in the context of early eye development [32]. However, we did not detect reduced expression of *Sox11* transcript in *Pax6^lacZ/lacZ^* embryos in the OV or SE on E9.5 (Figure S4). Therefore, while both *Pax6* and *Sox11* are required for lens vesicle detachment and OC morphogenesis, *Pax6* is not required for the expression of *Sox11*.

In contrast, we identified a negative regulatory interaction between *Pax6* and *Sox11* at later stages of eye development as observed by the 2.3-fold elevation in *Sox11* revealed by the microarray results above. To validate these findings, we characterized *Sox11* expression in control and *Pax6^loxP/loxP^*;*Mlr10-cre* eyes on E14.5. In the wild-type, *Sox11* transcripts were detected in the retina but not in the LE, in agreement with previous reports (Figure 3E, 3H, [32]). In contrast to previous studies, which utilized a *lacZ* reporter [32],[39], we did not detect *Sox11* transcript in the lens fiber cells (E14.5, Figure 3E). This up-regulation of the *lacZ* reporter may be due to loss of *miR-204* binding sites within the 3′ UTR, which were replaced with the *lacZ* gene, or to persistence of β-galactosidase protein in the slow-metabolizing lens fibers. *Sox11* mRNA was elevated at the equator of *Pax6^loxP/loxP^*;*Mlr10-cre* lenses (Figure 3I, arrowheads), similar to the area of elevation seen with *Sox2* [24] and *Sox9* (Figure S5) in Pax6 mutant lenses. The elevation of *Sox11* transcript in the *Pax6^loxP/loxP^*;*Mlr10-cre* lens validates *Pax6*'s negative regulation of *Sox11* in the lens.

*Trpm3/miR-204* is expressed in the progenitors of the OC and similar to Pax6, its expression is higher in the peripheral region of the OC, which is populated by the progenitors of the iris and ciliary body (Figure 1C, 1G, 1K and Figure 3G). While *Sox11* is also expressed in the OC, its expression is lowest in the iris and ciliary body progenitors (not shown), intermediate in the neuroblastic layer (NBL) and highest in the ganglion cell layer (Figure 3H). We next examined whether *Pax6* may also function in the OC to modulate *Sox11* using *Pax6^loxP/loxP^;a-Cre* embryos [29]. In the region depleted of *Pax6* (Figure 3J, dotted lines), *Trpm3* was reduced (Figure 3K, dotted lines), while in the same region, *Sox11* transcript level was markedly elevated (Figure 3L, small asterisk in area demarcated by dotted lines). The area of elevation corresponded to the *Pax6^−^;Crx^−^* population of RPCs in mutated *Pax6^loxP/loxP^;a-Cre* retinas [11].

Therefore, Pax6 down-regulates the expression of *Sox11* in progenitors of the lens and in a subclass of RPCs. Combined with the results above, these findings suggest that *Pax6* modulates *Sox11* via *miR-204*-based repression.

### Pax6 regulation of *Trpm/miR-204* is evolutionarily conserved in vertebrates

As *miR-204* was previously shown to be expressed and to regulate eye morphogenesis in medaka fish [17], we hypothesized that Pax6 regulation of medaka *miR-204* is evolutionarily conserved.

**Figure 4:**
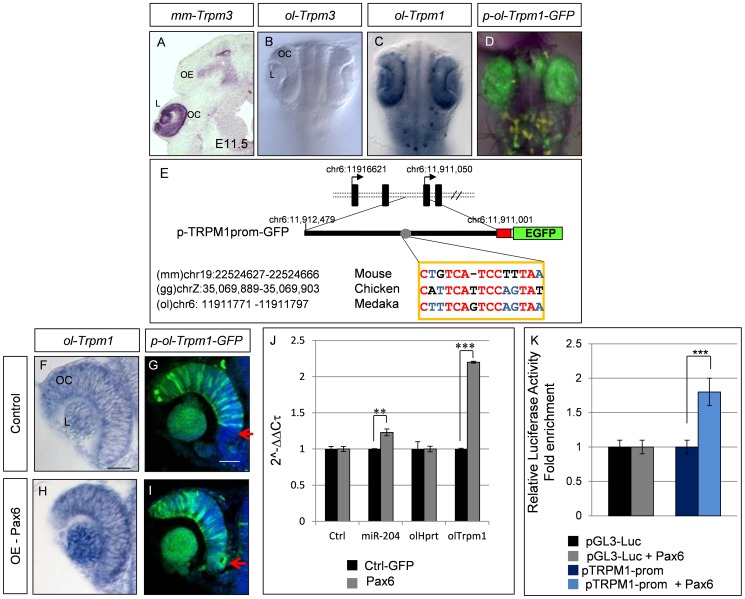
Characterization of the medaka *ol-Trpm1/miR-204* regulatory region. (A) RNA *in-situ* hybridization on frontal eye sections of E11.5 wild-type mouse embryos with the mouse *mm-Trpm3* probe. (B–D) Bright-field dorsal views of embryos at stage 24 of development; whole-mount *in-situ* hybridization with (B) ol-Trpm3 or (C) ol-Trpm1 probes and (D) epifluorescence of cI-transgenic embryos for EGF expression from *p-ol-Trpm1-GFP* transgene. (E) The *p-ol-Trpm1-GFP* construct includes 1.5 kb upstream of the coding region of *ol-Trpm1*. The red box represents the minimal TK promoter. The sequence with similarty to the Trpm3.4 Pax6-binding site is indicated. The numbers indicate respective genomic locations in the three indicated genomes. Conserved nucleotides between the three species are indicated in red or blue (two out of three analyzed species), and non-conserved nucleotides are in black. (D, G) EGFP expression in the whole (D) or a section (G) of the eye of *p-ol-Trpm1-GFP*-transgenic embryos recapitulates endogenous *ol-Trpm1* expression pattern (C,F). (H,I) *Pax6* mRNA over-expression activates both *ol-Trpm1* and *EGFP* expression in the ventral retina (red arrow) of the OC. (J) Fold-changes (expressed as 2-ΔΔCt values) in *miR-204* and *ol-Trpm1* quantified by qRT-PCR, from Pax6- compared to GFP-injected embryos. (K) Relative luciferase luminescence upon transfection of HeLa cells with Pax6 expression plasmid with or without *ol-Trpm1* promoter sequences. **P<0.001; ***P<0.0001. Abbreviations: L, Lens; OC, optic cup; OE, olfactory epithelium; NC neural crest melanocytes. Scale bar in C: 20 µm.

Interestingly, while *ol-Trpm3* is not expressed in the medaka at stage 24 (st 24), the expression of *ol-Trpm1* corresponded with the pattern of expression of murine *Trpm3* and *miR-204* (*mm-Trpm3*, Figure 1E and Figure 4A, 4C). The *ol-Trpm1* was expressed in the LP, neuroretina, ciliary marginal zone, ciliary body and presumed RPE (Figure 4C, 4F), suggesting that both *ol-Trpm3* and *ol-Trpm1* may have undergone a process of divergent gene functionalization, a common evolutionary process in teleost species [40]. To identify additional paralogs for both *ol-Trpm1* and *ol-Trpm3* genes in medaka fish, we used both available *ol-Trpm1* (ENSORLT00000009435) and *ol-Trpm3* (ENSORLT00000013467) sequences as queries to search public databases for the orthologous genomic locus. This search did not retrieve any additional *ol-Trpm1/3* genes in the medaka genome (data not shown). Furthermore, comparison of the protein sequences together with phylogenetic analysis of many known vertebrate Trpm1 and Trpm3 proteins (human, caw, mouse, rat, chicken, *Xenopus*, zebrafish, Table S6) using PHYLIP package tools [41] identified *ol-Trpm1* as more closely related to the mammalian *Trpm3* than to mammalian *Trpm1* (Figure S6). In support of these data, mouse–medaka comparative genomic analysis using MultiZ and Medaka Chain/Net package at UCSC (http://genome.ucsc.edu/) highlighted the locus for medaka *ol-Trpm1* as a possible syntenic region to the mouse *Trpm3* locus (Figure S7).

In medaka, *ol-miR-204* is located within an *ol-Trpm1* intron. Utilizing phylogenetic footprinting in sequences from related species [42], we identified putative Pax6 cis-regulatory elements within the first 1.5 kb upstream of the *ol-Trpm1* coding region (Figure 4E). This region included a Pax6 binding site that resembled the Trpm3.4 region bound by Pax6 *in vitro* (Figure 2B, Figure 4E). To test the regulatory potential of these sequences, the medaka 1.5-kb genomic fragment was amplified and fused with a nuclear EGFP reporter to generate the *p-ol-Trpm1P-GFP* construct (Figure 4E). *p-ol-Trpm1P-GFP* was then assayed for possible enhancer activity in medaka embryos [42]. Three stable transgenic *p-ol-Trpm1P-GFP* lines showed comparable and robust EGFP expression in the developing fish eye in a pattern that mimicked the reported spatio-temporal distribution of the expression pattern of both *ol-Trpm1* and *miR-204* in medaka ([17], Figure 4D, 4G), at both embryonic (Figure 4C, 4D) and adult (not shown) stages. We thus concluded that this region contains the sequences that mediate *Trpm1/miR-204* expression in the medaka fish.

To examine whether Pax6 controls *ol-Trpm1/miR-204* expression *in vivo*, we tested whether over-expression could expand *ol-Trpm1/miR-204*'s expression domains. Injections of murine *Pax6* mRNA (75–100 ng/µl) in *p-ol-Trpm1P-GFP-*transgenic embryos expanded and enhanced EGFP expression in both the lens and ventral retina of the OC (stage 24; Figure 4I, red arrow). Notably, no ectopic EGFP expression was observed in regions other than the retina, supporting tissue-specific Pax6-mediated activation of *ol-Trpm1/miR-204* expression. Likewise, Pax6 over-expression in wild-type embryos expanded *ol-Trpm1* mRNA distribution (Figure 4H) and increased both *miR-204* and *ol-Trpm1* transcript levels as detected by quantitative (q) RT-PCR (Figure 4J). In agreement with these results, transient transfection of HeLa cells with murine *Pax6* activated a luciferase reporter construct containing the *Trpm1P* fragment significantly more than the luciferase activity observed in controls (Figure 4K). Collectively, these findings revealed that Pax6 is able to regulate the *Trpm1/miR-204* promoter in fish, similar to its regulation of *Trpm3/miR-204* in mice.

### *miR-204* mediates Pax6 suppression of *Sox11* and additional genes in the lens and in medaka embryos

**Figure 5:**
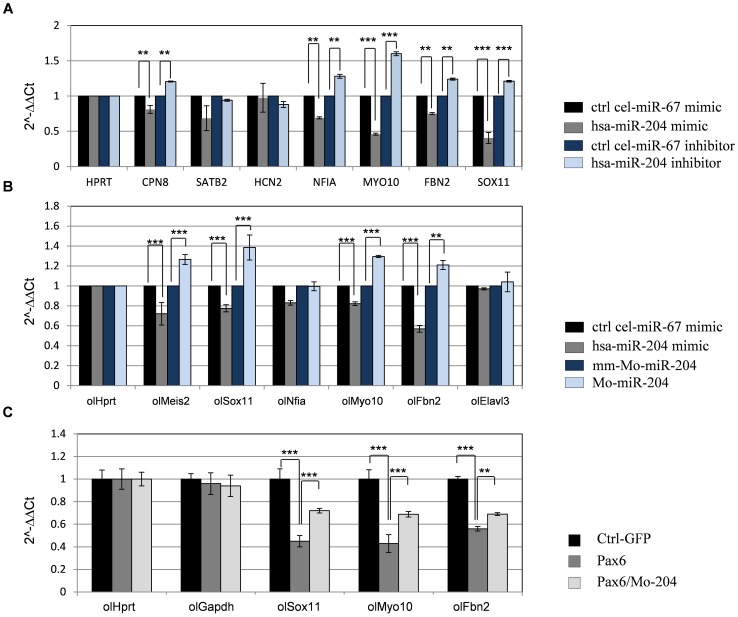
*miR-204* affects expression of multiple genes and mediates Pax6 suppression of gene expression. (A) H36CE cells transfected with *hsa-miR-204 mimic* or control miRNA (*cel-miR-67 mimic*) or *hsa-miR-204 inhibitor* or control miRNA inhibitor (*cel-miR-67 inhibitor*). Significant reduction following over-expression of *hsa-miR-204 mimic* and significant elevation following transfection with *hsa-miR-204 inhibitor* was detected by qPCR (expressed as 2-ΔΔCt values) for transcripts of *Sox11*, *Cpn8*, *Nfia*, *Myo10* and *Fbn2*. Error bars are SD (**P<0.001 and ***P<0.0001, n = 3). (B) Fold-change (expressed as 2-ΔΔCt values) in mRNA levels of indicated medaka genes quantified by qRT-PCR, from stage 24 embryos injected with *hsa-miR-204 mimic* compared to control *cel-miR-67mimic*, morpholino against miR-204 (*Mo-miR-204*) and mismatched morpholino (*mm-Mo-miR-204*). 400 embryos were pooled for each assay, and technical triplicate experiments were independently executed at least three times. (C) Fold-change in mRNA levels of the indicated genes quantified by qRT-PCR from embryos injected with *Pax6*, *Pax6/Mo-miR-204* and pGFP-expressing plasmid as a control. Results are shown as means ± SD, 250 embryos pooled for each assay. Technical triplicate experiments were independently executed at least three times, n = 3, **P<0.001, ***P<0.0001.

To further evaluate the contribution of *miR-204* to the phenotype observed following loss of *Pax6*, we tested the response of a subset of seven additional genes (*Cpn8, Satb2, Hcn2, Nfia, Myo10, Fbn2,* and *Elavl3*) that were both up-regulated in *Pax6*-negative lenses and contained putative *miR-204* binding sites in their 3′ UTR (Table 2) to over-expression of *hsa-miR-204* mimic. Most of these genes were examined in the human lens H36CE cell line, whereas *Elavl3*, a member of the Hu family of neuronal RNA binding proteins, was examined in mouse Neu-2a cells (Figure S8) because of its low expression levels in the lens cell line (data not shown). From the eight genes that were tested, including *Sox11*, six were down-regulated in response to *hsa-miR-204* mimic; the five reduced in H36CE cells were also significantly elevated following transfection with *hsa-miR-204* inhibitor (*Cpn8, Nfia, Myo10, Fbn2, Sox11*; Figure 5A). In Neu-2a cells, *Elavl3, Sox11* and *Myo10* were significantly reduced (Figure S8). Luciferase assay conducted on the *Elavl3* 3′ UTR further supported direct regulation of *Elavl3* by *miR-204*, and antibody labeling revealed expansion of *Elavl3* expression to the posterior lens cells of *Pax6^loxP/loxP^;Mrl10-Cre* mutants (Figure S8, [43]).

As the expression pattern and regulation of *miR-204* seem to be conserved between fish and mammals ([17], Figure 4), we also expected conservation of *miR-204* activity. To test this, medaka embryos were injected with either *hsa-miR-204* mimic for over-expression of miR-204, or with a morpholino (*Mo-miR-204*) for knock- down of the endogenous *hsa-miR-204* [17]. Control embryos were injected with either an unrelated control *Caenorhabditis elegans cel-miR-67* mimic or a six-base mismatched morpholino (*MM-Mo-miR-204*). The expression of *ol-Sox11,* o*l-Nfia, ol-Fbn2, ol-Myo10,* and *ol-Elavl3* was monitored by qRT-PCR in embryos collected at stage 24, when the ocular phenotype of *ol-miR-204* was first documented (Figure 5B, [17]). *Ol-Meis2* was also monitored as it is a known target of *miR-204* in medaka [17]. *ol-Meis2, ol-Sox11*, *ol-Fbn2* and *ol-Myo10* transcripts were significantly reduced following treatment with *hsa-miR-204* mimic, by 28±3.7%, 23±2.8%, 43±3,4% and 21±1% relative to the control, respectively (Figure 5B); these genes were up-regulated following treatment with the *Mo-miR-204* by 26.5±4.9%, 38.5±7.8%, 21±4% and 29.4±1.1%, respectively, relative to the control (Figure 5B). This finding supports the regulation of *ol-Sox11* as well as *ol-Myo10,* and *ol-Fbn2* by *miR-204* in medaka, similar to the regulatory interaction observed in mammals.

We next examined Pax6 regulation of *ol-Sox11*, *ol-Fbn2* and *ol-Myo10.* Injection of mouse Pax6 mRNA into medaka embryos resulted in reduced expression of these three genes compared with control embryos injected with EGFP mRNA (ol-Sox11 was reduced to 45±5.1%, ol-Myo10 was reduced to 43±6.3%, and ol-Fbn2 to 56.4±2.1% Figure 5C). This reduction was significantly alleviated when embryos were co-injected with *Mo-miR-204* (*ol-Sox11* was rescued by 28.5±1%, *ol-Myo10* by 26±3%, and *ol-Fbn2* by 18.4±1.4%) (Figure 5C), indicating that at least part of the repression is mediated through *miR-204* activity.

Collectively, and similar to mammals, the expression of *ol-Sox11*, *ol-Fbn2* and *ol-Myo10* is negatively regulated by *Pax6* and *ol-miR-204* in medaka fish. These findings reveal several novel targets of *miR-204* in the lens and demonstrate how Pax6 direct control of *miR-204* can simultaneously inhibit multiple genes, thus tightly regulating normal lens fate and physiology.

## Discussion

In this paper we establish *Pax6* as an indirect negative regulator of gene expression through *miR-204*. We demonstrate that Pax6 regulation of *miR-204* is conserved in vertebrates through regulation of the host gene *Trpm3* in mice and *ol-Trpm1* in medaka. The study identifies several targets of *miR-204* repression in the lens, among them *Sox11*, an important factor for eye and CNS development. Additional new targets for *miR-204* include neuronal factors and genes involved in cell motility. Pax6 regulation of *miR-204* explains part of the complex and divergent inhibitory activity of *Pax6* in ocular progenitor cells, which is required to establish and maintain the identity of ocular tissues.

### *miR-204* in ocular physiology and development

In mammals, *miR-204* has a closely related paralog the *miR-211*. *miR-204* and *miR-211* differ by one or two nucleotides, depending on the species. However, they have the same seed-region sequence. Classified as a subfamily of miRNAs, they show the same set of predicted targets (TargetScan [12]). Interestingly, *miR-211* first appears in mammals through the evolution of one of the two copies of miR-204, which is present in two identical copies in the genomes of early vertebrates and fish, including medaka fish [17]. In future studies it would be of interest to distinguish the different expression pattern of Trpm1/3 paralog genes and the functional activity of their hosted miRNAs in the different lineages.

The roles of *miR-204* in the eye have been recently examined using a primary culture of human fetal RPE. In those cells, *miR-204* was shown to down-regulate levels of *TgfbR2* and *Snai2*, genes known to be involved in the epithelial-to-mesenchymal transition (EMT, [18]). The authors provided evidence of this regulation helping to maintain epithelial phenotype and prevent EMT of the RPE [18]. EMT is characteristic of TGFβ-induced anterior subcapsular cataract formation, a pathology also associated with reduced dosage of Pax6 [44]–[46]. Interestingly, secondary cataract has recently been associated with alterations in miR-204 expression [47]. With this in mind, it is possible that part of *Pax6*'s activity in inhibiting cataract formation is mediated by the activity of *miR-204* in the lens and that restoration of *miR-204* levels may prevent anterior subcapsular cataract formation.

**Figure 6:**
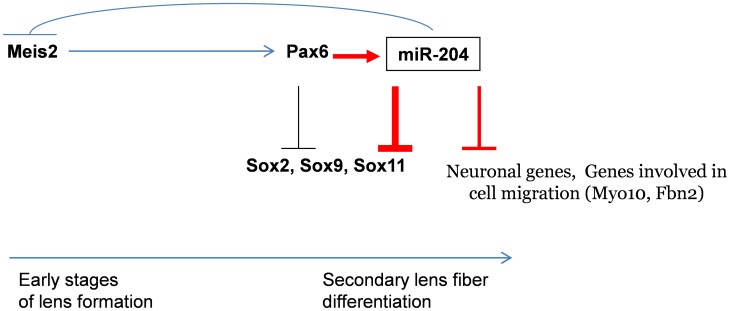
Model of Pax6 genetic regulation of *Sox11* during ocular development. Pax6 directly regulates *miR-204* by binding and activating expression of the host genes *Trpm3* in mammals and *ol-Trpm1* in fish. During later stages of lens development, *miR-204* reinforces inhibition of *Sox11*. *miR-204* is upstream of several genes involved in neurogenesis and cell motility. The findings in medaka suggest that during the early stages of LP formation, *ol-miR-204* and *Pax6* may co-regulate each other via a negative feedback loop through the established *Meis2*-Pax6 pathway. Red arrows indicate new data presented here.

In addition, the novel *miR-204* targets in lens development uncovered here include genes involved in cell motility *Myo10* [48], cell matrix and regulation of the TGFβ pathway *Fbn2* [49], and genes that function in neuronal or glia cells *Sox11* [37], *Elavl3* [43], *Cpne8* [50] and *Nfia* [51]. The identification of multiple genes responding to *miR-204*, and the finding that some of these targets are conserved among vertebrates (*Sox11, Fbn2, Myo10*), suggest a major effect of miR-204 in regulating LE fate and cell behavior. Future studies should evaluate the contribution of each of the identified targets to maintaining lens physiology (Figure 6). Furthermore, as miR-204 is expressed in several epithelial tissues that originate from the neuroectoderm, including the ciliary body, iris and choroid plexus, it is possible that miR-204 plays a more general role in maintenance of a non-neuronal fate of various epithelia within the CNS.

*miR-204* was found to negatively regulate the expression of *ol-Meis2* in the eye of medaka fish [17]. Murine *Meis1* and *Meis2* have been found to be upstream regulators of *Pax6* during induction of the LP [52]. It was therefore proposed that *miR-204* regulates *ol-Pax6* in the lens through regulation of *ol-Meis2* [17]. The findings presented here suggest a negative feedback loop between *Meis1/2, Pax6* and *miR-204* (Figure 6). This regulatory loop may only be relevant at early stages of lens development, because in the mouse lens, *Meis1* and *Meis2* expression is lost before E12.5 [53], whereas *Pax6* and *miR-204* expression is maintained throughout development (Figure 1). Further analysis is required to substantiate the relevance of the *miR-204–Meis1/2–Pax6* feedback loop in maintaining correct Pax6 dosage during early stages of lens development.

### Pax6 regulation of neuronal SOX genes

A previous global analysis of gene expression revealed a significant number of neuron-specific genes that are up-regulated in *Pax6*-heterozygous lenses [31]. This raised the hypothesis that *Pax6* has a dual role: promoting lenticular genes, while simultaneously suppressing competing programs, such as neural development [31]. The altered expression profile in the *Pax6*-null embryonic lens reported here further revealed over-representation of neurogenic genes using several tools for seeking gene-ontology enrichment *DAVID*, (NIH, [54]), *Expander* (Tel-Aviv University, [55]), *GATHER* (Duke University, [56]) and *Ontologizer2.0* (Computational Biology Group, [57]). Among these, four SOX members—*Sox2, Sox6, Sox9* and *Sox11*—were up-regulated upon *Pax6* deletion. All four are required for nervous system development [33], [34], [58]–[64]. Three of these genes were found to be regulated by *Pax6* at the late stages of lens development *in situ*: *Sox2* [24], *Sox11* (Figure 3) and *Sox9* (Figure S5). The propensity for *Pax6* to repress SOX genes in the lens could be a way of silencing competing genetic programs.

In this paper, we focused on the regulatory interaction between *Pax6* and *Sox11* to determine the mechanism underlying *Pax6* inhibitory activity during organ formation. In the eye, *Sox11* is required for normal development of both lens and retina [32]. The expression of *Sox11* in the lens overlaps with that of *Pax6* during the early LP stage but is reduced to below detection levels by *in-situ* hybridization (ISH) during the lens vesicle stage (E11, not shown). In contrast, *Pax6* expression is maintained in the LE through adulthood. This expression pattern supports negative regulation of *Sox11* by *Pax6* from the lens vesicle stage onward. The similar phenotypes of *Sox11* and *Pax6* mutants suggest that during early stages of LP formation, *Pax6* is a positive regulator of *Sox11* [32]. However, our analysis of *Sox11* expression in *Pax6^lacZ/lacZ^* mutants does not support this notion (Figure S3). The current study provides evidence for inhibition of *Sox11* by *Pax6*, mediated by direct *miR-204* repression from the stage at which *Trpm3/miR-204* expression begins (Figure 1). We propose that *Pax6* initially functions in parallel to *Sox11* for growth and morphogenesis of the LP. At later stages, *Pax6* is required for the initiation of *Trpm3*/*miR-204* expression in both retinal and lens progenitors. In cells that express *miR-204*, the regulation of *Sox11* by *Pax6* becomes inhibitory, contributing to loss of Sox11 expression from the lens fibers (Figure 3 and Figure 6). This is further supported by the observation that over-expression of either *Pax6* or *miR-204* in medaka, results in reduced expression of *Sox11*, and that Pax6 inhibition can be rescued by knock-down of miR-204 (Figure 5). Hence, this study reveals a new level of regulatory hierarchy mediated by *Pax6* during the acquisition of cell fate.

Combinatorial interactions between miRNAs and TFs have been suggested based on *in-silico* strategies which identified a prevalent TF-miRNA feed-forward loop in which miRNA and TFs co-regulate target genes, as well as each other [65], [66]. We therefore considered the possibility that Pax6 might negatively regulate *Sox11* transcription directly, in addition to repressing expression through *miR-204*. We did not, however, obtain substantial evidence for this possibility when examining the effects of Pax6 on *Sox11* regulatory regions in cell culture (HeLa and 293T cells, D. G., and R. A-P., data not shown). Nevertheless, it is possible that binding sites, other than those tested here, play a role in the regulation of *Sox11* by Pax6, and that Pax6 represses *Sox11* in the presence of co-factors which are only available in the restricted developmental context of the embryonic lens *in vivo*.

In the retina, *Sox11* exhibits a dynamic pattern of expression. It is expressed in the OV and later in the inner OC. Sox11 levels are lower in RPCs than in post-mitotic neurons in the ganglion cell layer (Figure 3), in accordance with its importance in neurogenic precursors in the CNS along with Sox4 [33], [58]. It is worth noting that while *Sox11* is detected in the ganglion cell layer during embryogenesis it is subsequently reduced and is not detected in the postnatal retina (K. G, R. A-P unpublished observation). *Pax6* however, is maintained in terminally differentiated ganglion and amacrine cell types. Pax6 may therefore contribute to the down-regulation of Sox11 in neuronal precursors during their terminal differentiation. *Pax6* is known to play a dual role in RPCs—down-regulating the cone-rod homeobox gene *Crx* and maintaining proliferation in the peripheral retina, while maintaining pluripotency of the more central cells of the OC [11], [29]. Therefore, *Pax6* regulation of *Sox11* during retinogenesis is expected to be part of a larger regulatory network, and may be repressed by *miR-204* in only a subset of RPCs.

In conclusion, this study reveals novel involvement of miRNA-based gene repression during lens and retinal development. *Pax6*, a regulator of multiple processes during eye development, is shown to execute part of its gene regulation through direct activation of *miR-204*.

## Materials and Methods

### Mouse lines

The mouse lines employed in this study have been previously described: *Pax6^loxP^* [67], *Mlr10*-*cre* [68], *αCre* [29], *Pax6^lacz^* [69]. All animal work was conducted according to national and international guidelines and approved by the Tel Aviv University review board.

### Statistical analysis

All data were examined using two-tailed student's t-test unless otherwise stated.

### Microarray analysis

*Pax6^loxP/loxP^*;*Mlr10-cre* and *Pax6^loxP/loxP^* E14.5 lenses were dissected; 20 lenses from each litter were pooled, resulting in three control (*Pax6^loxP/loxP^*) and three *Pax6^loxP/loxP^*;*Mlr10-cre* samples. RNA purification was performed using Qiashredder and RNeasy (Qiagen). Total RNA (300 ng) was used to generate sense-strand cDNA, which was fragmented, biotin-labeled and hybridized to Affymetrix GeneChip 1.0ST microarrays. Microarray analysis was performed using Partek Genomics Suite (Partek Inc., MO, USA; www.partek.com). Differentially expressed genes with *P*-values lower than 0.05 and with a fold-change cutoff of 1.5 are listed in Table S1.

### MiRNA–target enrichment analysis

We calculated the significance of miR-204-target enrichment among the up-regulated genes (fold change >1.5, *P*-value<0.05, n = 754 genes) using TargetScan Mouse predictions (version 5.2; http://www.targetscan.org/mmu_50/) and the hypergeometric test. The background dataset for the calculation included the differentially expressed genes (fold-change cutoff >1.5 and *P*-value<0.05, n = 1,013 genes). *P*-values were corrected using the false discovery rate (FDR) method [70].

### Immunofluorescence and ISH on sections

Immunofluorescence analysis was performed on 10-µM paraffin sections or 14-µM frozen sections as described previously [67], using the following primary antibodies: rabbit anti-Pax6 (1∶400, Covance, # prb-278b), rabbit anti-Sox9 (1∶200, Chemicon, ab5535), mouse anti-Elavl3 (1∶200, Invitrogen, A21272) and goat anti-CrystallinαA (1∶1,000, Santa Cruz, sc-22389). Secondary antibodies were conjugated to alexa594 donkey anti rabbit/goat (1∶1000, Invitrogen, A-21207/A-11058) or alexa488 donkey anti mouse/goat (1∶1000, Invitrogen, A-21202/A-11055).

mRNA ISH was performed as described previously [71]. Plasmids for antisense transcription were: *Trpm3* [21] and *Sox11*—kindly donated by Kirsten Kuhlbrodt [72]. For miRNA ISH, hsa-miR-204 miRCURY LNA Detection probe (working concentration 1/150 µM/µl, Exiqon, cat number 88076-15) were hybridized to frozen sections as described previously [73].

### EMSA

HEK293T cells were transfected with p3Xflag-CMV-10 conjugated to the first 270 amino acids of Pax6 containing PD and HD binding domains. Nuclear extracts were obtained as previously described [74]. Nuclear extract (1 µl) or 1∶10 diluted nuclear extract was incubated for 10 min on ice in 8.5 mM HEPES pH 7.9, 30 mM KCl, 1.5 mM MgCl_2_, 0.4 mM DTT and 2 µg polydI/dC (Sigma). Binding with 1 µl double-stranded 5′-γ-ATP-labeled probe (30,000 cpm) was performed at room temperature for 20 minutes and 200 ng of “cold” Pax6 consensus site was used for competition (P6CON; Wolf et al., 2009). EMSA probes are listed in Table S2.

### Quantitative ChIP

Groups of 400 pooled newborn lenses were fixed in 1% formaldehyde for 10–15 minutes at room temperature. The cross-linking reaction was quenched with 0.125 M glycine. The tissues were sonicated in 4 ml lysis buffer (1 mM EDTA pH 8, 0.5 mM EGTA pH 8, 10 mM Tris-HCl pH 8) by Fisher Sonic Dismembrator Model 500 for 14 min (30 s on/30 s off, amplification = 30%). Aliquots of the sheared chromatin prepared from 40 lenses were incubated with 5 µg rabbit anti-Pax6 antibody (Millipore, Cat# AB2237, epitope C-terminus) bound to 20 µl protein G-coated magnetic beads (Invitrogen). The immunoprecipitates were washed three times and resuspended in a buffer containing 10 mM Tris–HCl pH 8.0, 100 mM NaCl, and 25 mM EDTA supplemented with 0.1 mg/ml RNaseA and 0.2 mg/ml proteinase K. After 2 h incubation at 55°C, the cross-linking was reversed by overnight incubation at 65°C. Genomic DNA was eluted into 250 µl of water using QIAquick Spin Gel Purification kit (Qiagen).

Amounts of each specific DNA fragment were determined by real-time qPCR using a standard curve generated for each primer set with 0.04, 0.2, and 1% input DNA samples. The copy number of each DNA fragment was compared to the copy number of that fragment in the input DNA. A control antibody (rabbit IgG, Millipore, Cat# NI01) was included for each set of qPCR experiments. The PCR products were between 80 and 130 bp in length. Primers used for PCR are listed in Table S2. The reactions were analyzed using a 7900 ABI PRISM PCR Instrument and 2× SYBR mix (Applied Biosystems). The parameters were: 95°C/10 min followed by 45 cycles of 94°C/10 s, 60°C/20 s and 72°C/30 s.

### Immunofluorescence quantification of protein levels

For Sox11 repression by *miR-204*, Neuro-2a cells (ATCC) were grown on coverslips in 24-well plates for 24 h and then co-transfected with 500 ng of pCAG-GFP expression plasmid and either 500 ng of scrambled-miRVec or *miR-204*-miRVec expression plasmid containing the pre-miRNA of *miR-204* [75]. Cells were fixed for 5 min in 4% (v/v) paraformaldehyde and then immunolabeled as described previously [37] using goat anti-Sox11 (1∶100, Santa Cruz) and rabbit anti-GFP (1∶500, Rockland Immunochemicals). Cell immunofluorescence was measured using ImageJ (NIH) as described previously [24]. Each pCAG-GFP-positive cell (green channel), co-transfected with either *miR-204*-miRVec or scrambled-miRVec, was measured for Sox11 fluorescence intensity (red channel). The fluorescence level of each transfected cell was divided by the average (red) fluorescence of non-transfected cells in the same field, resulting in a Sox11 index level for each cell calibrated for random image conditions. Sox11 index levels of all *miR-204-*miRVec-transfected cells were then compared to those of scrambled-miRVec-transfected controls. Fluorescence was measured blindly for the *miR-204-*miRVec- or scrambled-miRVec-transfected cells. In all measurements, the cells that had a mega-nucleus or multiple neurites were considered differentiated and were therefore not counted.

### Teleost sequence analysis and plasmid construction

The available teleost *Trpm1* genomic sequences were retrieved (http://genome.ucsc.edu/) and aligned for putative regulatory modules on the basis of sequence conservation [42]. A 1.5-kb region of *ol-Trpm1* genomic sequence containing the start codon using the primers: *ol-Trpm1* forward 5′-TGTATCATGAGCCGCTAATG-3′ and *ol-Trpm1* reverse 5′-GCAGCACCAGGAGAAGGCTC-3′, was cloned in frame with an EGFP reporter gene into the pSKII-ISceI-EGFP and pGL3 basic vectors [76] to create the *Trpm1P:GFP and Trpm1:Luc* constructs.

### Phylogenetic analysis

Sequence alignments were performed using the VISTA [77] and Multalign programs [78], which are available at the corresponding websites: [http://genome.lbl.gov/vista/index.shtml and [http://bioinfo.genopole-toulouse.prd.fr/multalin/multalin.html]. The criterion used for comparison was a minimum 75% nucleotide identity with a window size of over 100 bp. Phylogenetic analysis was performed using the PHYLIP package and the results were plotted using the Tree-view software package as previously described [41]). The syntenic genomic region was analyzed using MultiZ and Medaka Chain/Net package at UCSC (http://genome.ucsc.edu/).

### Establishment of transgenic fish lines

The Cab inbred (CI) medaka strain was used throughout this study and aged with Iwamatsu staging [79]. Transgenesis and live EGFP monitoring was performed as described previously [42]. Three independent stable transgenic lines were generated for the tested construct.

### mRNA injection of medaka embryos

*In-vitro* synthesis of mouse *Pax6* mRNA (cDNA from [80]) was performed as described previously [76]. *Pax6* mRNA was injected at 50–200 ng/µl to observe dose-dependent phenotypes. Selected working concentrations were 75–100 ng/µl. Control embryos were injected with 15 ng/µl of EGFP mRNA [76].

### Cell transfection and luciferase assays

For miRNA-repression studies (Figure 3D), a 506-bp fragment of the *Sox11* 3′ UTR (spanning two miR-204 binding sites) or 484 bp of *Elavl3*'s 3′ UTR was amplified by PCR from mouse brain cDNA and XhoI–NotI restriction sites were added with the following primers: Sox11 (F) 5′-ACACTCGAGCTGTTACTCTAGGGAGTTGA-3′ and (R) 5′-AAGGTCAAGCGGCCGCAAAGGGAAGAAGTGCCTGAA-3; Elavl3 (F) 5′-ACACTCGAGCAATGGTGCCCTACTCAGG-3′ and (R) 5′-AGGTCAAGCGGCCGCTTCCTGTGGCCATGTTTGCT-3′.

The 3′ UTRs were cloned downstream of the Renilla luciferase reporter (psiCHECK™-2, Promega). For site-directed mutation, the miR-204 binding site on the 3′ UTRs (second binding site for *Sox11*), was mutated by PCR with PfuUltra II Fusion HS DNA Polymerase (Genex). The mutagenesis primers were: Sox11 (F) 5′-TTTGTACAGTGAAAATCTCACAATCTTGCTGTGT-3′; Elavl3 (F) 5′-CTATTTTTGTAAAAACTCCAAAAGACCTCGTGGA-3′ and complementary reverse primers. Products were then incubated with DPN1 (New England BioLabs) for digestion of the source plasmid. Co-transfection of miRVec-miR-204 [75] and the Renilla-3′ UTR plasmid was in HEK293T cells with TransIT-LT1 Transfection Reagent (Mirus). After 48 h, firefly and Renilla luciferase activities were measured using the Dual-Luciferase Reporter Assay System.

Regulation of promoter activity in cell culture (Figure 4K) was performed in HeLa cells. Cells were co-transfected with the construct (100 ng), expression vector pcDNA3/Pax6 (100 ng) and RL-TK plasmid with Renilla luciferase (10 ng) as a transfection-efficiency control. Transfection was performed using Fugene Transfection Reagent (Roche) following the manufacturer's protocol [76]. Cells were harvested 48 h after transfection. Reporter activities were measured using the Dual-Luciferase Reporter Assay System (Promega). Each assay was performed in duplicate, with three biological repeats.

For functional studies of miR-204 in cell culture (Figure 5A, Figure S8) the human lens cell line H36CE (Porter et al. 1998) was transfected and qRT-PCR experiments were performed as described [81]. H36CE cells were transfected with either 50 nM miRIDIAN™ Dharmacon microRNA Mimics (hsa-miR-204 mimic or control cel-miR-67) or 80 nM miRIDIAN™ Dharmacon microRNA Inhibitor (hsa-miR-204 inhibitor or negative control cel-miR-67). Neu-2a cells were transfected using HiPerFect (Qiagen) with hsa-miR-204 mimic (Ambion) or miR negative control (Applied Biosystems) oligonucleotide. The isolation of RNA and qRT-PCR for detection of miR-204 were as previously described [82]. Total cDNA was generated using SuperScript III (Invitrogen) and qRT-PCR was performed with FastStart Universal SYBR Green Master (Roche). Primers used for qRT-PCR are listed in Table S4 and sequences of for functional studies of miR204 are listed in Table S5.

### qRT–PCR analysis of gene expression in injected embryos

At least 250 embryos were pooled in each assay. cDNA synthesis was performed using the SuperScript III First-Strand Synthesis System for RT-PCR using random hexamers (Invitrogen). *ol-Hprt* was used as the endogenous control as previously described [17]. Detection of *miR-204* was performed by TaqMan MicroRNA Assays (Applied Biosystems) following the manufacturer's protocol. Each assay was performed in duplicate. Primers used for qRT-PCR are listed in Table S4.

## Supporting Information

Figure S1 Volcano plot demonstrating differences in gene expression between Pax6^loxP/loxP^;Mrl10-cre and control E14.5 lens. Each of the 1,013 differentially expressed genes is represented by a single dot. *P*-values are presented as -log10 values, expression differences are presented as log2 fold-changes; 754 transcripts were up-regulated and 259 were down-regulated in the Pax6-depleted E14.5 lens.
(TIF)

Figure S2 *Trpm3* and *miR-204* are co-expressed in the embryonic eye. The expression pattern of *Trpm3* (A,B) and *miR-204* (C) detected by *in-situ* hybridization on a sagittal section of E12.5 head (A) and P0 optic cup (B,C). Abbreviation: CP, choroid plexus; IE, inner ear; L, lens; PE, pigmented epithelium.
(TIF)

Figure S3 Reduced expression of *Trpm3* and *miR-204* in progenitors of iris and CB in Pax6 somatic mutants. *Trpm3* (A,B) and *miR-204* (C,D) in the inner and outer layers of the developing ciliary body and iris of *Pax6^loxP/loxP^* control (A,C) but not in the outer layer of the distal optic cup of *Pax6^loxP/loxP^;Dct-Cre* mutants (P5). The outer layer of the optic cup is marked with arrows.
(TIF)

Figure S4 The expression of Sox11 is maintain in Pax6 systemic mutants. *Sox11* expression detected by *in-situ* hybridization in control (A,B) and *Pax6^lacZ/lacZ^* (C,D) mutant eye on E9.5 (A,C) and E11.5 (B,D). Abbreviations: CE, corneal epithelium; LV, lens vesicle; OC, optic cup; OM, ocular mesenchyme; OR, ocular rudiment; PE, pigmented epithelium.
(TIF)

Figure S5 Sox9 is elevated in Pax6 deficient lens. *Sox9* detected with antibodies (green) in the *Pax6^loxP/loxP^;Mrl10-Cre* (B) but not control *Pax6^loxP/loxP^* (A) lens on E14.5.
(TIF)

Figure S6 Phylogenetic analysis of the *Trpm1* and *Trpm3* genes. Phylogenetic tree comparing the amino acid sequences of the Trpm1 and Trpm3 members. Complete or partial protein sequences of all *Trpm1* and *Trpm3* genes were obtained from the NCBI protein database (Table S4). Genes displaying the greatest sequence similarities cluster together; branch length is proportional to divergence (percentage of amino acid changes). The numbers indicate the bootstrap confidence for each node (n = 1,000).
(TIF)

Figure S7 Comparison of the genomic organization between mouse *mm-Trpm3* and medaka *ol-Trpm3* loci. (A) Schematic representation of the *Trpm3* locus in the mouse genome obtained from the UCSC database. (B) Vista comparison of the mouse genomic region, containing the Trpm3.4 box, plotted against those of human, *R. norvegicus*, rhesus, dog, and horse. (C) Schematic representation of the *Trpm1* locus in the medaka genome obtained from the UCSC database. (D) Vista comparison of the medaka genomic region, containing the Trpm3.4 box, plotted against those of fugu, tetraodon and zebrafish. Sequences conserved among the species (60% identity over 100 bp) are indicated in pink. Evolutionarily convergent genes are indicated with red boxes.
(TIF)

Figure S8 *Elavl3* is regulated by miR-204 in Neu-2a cells. (A) Neu-2a cells transfected with miR-204 mimic or scrambled miRNA as a control. *Elavl3*, *Sox11* and *Myo10* were significantly down-regulated to 77%, 86% and 84%, respectively. Error bars represent SEM (*P<0.01, n = 7). (B) Relative luciferase luminescence in HEK293T cells transfected with wild-type *Elavl3* 3′ UTR, or mutated *Elavl3* 3′ UTR co-transfected with a plasmid containing the pre-miR-204. Error bars represent SEM (*P*<0.0005; n = 3). Below is the alignment of the *miR-204* RNA sequence, wild-type *Elavl3* 3′ UTR and mutated *Elavl3* 3′ UTR. (C) Elavl3 immunofluorescence of E14.5 control (D) and *Pax6^loxP/loxP^;Mlr10-cre* mutant lens. White arrowheads mark expanded equatorial region. Scale bar = 100 µM.
(TIF)

Table S1 The list of 1,013 differentially expressed genes following Pax6 loss. The transcriptom of the *Pax6^loxP/loxP^*;*Mlr10-cre* E14.5 lenses was compared with that of control *Pax6^loxP/loxP^* using the Affymetrix platform. The analysis was performed using Partek Genomics Suite. Differentially expressed genes with *P*-values lower than 0.05 and with a fold-change cutoff of 1.5 are listed.
(DOCX)

Table S2 List of oligonucleotide probes used for ChIP and EMSA.
(DOCX)

Table S3 Selection of putative Pax6 binding sites. Identification of putative Pax6 binding sites within the genomic region which is bound by Pax6 based on ChIP assay (mm9- chr19:22,524,578- Chr19:22,525,126). The putative Pax6 binding motifs were selected based on the sites identified using SELEX assay (Xie and Cvekl 2011, Epstein et al., 1994). The sites with up to 3 mismatches compared to the motifs were selected using fuzznuc program (http://mobyle.pasteur.fr/cgi-bin/portal.py?#forms::fuzznuc). The patterns and number of mismatch that were identified for each of the putative binding sequences are presented.
(DOCX)

Table S4 Primers used for detection of transcript levels of specific genes using q-PCR.
(DOCX)

Table S5 Sequences employed for functional studies of miR-204 in cell culture and in fish embryos.
(DOCX)

Table S6 The protein sequences of the Trpm1 and Trpm3 orthologues from various species for phylogenetic analysis of the *Trpm1* and *Trpm3* genes (Figure S6).
(DOCX)
