# Supplementary figures and images for: Pax6 Regulates Gene Expression in the Vertebrate Lens through miR-204
Source: PLoS Genet. 2013 Mar 14;9(3):e1003357. doi: 10.1371/journal.pgen.1003357 (PMC3597499; doi:10.1371/journal.pgen.1003357)

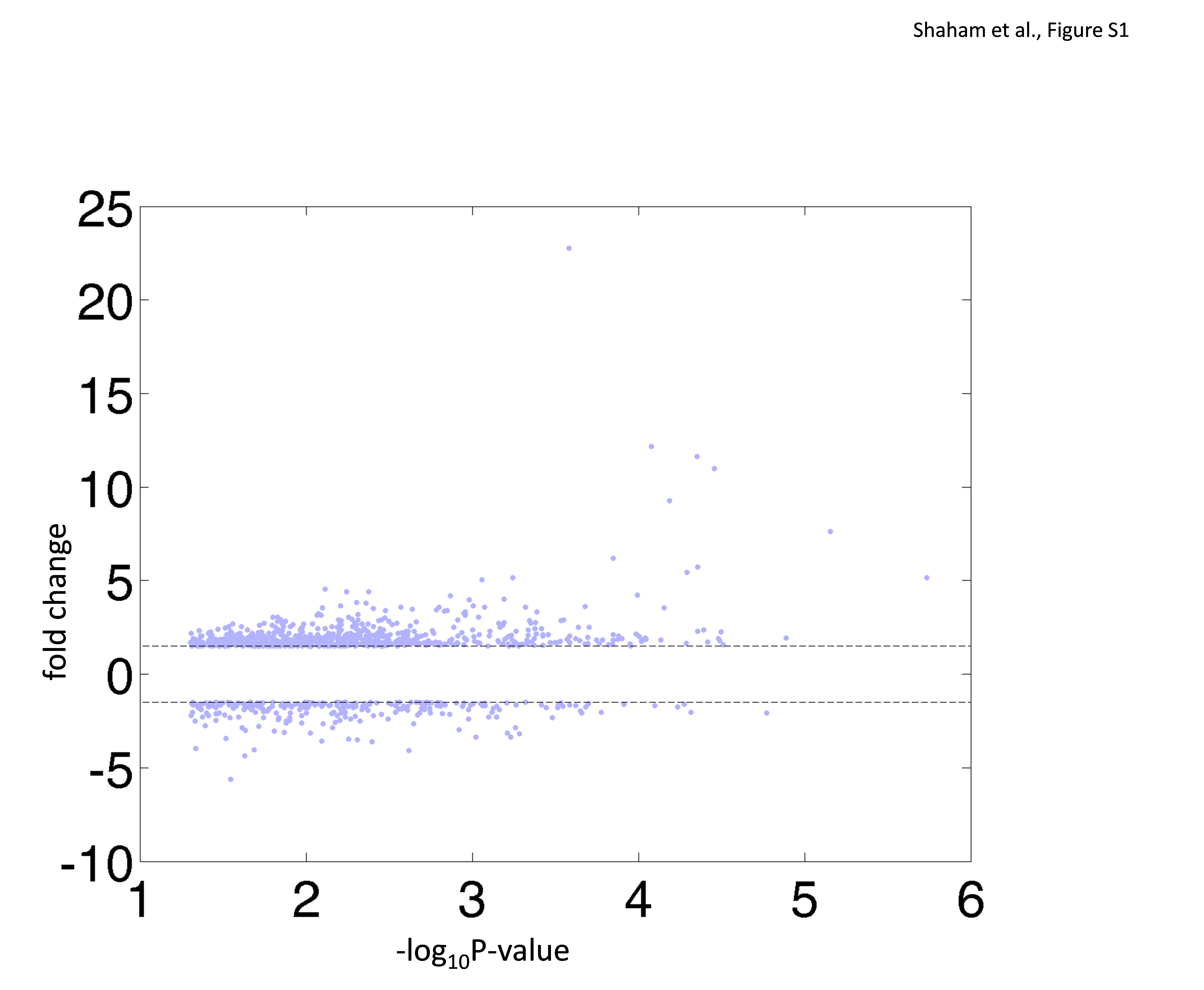

Supplement: Figure S1 — Volcano plot demonstrating differences in gene expression between Pax6loxP/loxP;Mrl10-cre and control E14.5 lens. Each of the 1,013 differentially expressed genes is represented by a single dot. P-values are presented as -log10 values, expression differences are presented as log2 fold-changes; 754 transcripts were up-regulated and 259 were down-regulated in the Pax6-depleted E14.5 lens. (TIF) [file pgen.1003357.s001.tif]

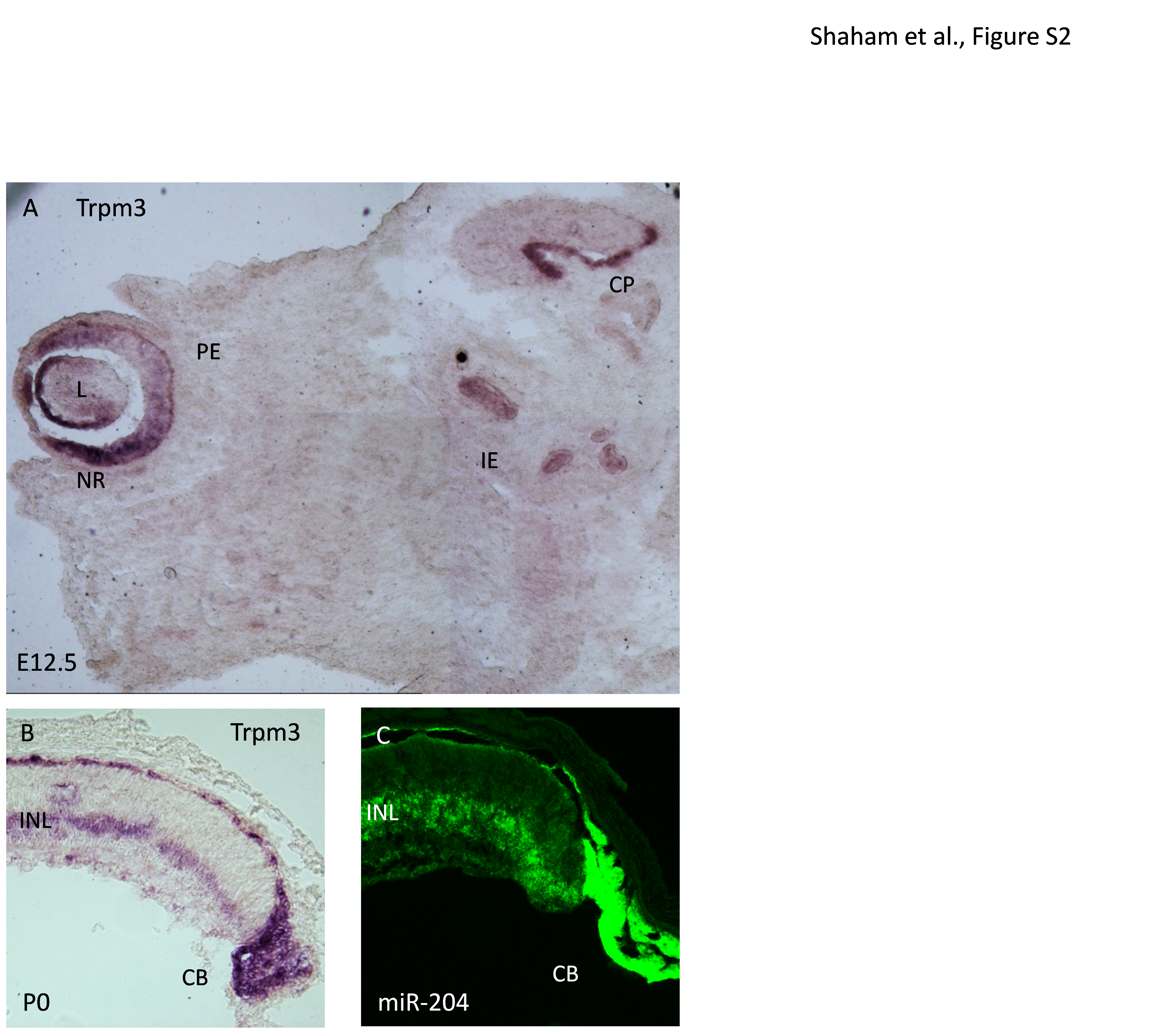

Supplement: Figure S2 — Trpm3 and miR-204 are co-expressed in the embryonic eye. The expression pattern of Trpm3 (A,B) and miR-204 (C) detected by in-situ hybridization on a sagittal section of E12.5 head (A) and P0 optic cup (B,C). Abbreviation: CP, choroid plexus; IE, inner ear; L, lens; PE, pigmented epithelium. (TIF) [file pgen.1003357.s002.tif]

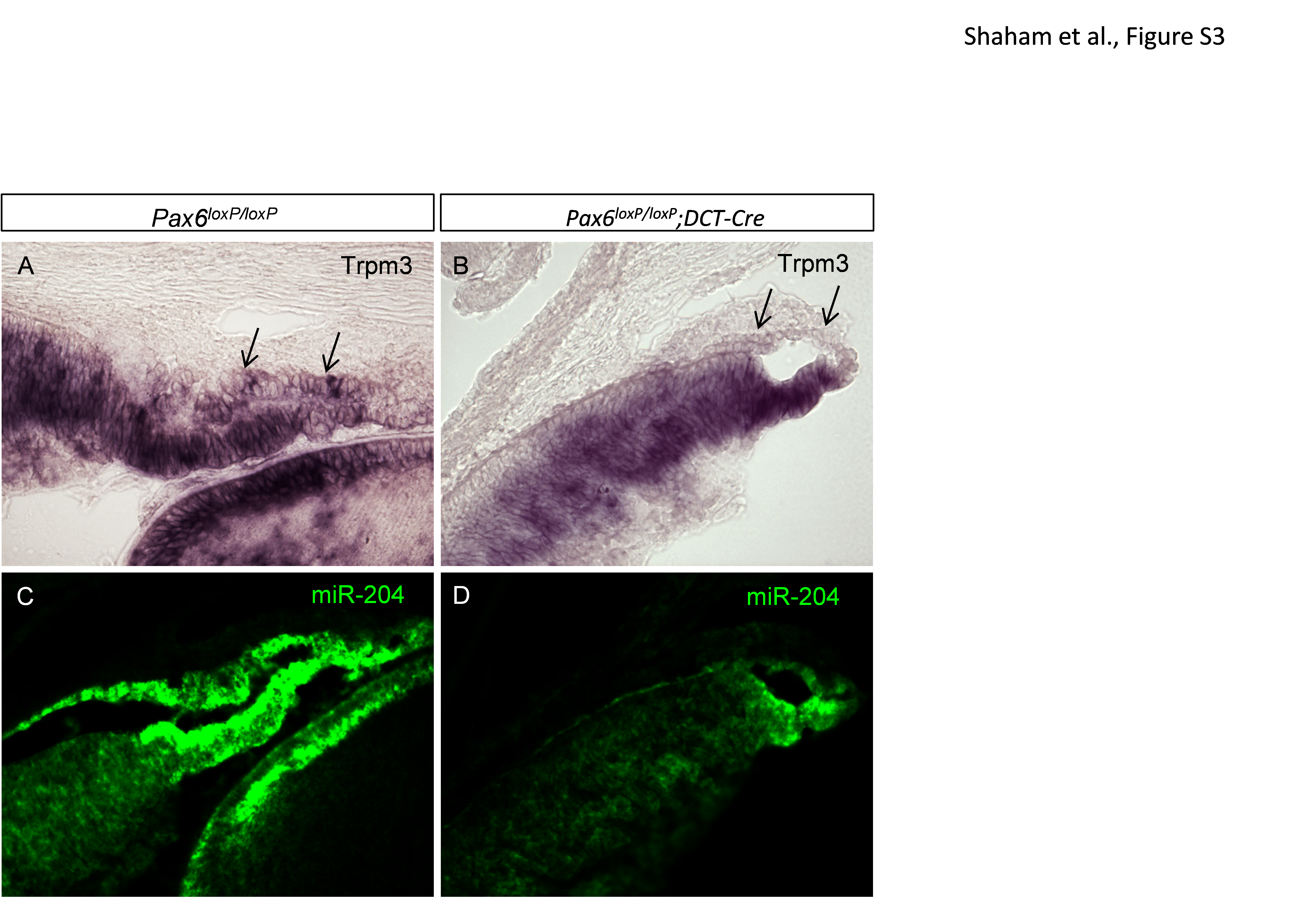

Supplement: Figure S3 — Reduced expression of Trpm3 and miR-204 in progenitors of iris and CB in Pax6 somatic mutants. Trpm3 (A,B) and miR-204 (C,D) in the inner and outer layers of the developing ciliary body and iris of Pax6loxP/loxP control (A,C) but not in the outer layer of the distal optic cup of Pax6loxP/loxP;Dct-Cre mutants (P5). The outer layer of the optic cup is marked with arrows. (TIF) [file pgen.1003357.s003.tif]

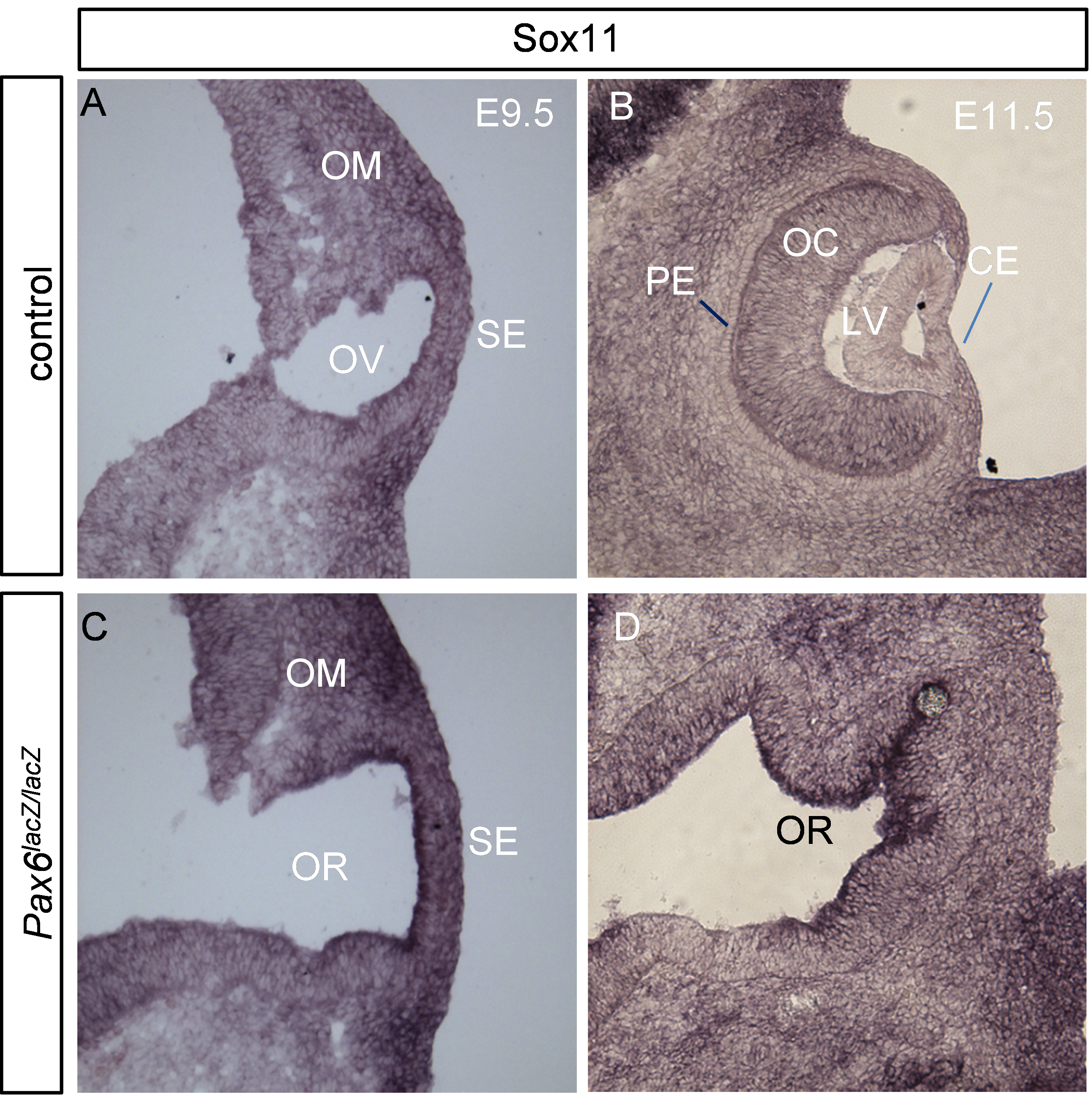

Supplement: Figure S4 — The expression of Sox11 is maintain in Pax6 systemic mutants. Sox11 expression detected by in-situ hybridization in control (A,B) and Pax6lacZ/lacZ (C,D) mutant eye on E9.5 (A,C) and E11.5 (B,D). Abbreviations: CE, corneal epithelium; LV, lens vesicle; OC, optic cup; OM, ocular mesenchyme; OR, ocular rudiment; PE, pigmented epithelium. (TIF) [file pgen.1003357.s004.tif]

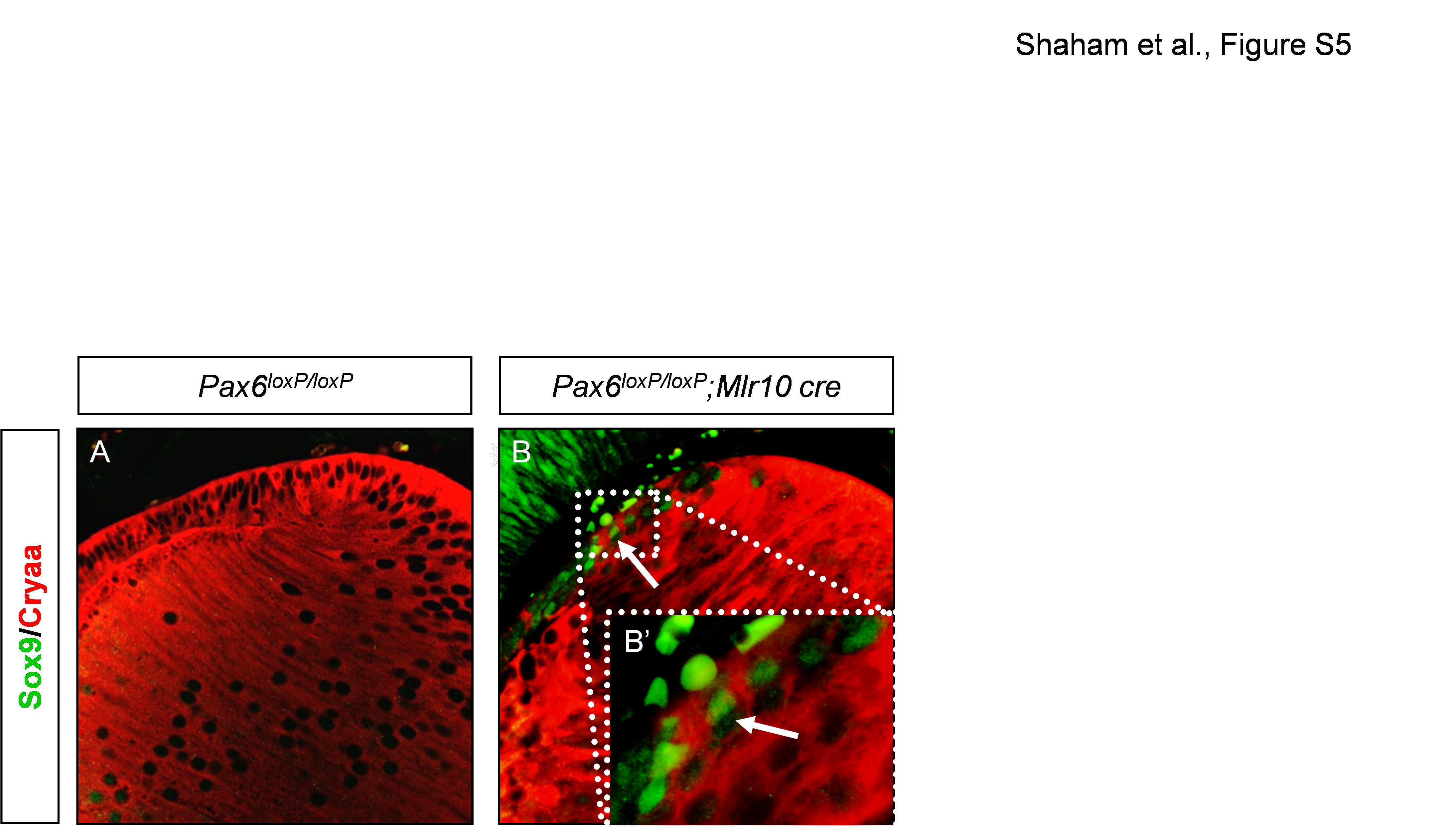

Supplement: Figure S5 — Sox9 is elevated in Pax6 deficient lens. Sox9 detected with antibodies (green) in the Pax6loxP/loxP;Mrl10-Cre (B) but not control Pax6loxP/loxP (A) lens on E14.5. (TIF) [file pgen.1003357.s005.tif]

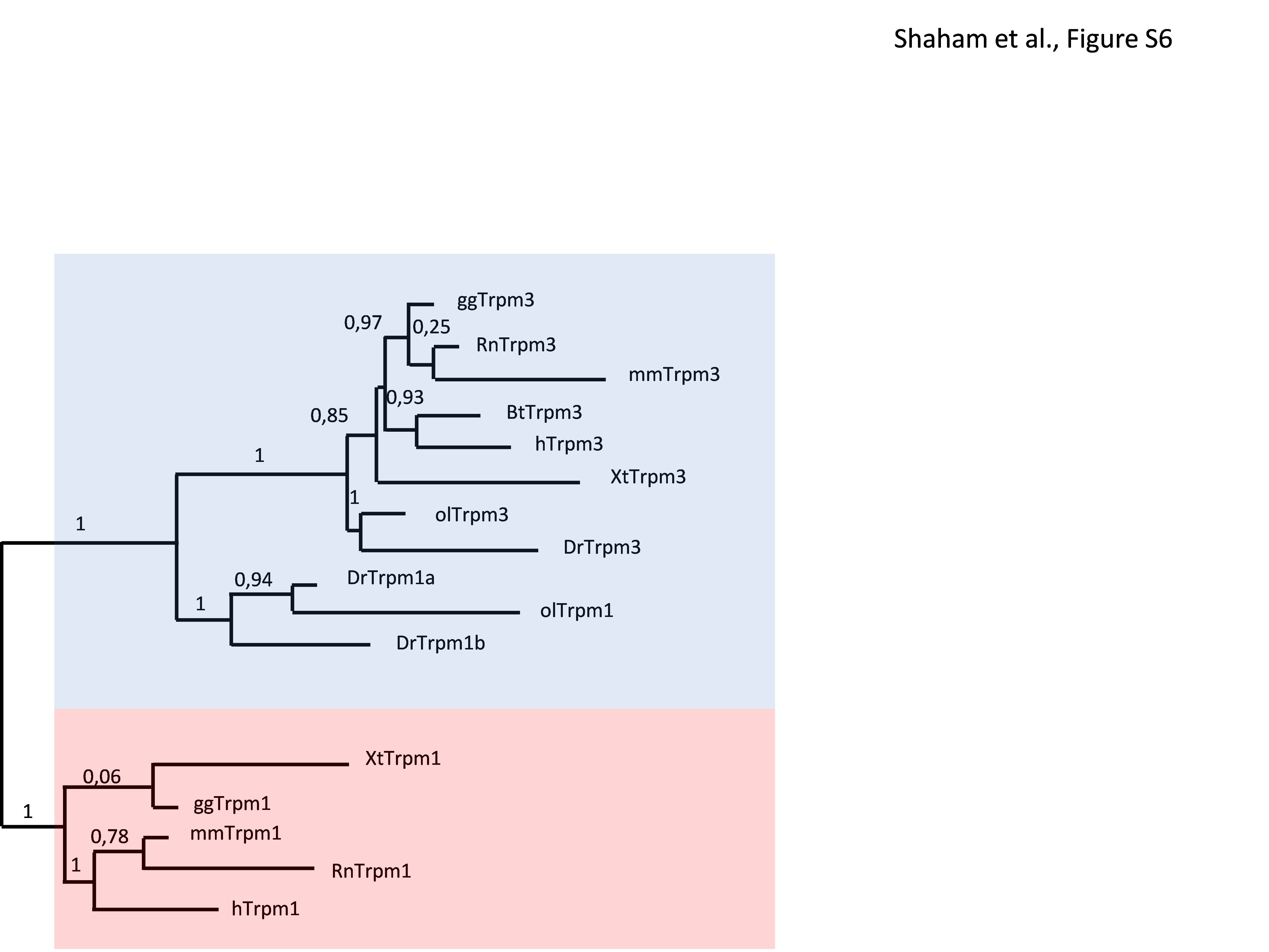

Supplement: Figure S6 — Phylogenetic analysis of the Trpm1 and Trpm3 genes. Phylogenetic tree comparing the amino acid sequences of the Trpm1 and Trpm3 members. Complete or partial protein sequences of all Trpm1 and Trpm3 genes were obtained from the NCBI protein database (Table S4). Genes displaying the greatest sequence similarities cluster together; branch length is proportional to divergence (percentage of amino acid changes). The numbers indicate the bootstrap confidence for each node (n = 1,000). (TIF) [file pgen.1003357.s006.tif]

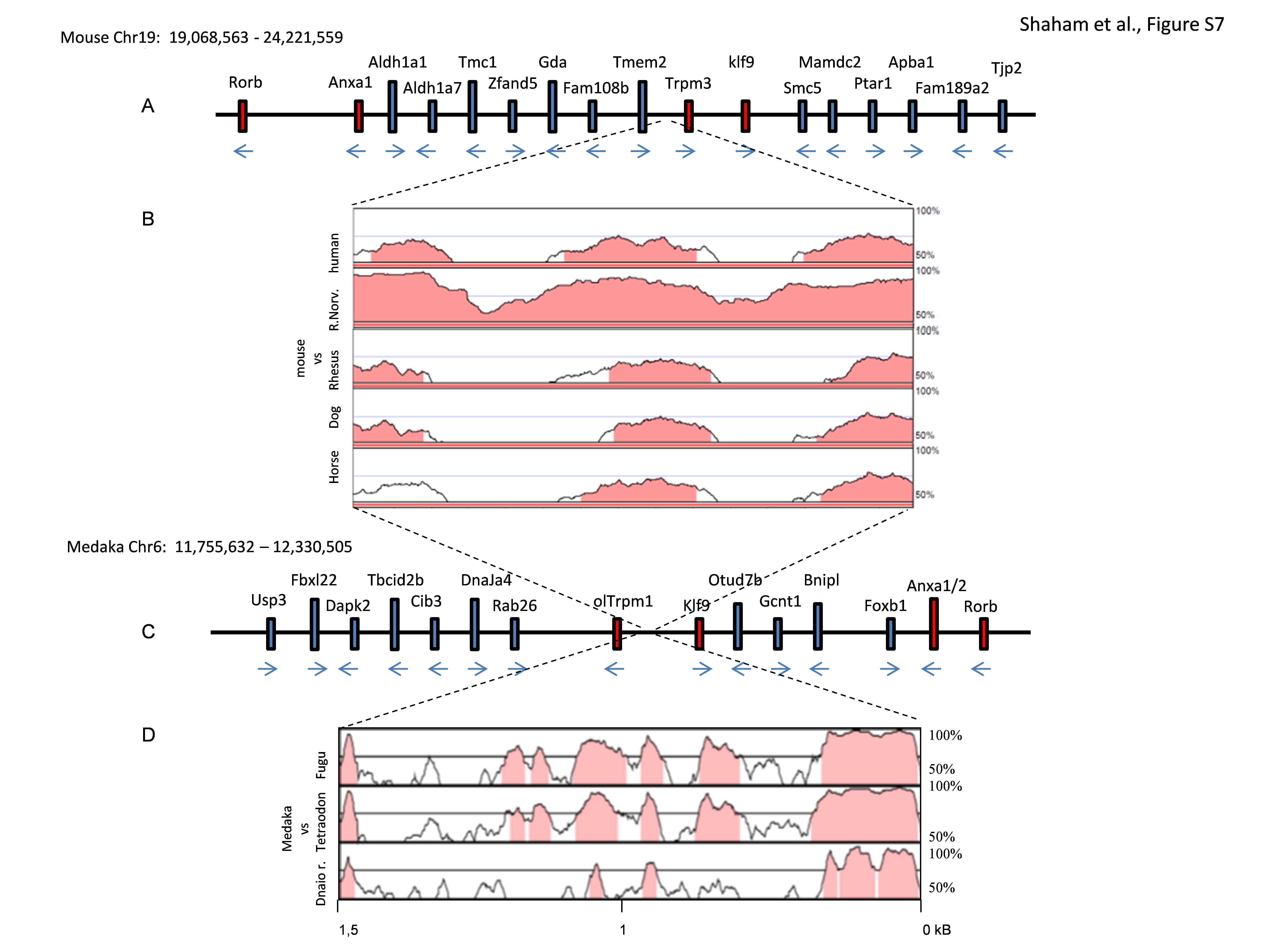

Supplement: Figure S7 — Comparison of the genomic organization between mouse mm-Trpm3 and medaka ol-Trpm3 loci. (A) Schematic representation of the Trpm3 locus in the mouse genome obtained from the UCSC database. (B) Vista comparison of the mouse genomic region, containing the Trpm3.4 box, plotted against those of human, R. norvegicus, rhesus, dog, and horse. (C) Schematic representation of the Trpm1 locus in the medaka genome obtained from the UCSC database. (D) Vista comparison of the medaka genomic region, containing the Trpm3.4 box, plotted against those of fugu, tetraodon and zebrafish. Sequences conserved among the species (60% identity over 100 bp) are indicated in pink. Evolutionarily convergent genes are indicated with red boxes. (TIF) [file pgen.1003357.s007.tif]

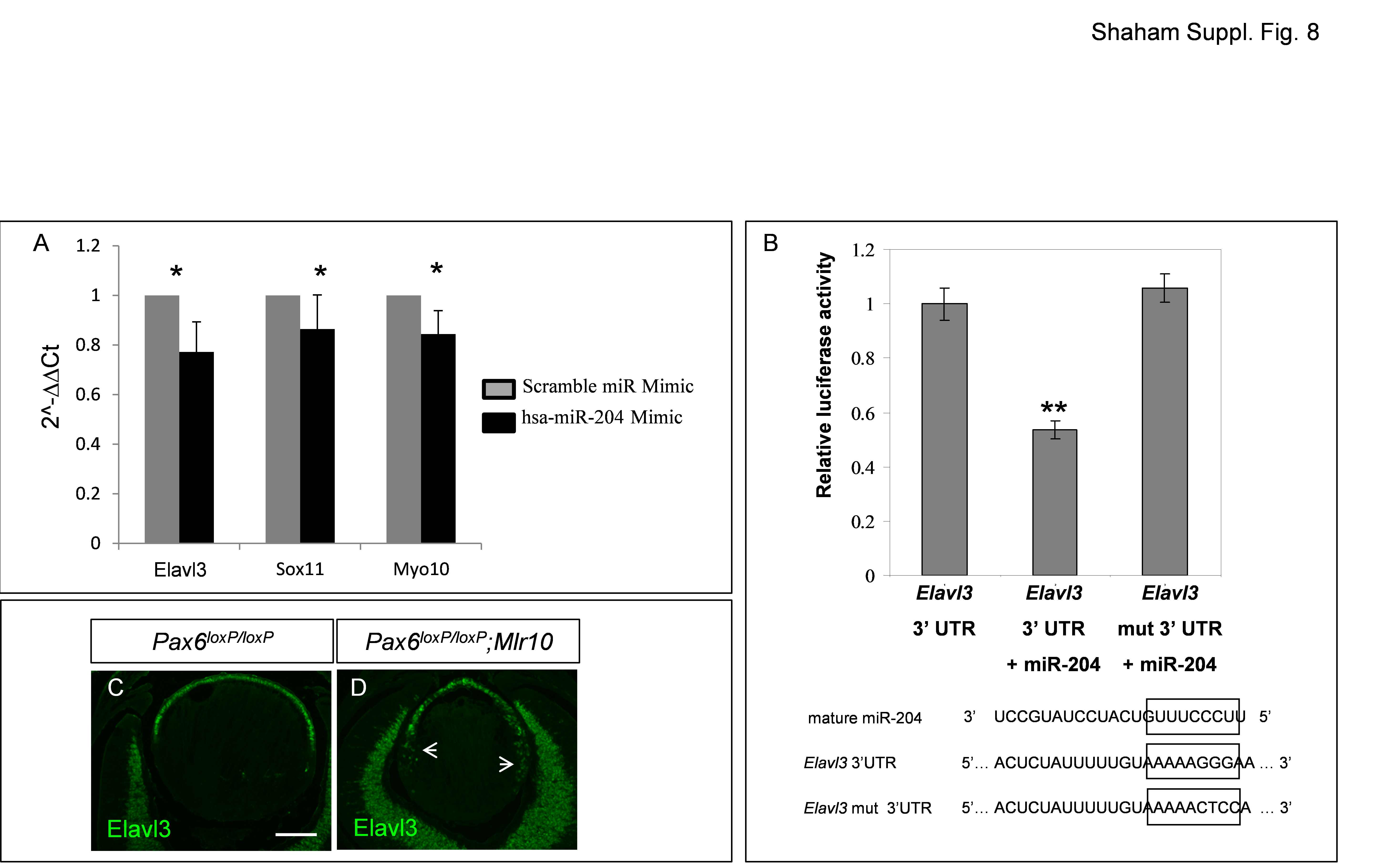

Supplement: Figure S8 — Elavl3 is regulated by miR-204 in Neu-2a cells. (A) Neu-2a cells transfected with miR-204 mimic or scrambled miRNA as a control. Elavl3, Sox11 and Myo10 were significantly down-regulated to 77%, 86% and 84%, respectively. Error bars represent SEM (*P<0.01, n = 7). (B) Relative luciferase luminescence in HEK293T cells transfected with wild-type Elavl3 3′ UTR, or mutated Elavl3 3′ UTR co-transfected with a plasmid containing the pre-miR-204. Error bars represent SEM (P<0.0005; n = 3). Below is the alignment of the miR-204 RNA sequence, wild-type Elavl3 3′ UTR and mutated Elavl3 3′ UTR. (C) Elavl3 immunofluorescence of E14.5 control (D) and Pax6loxP/loxP;Mlr10-cre mutant lens. White arrowheads mark expanded equatorial region. Scale bar = 100 µM. (TIF) [file pgen.1003357.s008.tif]
