# Supplementary material for: Pax6 Regulates Gene Expression in the Vertebrate Lens through miR-204
Source: PLoS Genet. 2013 Mar 14;9(3):e1003357. doi: 10.1371/journal.pgen.1003357 (PMC3597499; doi:10.1371/journal.pgen.1003357)
Supplement: Table S1 — The list of 1,013 differentially expressed genes following Pax6 loss. The transcriptom of the Pax6loxP/loxP;Mlr10-cre E14.5 lenses was compared with that of control Pax6loxP/loxP using the Affymetrix platform. The analysis was performed using Partek Genomics Suite. Differentially expressed genes with P-values lower than 0.05 and with a fold-change cutoff of 1.5 are listed. (DOCX) [file pgen.1003357.s009.docx]

**Table S1.**

| **Gene Symbol** | **Fold Ch(M)** | **P-Val(M)** |
| --- | --- | --- |
| Tph1 | 22.75 | 2.61E-04 |
| Cpxm2 | 12.17 | 8.35E-05 |
| Crabp1 | 11.63 | 4.45E-05 |
| Shisa3 | 10.98 | 3.48E-05 |
| Gjb2 | 9.28 | 6.51E-05 |
| Vstm2b | 7.61 | 7.02E-06 |
| Igfbp3 | 6.20 | 1.42E-04 |
| Cxcl14 | 5.73 | 4.40E-05 |
| Glycam1 | 5.45 | 5.11E-05 |
| Ephx1 | 5.17 | 5.69E-04 |
| Rbp1 | 5.14 | 1.84E-06 |
| Gdf6 | 5.04 | 8.77E-04 |
| Stmn2 | 4.53 | 7.70E-03 |
| Vsx2 | 4.42 | 4.19E-03 |
| Rax | 4.39 | 5.71E-03 |
| Anxa2 | 4.23 | 1.01E-04 |
| Neurod1 | 4.18 | 1.35E-03 |
| Thbs2 | 4.01 | 6.43E-04 |
| Cnksr2 | 3.98 | 1.04E-03 |
| Zic5 | 3.82 | 4.94E-03 |
| Fgf15 | 3.81 | 4.35E-03 |
| Igfbpl1 | 3.67 | 9.90E-04 |
| Zic1 | 3.64 | 6.19E-03 |
| Cdkn1a | 3.63 | 2.10E-04 |
| Mal2 | 3.59 | 4.78E-04 |
| C1ql3 | 3.59 | 8.41E-04 |
| Six6 | 3.58 | 2.68E-03 |
| Hmgcs2 | 3.57 | 1.58E-03 |
| Epha4 | 3.55 | 8.00E-03 |
| Pcp4l1 | 3.54 | 7.02E-05 |
| Nr2e1 | 3.50 | 3.93E-03 |
| Peg10 | 3.46 | 2.29E-03 |
| Gm784 | 3.43 | 1.65E-03 |
| Isl1 | 3.40 | 3.34E-03 |
| Chrna3 | 3.39 | 1.42E-03 |
| Dsp | 3.36 | 1.47E-03 |
| Nefl | 3.34 | 1.10E-03 |
| Nr2f1 | 3.34 | 4.08E-04 |
| Nsg1 | 3.25 | 1.12E-03 |
| Id4 | 3.22 | 1.10E-03 |
| Lrp2 | 3.21 | 8.46E-03 |
| Cdh11 | 3.20 | 4.86E-03 |
| Neurod4 | 3.15 | 8.15E-03 |
| Plxnc1 | 3.14 | 8.55E-03 |
| Tbx2 | 3.14 | 1.10E-03 |
| Chrna4 | 3.05 | 9.14E-04 |
| Mab21l2 | 3.04 | 1.49E-02 |
| Nptx1 | 3.03 | 1.58E-02 |
| Ntng1 | 3.03 | 5.54E-03 |
| Kcnk1 | 3.02 | 3.36E-03 |
| Srgap3 | 2.94 | 5.31E-03 |
| Plagl1 | 2.91 | 4.89E-03 |
| Wfdc1 | 2.91 | 1.22E-03 |
| Gap43 | 2.90 | 6.39E-03 |
| Ppp2r2b | 2.89 | 2.82E-04 |
| Rora | 2.89 | 3.52E-03 |
| Kit | 2.89 | 1.41E-02 |
| Zic2 | 2.88 | 3.64E-03 |
| Bcl11a | 2.88 | 4.53E-04 |
| Scg3 | 2.87 | 2.93E-04 |
| Dapl1 | 2.85 | 6.17E-03 |
| Frk | 2.84 | 1.49E-02 |
| Nefm | 2.83 | 3.17E-03 |
| Arhgap11a | 2.83 | 1.33E-02 |
| Gng3 | 2.81 | 6.28E-03 |
| Tox | 2.80 | 2.17E-03 |
| Foxd1 | 2.75 | 6.43E-04 |
| Rgs3 | 2.75 | 4.16E-04 |
| Reln | 2.74 | 5.08E-03 |
| Tcerg1l | 2.73 | 2.83E-03 |
| Zfp36l1 | 2.73 | 4.43E-04 |
| Dct | 2.72 | 1.79E-02 |
| Necab2 | 2.71 | 6.96E-04 |
| Khdrbs2 | 2.70 | 1.39E-02 |
| Epha5 | 2.69 | 4.23E-03 |
| Sox2 | 2.69 | 4.62E-03 |
| Tiam1 | 2.68 | 1.03E-02 |
| Mybl1 | 2.67 | 9.17E-03 |
| Sh3pxd2b | 2.67 | 2.76E-02 |
| Cldn1 | 2.66 | 4.97E-03 |
| Ina | 2.65 | 3.04E-03 |
| Nov | 2.65 | 1.20E-02 |
| Crmp1 | 2.65 | 6.16E-03 |
| Slc6a15 | 2.63 | 1.60E-02 |
| Tmtc1 | 2.63 | 1.90E-02 |
| Pon3 | 2.62 | 6.54E-04 |
| Lhx2 | 2.59 | 6.30E-03 |
| Lbh | 2.58 | 2.37E-03 |
| Clstn2 | 2.56 | 2.46E-02 |
| Foxn4 | 2.56 | 6.99E-03 |
| Lrrn1 | 2.56 | 3.93E-03 |
| Tek | 2.55 | 2.91E-02 |
| Sstr2 | 2.54 | 1.36E-02 |
| Gpr56 | 2.54 | 1.24E-02 |
| Alox12 | 2.53 | 5.24E-03 |
| Ebf3 | 2.52 | 6.81E-04 |
| Mt1 | 2.52 | 6.49E-03 |
| Lbp | 2.52 | 1.29E-03 |
| Enpp3 | 2.51 | 1.53E-02 |
| St8sia2 | 2.51 | 1.98E-04 |
| Tnfrsf21 | 2.50 | 1.05E-02 |
| Lrrn3 | 2.50 | 2.29E-04 |
| Zfp488 | 2.49 | 1.52E-02 |
| March11 | 2.48 | 4.46E-03 |
| Slc38a8 | 2.47 | 3.07E-02 |
| Uaca | 2.44 | 7.65E-03 |
| Sox9 | 2.44 | 4.20E-04 |
| Syt1 | 2.44 | 5.02E-03 |
| Tcfap2c | 2.43 | 3.81E-04 |
| Cdh20 | 2.43 | 8.50E-03 |
| Fezf2 | 2.42 | 3.58E-03 |
| Tbx3 | 2.42 | 1.07E-02 |
| Ano6 | 2.41 | 3.39E-02 |
| Sncg | 2.37 | 9.96E-04 |
| Itm2a | 2.37 | 4.62E-04 |
| Atoh7 | 2.37 | 4.85E-03 |
| Glrb | 2.37 | 2.26E-02 |
| Snca | 2.37 | 3.43E-03 |
| Pmepa1 | 2.37 | 4.06E-05 |
| OTTMUSG00000010657 | 2.36 | 1.45E-02 |
| Fam46a | 2.36 | 6.15E-03 |
| Cadps | 2.35 | 1.47E-02 |
| Edil3 | 2.35 | 1.08E-02 |
| Crhbp | 2.33 | 1.39E-02 |
| Trib2 | 2.33 | 1.73E-03 |
| Ppfibp2 | 2.33 | 4.38E-02 |
| Dpysl3 | 2.33 | 4.34E-03 |
| Zfp462 | 2.32 | 3.45E-03 |
| Gatm | 2.31 | 4.66E-03 |
| Pter | 2.31 | 2.42E-03 |
| Pde9a | 2.31 | 2.23E-03 |
| Sox11 | 2.30 | 2.83E-02 |
| Scrn1 | 2.30 | 4.38E-05 |
| A730017C20Rik | 2.30 | 6.33E-03 |
| Spock2 | 2.28 | 2.49E-03 |
| Rtn1 | 2.28 | 7.81E-04 |
| ENSMUSG00000050974 | 2.28 | 5.46E-04 |
| Cntn3 | 2.27 | 2.50E-03 |
| Vcan | 2.27 | 1.05E-02 |
| Fstl4 | 2.27 | 1.85E-02 |
| Erbb4 | 2.24 | 1.51E-02 |
| Dtx4 | 2.24 | 3.77E-02 |
| Hspb8 | 2.24 | 4.24E-03 |
| Rab32 | 2.24 | 3.18E-05 |
| Hsd17b11 | 2.22 | 1.94E-02 |
| 6330403K07Rik | 2.22 | 3.70E-03 |
| Ang | 2.21 | 1.16E-03 |
| Tppp3 | 2.21 | 6.32E-03 |
| Cmtm8 | 2.21 | 2.65E-03 |
| Evi1 | 2.21 | 3.81E-02 |
| Ibsp | 2.21 | 1.55E-02 |
| Hist1h1a | 2.20 | 2.79E-03 |
| Ppbp | 2.20 | 4.92E-02 |
| Serinc2 | 2.20 | 1.70E-03 |
| Dhrs3 | 2.20 | 3.71E-03 |
| OTTMUSG00000010671 | 2.20 | 2.50E-02 |
| Nlgn1 | 2.19 | 1.67E-02 |
| 2410066E13Rik | 2.19 | 7.33E-03 |
| Rbms3 | 2.18 | 2.11E-02 |
| Dclk1 | 2.18 | 5.38E-03 |
| Postn | 2.18 | 2.79E-02 |
| Pcolce | 2.17 | 1.27E-02 |
| Frem1 | 2.17 | 3.86E-02 |
| Grb14 | 2.16 | 6.87E-03 |
| Foxi3 | 2.16 | 3.48E-03 |
| Fgf9 | 2.15 | 5.65E-03 |
| Gcnt1 | 2.15 | 1.08E-02 |
| Dcx | 2.15 | 4.73E-03 |
| Rbms1 | 2.14 | 1.03E-04 |
| 1810041L15Rik | 2.14 | 4.12E-04 |
| Rgs5 | 2.13 | 3.11E-03 |
| Galntl4 | 2.13 | 5.36E-03 |
| St8sia4 | 2.13 | 7.05E-03 |
| Trim47 | 2.13 | 9.09E-04 |
| Otx2 | 2.13 | 1.90E-03 |
| Plcd1 | 2.13 | 3.59E-03 |
| Rdh10 | 2.12 | 9.42E-03 |
| Adamts1 | 2.12 | 1.41E-02 |
| Sept5 | 2.12 | 1.42E-04 |
| Rbpms | 2.11 | 3.76E-04 |
| BC048355 | 2.11 | 5.26E-03 |
| Fbn1 | 2.11 | 3.01E-02 |
| Fbn2 | 2.11 | 2.60E-02 |
| St6gal1 | 2.11 | 4.16E-03 |
| Robo2 | 2.11 | 1.93E-02 |
| Hmcn1 | 2.11 | 3.45E-03 |
| Grit | 2.10 | 8.53E-03 |
| Ptk7 | 2.10 | 4.90E-03 |
| Efhd1 | 2.10 | 2.09E-02 |
| Tmsb4x | 2.10 | 2.11E-02 |
| Ebf1 | 2.10 | 2.96E-02 |
| Pcbp3 | 2.10 | 3.48E-04 |
| Penk1 | 2.09 | 5.80E-04 |
| Rbpms2 | 2.09 | 6.36E-03 |
| Sox6 | 2.09 | 2.60E-03 |
| Ascl1 | 2.09 | 4.61E-04 |
| 4930523C07Rik | 2.09 | 2.45E-03 |
| Pak3 | 2.08 | 3.80E-03 |
| Nkain3 | 2.08 | 3.95E-03 |
| Hhip | 2.07 | 3.70E-02 |
| Tubb3 | 2.07 | 4.15E-03 |
| Cd97 | 2.07 | 5.81E-03 |
| Csrnp3 | 2.07 | 1.08E-02 |
| Ubash3b | 2.07 | 4.76E-03 |
| Laptm5 | 2.06 | 1.15E-02 |
| Adam22 | 2.06 | 1.84E-02 |
| Fzd5 | 2.05 | 7.87E-04 |
| Pdgfc | 2.05 | 5.47E-03 |
| Glul | 2.05 | 3.31E-03 |
| Neto2 | 2.05 | 1.36E-02 |
| Camk2n1 | 2.05 | 2.15E-03 |
| Nrip1 | 2.05 | 4.23E-02 |
| 2610301F02Rik | 2.05 | 1.14E-02 |
| Lrp5 | 2.04 | 9.45E-03 |
| Fbxo44 | 2.04 | 3.77E-04 |
| Igfbp2 | 2.04 | 5.99E-04 |
| Cdo1 | 2.04 | 1.32E-04 |
| Elavl3 | 2.04 | 4.86E-03 |
| Apba2 | 2.04 | 1.78E-02 |
| 1700029I01Rik | 2.04 | 4.37E-03 |
| Slc1a3 | 2.03 | 4.31E-02 |
| Rhoj | 2.03 | 1.22E-03 |
| Gpc2 | 2.03 | 2.02E-03 |
| Neurog2 | 2.03 | 2.88E-02 |
| Racgap1 | 2.03 | 2.60E-03 |
| 1700006J14Rik | 2.03 | 2.60E-04 |
| A730049H05Rik | 2.03 | 7.11E-03 |
| Bai3 | 2.02 | 7.53E-03 |
| Sepp1 | 2.02 | 9.89E-05 |
| Lpar4 | 2.02 | 2.14E-02 |
| OTTMUSG00000011097 | 2.01 | 1.57E-02 |
| Syt9 | 2.00 | 2.49E-02 |
| Prdm1 | 2.00 | 7.65E-03 |
| C3ar1 | 2.00 | 9.73E-04 |
| Cdc14a | 2.00 | 2.70E-02 |
| Frem2 | 1.99 | 2.19E-02 |
| Pfkp | 1.99 | 2.51E-03 |
| Mgp | 1.99 | 1.16E-02 |
| Sgce | 1.99 | 3.46E-03 |
| Txnip | 1.99 | 1.01E-02 |
| Ndp | 1.99 | 6.10E-04 |
| Atp1b2 | 1.98 | 1.32E-02 |
| Rtn4rl1 | 1.98 | 3.75E-02 |
| Tbx5 | 1.98 | 3.77E-03 |
| Camta1 | 1.98 | 6.81E-03 |
| Has2 | 1.97 | 1.92E-02 |
| Trim59 | 1.97 | 2.02E-04 |
| Elovl2 | 1.97 | 1.97E-02 |
| Sgk1 | 1.97 | 2.11E-03 |
| LOC665622 | 1.97 | 2.42E-02 |
| Vash1 | 1.96 | 5.19E-03 |
| Adamts9 | 1.96 | 1.64E-02 |
| Pou3f3 | 1.95 | 6.29E-03 |
| Arhgef4 | 1.94 | 2.25E-02 |
| Fat3 | 1.94 | 1.99E-02 |
| Ccdc60 | 1.94 | 4.70E-03 |
| Lxn | 1.94 | 3.67E-03 |
| BC005764 | 1.94 | 1.11E-03 |
| Rnpep | 1.94 | 9.10E-05 |
| BC056474 | 1.94 | 1.16E-02 |
| Ptprz1 | 1.93 | 1.04E-02 |
| Tec | 1.93 | 9.52E-03 |
| Zfp57 | 1.93 | 1.85E-02 |
| LOC100041230 | 1.93 | 8.17E-03 |
| Lmod1 | 1.93 | 1.29E-05 |
| 2310014D11Rik | 1.92 | 5.60E-03 |
| Serpine2 | 1.92 | 8.98E-03 |
| Rbp2 | 1.92 | 7.88E-03 |
| Nnat | 1.92 | 3.02E-02 |
| Prokr1 | 1.92 | 2.75E-02 |
| Stard5 | 1.91 | 4.77E-02 |
| Plxnb1 | 1.91 | 9.57E-03 |
| Rassf2 | 1.91 | 4.22E-04 |
| Tmeff1 | 1.91 | 2.59E-03 |
| Khdrbs3 | 1.91 | 3.30E-05 |
| Ly86 | 1.91 | 1.10E-02 |
| Atp2a1 | 1.91 | 2.54E-02 |
| Myo10 | 1.91 | 2.07E-02 |
| 4631426J05Rik | 1.90 | 6.02E-03 |
| Unc5b | 1.90 | 4.12E-03 |
| Mmp14 | 1.90 | 2.97E-02 |
| Tspan7 | 1.90 | 2.39E-04 |
| Ccne1 | 1.90 | 1.25E-04 |
| Chrnb4 | 1.90 | 1.71E-02 |
| 6820445E23Rik | 1.90 | 2.26E-02 |
| Gucy1a2 | 1.89 | 3.20E-03 |
| Ldb2 | 1.89 | 1.10E-03 |
| Sept3 | 1.89 | 4.36E-03 |
| Mcam | 1.88 | 2.16E-02 |
| Lypd6 | 1.88 | 4.96E-03 |
| Zic3 | 1.88 | 3.27E-02 |
| Nppb | 1.88 | 2.62E-02 |
| Rgs2 | 1.88 | 2.64E-03 |
| Pdpn | 1.88 | 7.39E-03 |
| 1110067D22Rik | 1.88 | 1.33E-03 |
| Ube2d1 | 1.87 | 8.97E-05 |
| Fgf14 | 1.87 | 5.96E-03 |
| Prim1 | 1.87 | 2.44E-03 |
| Nfia | 1.87 | 1.09E-03 |
| Cdh8 | 1.87 | 5.89E-03 |
| 6330407J23Rik | 1.87 | 2.60E-02 |
| Nr2f2 | 1.87 | 9.52E-03 |
| Scoc | 1.87 | 6.35E-04 |
| Pde1b | 1.86 | 7.02E-03 |
| Cntn2 | 1.86 | 1.72E-02 |
| Cdon | 1.86 | 3.52E-02 |
| Olfm1 | 1.85 | 2.22E-03 |
| Gal | 1.85 | 8.94E-03 |
| Cugbp2 | 1.85 | 3.16E-02 |
| C1ql1 | 1.85 | 2.70E-04 |
| Obfc2a | 1.85 | 3.50E-02 |
| Hif3a | 1.85 | 1.33E-04 |
| Pmm1 | 1.85 | 8.57E-03 |
| Hey2 | 1.85 | 2.77E-03 |
| Cdk6 | 1.85 | 4.67E-02 |
| 2610305D13Rik | 1.85 | 8.41E-03 |
| Kcnj13 | 1.85 | 1.03E-02 |
| Dclk2 | 1.84 | 2.61E-02 |
| 2810030E01Rik | 1.84 | 9.10E-03 |
| B2m | 1.84 | 1.79E-04 |
| Lass6 | 1.84 | 1.03E-02 |
| Sh3pxd2a | 1.84 | 2.52E-02 |
| Onecut1 | 1.84 | 2.91E-02 |
| Dchs1 | 1.84 | 1.38E-02 |
| Igfbp4 | 1.83 | 5.74E-04 |
| Madd | 1.83 | 3.19E-03 |
| Cacnb3 | 1.83 | 9.42E-05 |
| Pros1 | 1.83 | 4.38E-02 |
| Slc4a1 | 1.83 | 3.25E-02 |
| Lphn3 | 1.83 | 2.54E-02 |
| Islr2 | 1.83 | 2.25E-02 |
| Arntl | 1.83 | 4.70E-04 |
| Tle1 | 1.82 | 2.18E-03 |
| 2610039C10Rik | 1.82 | 1.08E-03 |
| Meis1 | 1.82 | 1.87E-02 |
| Tnrc4 | 1.82 | 1.23E-02 |
| Mpp1 | 1.82 | 1.41E-04 |
| Cd9 | 1.82 | 2.80E-02 |
| Mfng | 1.82 | 7.34E-05 |
| Dock11 | 1.82 | 3.56E-02 |
| S100a10 | 1.82 | 3.26E-03 |
| Mbc2 | 1.82 | 5.40E-03 |
| LOC100044854 | 1.82 | 8.88E-03 |
| Nphs1 | 1.81 | 1.54E-02 |
| Sorcs1 | 1.81 | 1.23E-02 |
| Cacng4 | 1.81 | 2.40E-03 |
| Masp2 | 1.81 | 1.64E-04 |
| Fabp5 | 1.81 | 8.71E-03 |
| Nhlh2 | 1.81 | 1.25E-02 |
| Nsbp1 | 1.80 | 1.58E-02 |
| 3632451O06Rik | 1.80 | 7.84E-03 |
| Mark1 | 1.80 | 4.46E-03 |
| Slc43a1 | 1.80 | 1.84E-03 |
| Foxp2 | 1.80 | 7.73E-03 |
| Parp6 | 1.80 | 6.99E-04 |
| Serpind1 | 1.80 | 2.30E-03 |
| Axl | 1.80 | 1.11E-02 |
| Met | 1.80 | 2.89E-02 |
| Rasgef1b | 1.80 | 5.86E-03 |
| Tmem100 | 1.80 | 1.51E-02 |
| Nrg1 | 1.80 | 9.09E-03 |
| Dach1 | 1.79 | 6.44E-03 |
| Gpm6a | 1.79 | 1.53E-02 |
| Hey1 | 1.79 | 4.18E-03 |
| Rftn1 | 1.79 | 3.85E-02 |
| Mex3b | 1.79 | 1.50E-02 |
| Enc1 | 1.78 | 1.36E-03 |
| Hpgd | 1.78 | 2.25E-04 |
| Ntrk2 | 1.78 | 2.69E-03 |
| Pdlim1 | 1.78 | 3.24E-05 |
| 1500001A10Rik | 1.78 | 1.15E-02 |
| Arhgap4 | 1.78 | 1.22E-02 |
| Crot | 1.78 | 9.65E-03 |
| Upp1 | 1.78 | 1.76E-02 |
| 2410129H14Rik | 1.78 | 3.58E-02 |
| Pitx1 | 1.77 | 1.86E-02 |
| Gria4 | 1.77 | 3.03E-02 |
| Mpeg1 | 1.77 | 1.05E-02 |
| Vill | 1.77 | 2.04E-02 |
| Atoh8 | 1.77 | 9.46E-03 |
| Gfra1 | 1.76 | 4.78E-02 |
| Gadd45b | 1.76 | 2.86E-03 |
| Dlx1 | 1.76 | 2.01E-02 |
| Epas1 | 1.76 | 7.27E-03 |
| Ntng2 | 1.76 | 2.36E-03 |
| St3gal1 | 1.76 | 1.15E-02 |
| Pdgfd | 1.76 | 2.82E-02 |
| Mtus1 | 1.76 | 2.12E-02 |
| Sdpr | 1.76 | 4.66E-02 |
| Pou6f2 | 1.76 | 2.24E-02 |
| Zdbf2 | 1.76 | 1.47E-02 |
| Ptrf | 1.76 | 7.05E-03 |
| OTTMUSG00000015750 | 1.76 | 9.61E-03 |
| Mdk | 1.75 | 2.94E-04 |
| Padi4 | 1.75 | 1.87E-02 |
| Rrm2 | 1.75 | 3.65E-03 |
| Gucy1a3 | 1.75 | 4.47E-02 |
| Cdc42ep3 | 1.75 | 9.65E-04 |
| Mapk8ip2 | 1.75 | 1.51E-02 |
| Vangl1 | 1.74 | 9.60E-05 |
| Scd1 | 1.74 | 6.08E-03 |
| Dner | 1.74 | 7.66E-03 |
| Sema7a | 1.74 | 2.42E-03 |
| Tshz3 | 1.74 | 1.87E-02 |
| Optc | 1.74 | 2.40E-02 |
| Insm1 | 1.74 | 3.91E-03 |
| Lum | 1.73 | 4.37E-02 |
| Npr1 | 1.73 | 4.52E-03 |
| Tagln3 | 1.73 | 3.24E-03 |
| LOC676910 | 1.73 | 3.84E-02 |
| Mamld1 | 1.73 | 1.36E-03 |
| Thsd7a | 1.73 | 2.33E-02 |
| Enah | 1.72 | 3.84E-05 |
| Ppic | 1.72 | 4.39E-04 |
| Tm6sf1 | 1.72 | 2.89E-03 |
| 1200009O22Rik | 1.72 | 2.31E-03 |
| Myt1 | 1.72 | 3.44E-02 |
| Ldhb | 1.72 | 2.02E-02 |
| Arsb | 1.72 | 6.93E-04 |
| Nsg2 | 1.72 | 5.39E-03 |
| Il17rd | 1.72 | 1.20E-02 |
| Brca1 | 1.71 | 2.12E-02 |
| Trp53i11 | 1.71 | 1.66E-03 |
| Cox6a2 | 1.71 | 1.30E-02 |
| Foxp4 | 1.71 | 5.53E-04 |
| E2f7 | 1.71 | 1.46E-02 |
| Asf1b | 1.71 | 5.21E-03 |
| Rbbp8 | 1.71 | 5.35E-03 |
| Tbc1d8b | 1.71 | 3.46E-02 |
| Elovl5 | 1.71 | 4.04E-03 |
| Prrx1 | 1.71 | 4.91E-02 |
| Cks2 | 1.71 | 3.45E-02 |
| Tet1 | 1.71 | 5.42E-03 |
| Acta2 | 1.70 | 4.97E-02 |
| Nudt4 | 1.70 | 5.01E-03 |
| Plce1 | 1.70 | 3.76E-02 |
| Pop4 | 1.70 | 3.58E-02 |
| Runx1 | 1.70 | 6.05E-03 |
| Nusap1 | 1.70 | 6.23E-03 |
| Prom1 | 1.70 | 2.03E-02 |
| 1810011O10Rik | 1.70 | 3.33E-02 |
| D930015E06Rik | 1.70 | 9.25E-03 |
| Vat1l | 1.70 | 4.16E-02 |
| Dpysl5 | 1.69 | 1.14E-02 |
| Lphn2 | 1.69 | 1.94E-03 |
| Snap25 | 1.69 | 3.28E-03 |
| Flt1 | 1.69 | 4.62E-02 |
| Cdc25b | 1.69 | 1.57E-03 |
| Tmem132e | 1.69 | 5.07E-03 |
| Snai2 | 1.69 | 4.52E-03 |
| Slc1a2 | 1.69 | 4.80E-02 |
| Agrn | 1.69 | 2.33E-02 |
| 2010011I20Rik | 1.69 | 2.58E-04 |
| Gpr149 | 1.69 | 1.27E-02 |
| Hist2h2ac | 1.69 | 2.50E-02 |
| Lgi1 | 1.68 | 3.06E-02 |
| Alas2 | 1.68 | 2.59E-03 |
| 2610020H08Rik | 1.68 | 2.13E-02 |
| Tle4 | 1.68 | 1.29E-02 |
| Wdhd1 | 1.68 | 3.01E-02 |
| Klf3 | 1.68 | 3.09E-04 |
| Plscr1 | 1.68 | 2.61E-03 |
| Mertk | 1.68 | 1.39E-02 |
| Dll1 | 1.68 | 2.12E-02 |
| Mmp15 | 1.68 | 2.30E-03 |
| Ntn1 | 1.68 | 3.45E-03 |
| Cenpv | 1.68 | 3.86E-02 |
| Epcam | 1.68 | 1.04E-02 |
| Plcl1 | 1.68 | 3.37E-02 |
| ENSMUSG00000038594 | 1.68 | 7.97E-03 |
| Jup | 1.67 | 3.19E-04 |
| Usp1 | 1.67 | 9.28E-04 |
| Zfp503 | 1.67 | 1.23E-03 |
| Myb | 1.67 | 5.36E-03 |
| Arhgap10 | 1.67 | 3.39E-04 |
| Mapt | 1.67 | 1.88E-03 |
| Mkrn1 | 1.67 | 4.40E-03 |
| Ssh2 | 1.67 | 4.20E-02 |
| Rad51 | 1.67 | 2.98E-02 |
| Samd14 | 1.67 | 1.09E-02 |
| Serpinb6a | 1.67 | 2.61E-02 |
| Tbx20 | 1.67 | 1.51E-02 |
| Fam171b | 1.67 | 9.73E-03 |
| Tmprss11d | 1.66 | 2.50E-02 |
| Ucp2 | 1.66 | 2.07E-03 |
| Heyl | 1.66 | 1.22E-03 |
| Six6os1 | 1.66 | 3.07E-02 |
| Tank | 1.66 | 2.67E-03 |
| Aldh5a1 | 1.66 | 6.37E-03 |
| Tspan13 | 1.66 | 4.68E-04 |
| Gm447 | 1.66 | 4.15E-02 |
| Ttl | 1.66 | 1.85E-02 |
| Slc8a3 | 1.66 | 3.02E-02 |
| Slc44a5 | 1.66 | 5.15E-05 |
| 4930452B06Rik | 1.66 | 2.10E-02 |
| Mdc1 | 1.65 | 2.40E-02 |
| Ephb2 | 1.65 | 1.20E-02 |
| Gng12 | 1.65 | 5.36E-03 |
| Man1a | 1.65 | 1.79E-02 |
| Eef2k | 1.65 | 4.61E-03 |
| Ppap2b | 1.65 | 2.96E-02 |
| Pik3r1 | 1.65 | 1.70E-02 |
| Ddr2 | 1.65 | 4.61E-02 |
| E2f8 | 1.65 | 1.91E-02 |
| Gdf1 | 1.65 | 5.06E-03 |
| Pip4k2a | 1.65 | 2.82E-03 |
| Plxnd1 | 1.64 | 1.73E-02 |
| Rgs12 | 1.64 | 3.48E-02 |
| Actn1 | 1.64 | 3.10E-03 |
| Ntan1 | 1.64 | 1.74E-03 |
| Emcn | 1.64 | 3.53E-03 |
| Dll4 | 1.64 | 1.38E-02 |
| Abca1 | 1.64 | 4.24E-02 |
| Prkch | 1.64 | 1.70E-02 |
| Gypa | 1.64 | 8.96E-04 |
| Gfra2 | 1.64 | 4.66E-02 |
| Clspn | 1.64 | 2.49E-02 |
| E130309F12Rik | 1.64 | 2.43E-03 |
| Lix1l | 1.64 | 2.02E-04 |
| Nkain4 | 1.64 | 8.57E-03 |
| Fxyd6 | 1.64 | 2.12E-03 |
| Ifi30 | 1.64 | 3.64E-02 |
| Itpripl2 | 1.64 | 2.60E-03 |
| Kpna2 | 1.63 | 1.72E-04 |
| Frmpd1 | 1.63 | 5.03E-04 |
| Calcrl | 1.63 | 2.82E-02 |
| Crip2 | 1.63 | 3.30E-02 |
| Ssbp4 | 1.63 | 2.20E-03 |
| Pf4 | 1.63 | 1.58E-02 |
| Chd3 | 1.63 | 3.06E-02 |
| Tns1 | 1.63 | 2.27E-02 |
| Spata13 | 1.63 | 4.33E-02 |
| Car2 | 1.62 | 7.11E-03 |
| Ptms | 1.62 | 4.75E-04 |
| Slc24a3 | 1.62 | 2.88E-02 |
| Atp10d | 1.62 | 4.78E-02 |
| Cadm3 | 1.62 | 4.88E-03 |
| Fnbp1 | 1.62 | 3.29E-02 |
| Brca2 | 1.62 | 7.79E-03 |
| Ctss | 1.62 | 4.10E-03 |
| Scarb2 | 1.62 | 1.71E-02 |
| Slc7a7 | 1.62 | 5.03E-03 |
| Tes | 1.62 | 1.05E-02 |
| Hes6 | 1.62 | 4.53E-03 |
| Hist1h1b | 1.62 | 5.90E-03 |
| Exo1 | 1.62 | 2.53E-02 |
| Rgs8 | 1.62 | 6.54E-04 |
| Eng | 1.62 | 3.66E-03 |
| OTTMUSG00000005773 | 1.62 | 4.53E-02 |
| EG667519 | 1.62 | 1.46E-03 |
| Clec2d | 1.62 | 4.67E-03 |
| Ifitm3 | 1.61 | 1.12E-04 |
| Jak2 | 1.61 | 4.56E-02 |
| Adarb1 | 1.61 | 2.99E-03 |
| 2810417H13Rik | 1.61 | 1.97E-02 |
| Elavl4 | 1.61 | 1.80E-02 |
| 2810474O19Rik | 1.61 | 3.41E-03 |
| 4732418C07Rik | 1.61 | 4.71E-02 |
| Zfp583 | 1.61 | 1.05E-02 |
| Sbk1 | 1.61 | 3.36E-02 |
| Gdpd5 | 1.60 | 7.60E-03 |
| Ckb | 1.60 | 5.58E-03 |
| Sulf2 | 1.60 | 1.65E-02 |
| Tmem106a | 1.60 | 4.14E-03 |
| Mtap1b | 1.60 | 6.27E-03 |
| Gpc3 | 1.60 | 5.79E-03 |
| Nrm | 1.60 | 3.18E-02 |
| Rimbp2 | 1.60 | 1.23E-02 |
| Abca3 | 1.60 | 6.06E-04 |
| Eya2 | 1.60 | 1.43E-04 |
| Mdga2 | 1.60 | 5.32E-04 |
| EG668588 | 1.60 | 8.40E-03 |
| Sdc3 | 1.60 | 1.65E-02 |
| Ccdc8 | 1.60 | 1.13E-02 |
| P4ha3 | 1.60 | 5.38E-03 |
| Peg12 | 1.60 | 4.86E-03 |
| Zmiz1 | 1.60 | 2.10E-04 |
| S100a11 | 1.59 | 3.04E-02 |
| Bdh2 | 1.59 | 1.59E-03 |
| Pard6g | 1.59 | 6.53E-03 |
| Lgr5 | 1.59 | 1.90E-02 |
| Raph1 | 1.59 | 1.84E-03 |
| Slc4a5 | 1.59 | 2.47E-02 |
| ENSMUSG00000051554 | 1.59 | 3.93E-02 |
| Pkd1l3 | 1.59 | 1.74E-02 |
| Nedd9 | 1.58 | 1.98E-02 |
| Tll1 | 1.58 | 1.93E-02 |
| Gsta4 | 1.58 | 2.85E-03 |
| Hivep2 | 1.58 | 2.48E-02 |
| Stmn1 | 1.58 | 4.27E-03 |
| Cpne8 | 1.58 | 4.13E-02 |
| Arhgdib | 1.58 | 8.18E-03 |
| Syp | 1.58 | 1.96E-02 |
| B230399E16Rik | 1.58 | 2.98E-02 |
| 6720463M24Rik | 1.58 | 7.52E-03 |
| Fam101b | 1.58 | 3.77E-02 |
| Ctns | 1.57 | 3.08E-05 |
| Pde5a | 1.57 | 5.95E-03 |
| AK129341 | 1.57 | 5.52E-03 |
| Zcwpw1 | 1.57 | 4.70E-02 |
| Vps37b | 1.57 | 4.06E-02 |
| Cd200 | 1.57 | 4.16E-03 |
| Pcdh9 | 1.57 | 1.37E-02 |
| AW146242 | 1.57 | 2.40E-02 |
| Rab31 | 1.57 | 9.41E-03 |
| Cyp39a1 | 1.57 | 3.35E-02 |
| Prr11 | 1.57 | 1.52E-03 |
| Casp8 | 1.57 | 1.95E-02 |
| Drp2 | 1.57 | 1.03E-02 |
| Ccl3 | 1.57 | 1.40E-02 |
| Crabp2 | 1.57 | 2.38E-02 |
| Snx33 | 1.57 | 1.53E-02 |
| Cd38 | 1.57 | 3.45E-02 |
| Lhcgr | 1.57 | 1.51E-04 |
| Cdv3 | 1.57 | 6.58E-03 |
| Tnnt1 | 1.57 | 2.35E-02 |
| Rnf144a | 1.57 | 2.94E-02 |
| Adamts3 | 1.56 | 2.14E-03 |
| Kcnh5 | 1.56 | 4.07E-02 |
| Notch1 | 1.56 | 1.61E-02 |
| Lhfp | 1.56 | 1.12E-03 |
| Plekho1 | 1.56 | 3.35E-03 |
| Tcf19 | 1.56 | 1.05E-02 |
| Spata6 | 1.56 | 1.01E-02 |
| Tyms | 1.56 | 1.32E-03 |
| Crispld1 | 1.56 | 2.94E-02 |
| Slc6a8 | 1.56 | 1.90E-03 |
| Pold3 | 1.56 | 3.85E-03 |
| Trim67 | 1.56 | 1.22E-02 |
| 4632417K18Rik | 1.56 | 3.60E-03 |
| Cdk5r1 | 1.56 | 6.72E-03 |
| Shisa4 | 1.56 | 3.84E-02 |
| Ugdh | 1.56 | 4.54E-02 |
| Ypel1 | 1.55 | 1.45E-02 |
| Hells | 1.55 | 2.41E-02 |
| Dnajb9 | 1.55 | 5.42E-03 |
| Elmo1 | 1.55 | 2.07E-02 |
| Oip5 | 1.55 | 1.42E-02 |
| Nudt10 | 1.55 | 1.34E-02 |
| Tgfb1i1 | 1.55 | 8.29E-03 |
| Zbtb45 | 1.55 | 2.83E-03 |
| Pou4f2 | 1.55 | 4.22E-04 |
| Mctp1 | 1.55 | 2.00E-02 |
| Rbm46 | 1.55 | 3.25E-02 |
| Csf1r | 1.55 | 2.15E-02 |
| Bend5 | 1.55 | 1.90E-02 |
| Esam | 1.55 | 3.76E-03 |
| BC062109 | 1.55 | 3.16E-02 |
| Cdh6 | 1.55 | 1.53E-02 |
| Synpr | 1.55 | 3.42E-02 |
| Rex2 | 1.55 | 1.90E-03 |
| Tbc1d2b | 1.55 | 4.27E-02 |
| Coch | 1.55 | 4.76E-02 |
| Mad2l1 | 1.54 | 9.98E-03 |
| Hn1 | 1.54 | 2.04E-02 |
| Kcnj2 | 1.54 | 1.21E-02 |
| Slc43a3 | 1.54 | 1.69E-02 |
| Fgf13 | 1.54 | 3.53E-03 |
| Notum | 1.54 | 1.96E-02 |
| Sgol1 | 1.54 | 2.44E-02 |
| Kif18a | 1.54 | 3.81E-02 |
| Mcm6 | 1.54 | 1.14E-02 |
| Zfp423 | 1.54 | 3.77E-04 |
| Pgm2l1 | 1.54 | 8.55E-03 |
| Tmod3 | 1.54 | 2.87E-02 |
| Arhgap19 | 1.54 | 2.64E-02 |
| Tmem108 | 1.54 | 1.87E-02 |
| 2610029I01Rik | 1.54 | 5.63E-04 |
| Snx15 | 1.54 | 3.25E-03 |
| Pgm1 | 1.54 | 1.58E-03 |
| Cyr61 | 1.54 | 3.71E-02 |
| Dtx1 | 1.54 | 4.54E-02 |
| Pcdh17 | 1.54 | 1.21E-02 |
| Phyhipl | 1.54 | 9.36E-03 |
| Hdgfrp3 | 1.54 | 3.00E-02 |
| Ttc28 | 1.54 | 7.14E-03 |
| Nelf | 1.54 | 1.80E-02 |
| EG668063 | 1.54 | 3.51E-02 |
| Anln | 1.53 | 1.53E-02 |
| Spint2 | 1.53 | 5.79E-03 |
| Lrrc4c | 1.53 | 2.60E-02 |
| Ncaph | 1.53 | 1.61E-02 |
| Tax1bp3 | 1.53 | 1.85E-02 |
| Etv1 | 1.53 | 6.44E-03 |
| Rgs16 | 1.53 | 5.94E-03 |
| Dgkh | 1.53 | 2.24E-02 |
| Samd4 | 1.53 | 2.54E-03 |
| Ccdc3 | 1.53 | 1.91E-02 |
| Hyls1 | 1.53 | 6.94E-03 |
| Ezh2 | 1.53 | 3.11E-02 |
| Nuf2 | 1.53 | 3.36E-02 |
| Irs4 | 1.53 | 1.99E-02 |
| Cobll1 | 1.53 | 4.49E-03 |
| Mn1 | 1.53 | 9.99E-03 |
| Slc9a3r1 | 1.52 | 2.82E-03 |
| Phactr2 | 1.52 | 2.02E-02 |
| Cdca8 | 1.52 | 6.75E-03 |
| Il10rb | 1.52 | 7.74E-03 |
| Plp2 | 1.52 | 2.39E-02 |
| Nrarp | 1.52 | 5.07E-03 |
| Ccng2 | 1.52 | 4.54E-02 |
| Col9a2 | 1.52 | 1.80E-02 |
| Ptchd1 | 1.52 | 3.07E-02 |
| L1cam | 1.52 | 1.34E-02 |
| Dock6 | 1.52 | 1.87E-02 |
| Efnb3 | 1.52 | 5.76E-03 |
| Tjp2 | 1.52 | 3.32E-02 |
| Pak1 | 1.52 | 9.32E-03 |
| Bdh1 | 1.52 | 4.23E-03 |
| Sgip1 | 1.52 | 9.62E-03 |
| Satb2 | 1.52 | 1.57E-02 |
| Lrrc8b | 1.52 | 9.30E-03 |
| 6530404N21Rik | 1.52 | 1.05E-02 |
| Eif4ebp2 | 1.52 | 4.86E-02 |
| Usp49 | 1.52 | 2.14E-02 |
| St6galnac3 | 1.52 | 4.89E-02 |
| Rlbp1l1 | 1.51 | 6.36E-03 |
| Pcna | 1.51 | 9.25E-03 |
| Ccnb2 | 1.51 | 3.52E-03 |
| Tnni1 | 1.51 | 8.23E-03 |
| Myadm | 1.51 | 3.91E-03 |
| Pola2 | 1.51 | 6.01E-04 |
| E030049G20Rik | 1.51 | 6.12E-03 |
| Hmgb2 | 1.51 | 8.01E-04 |
| Eltd1 | 1.51 | 1.43E-02 |
| BC013529 | 1.51 | 1.25E-02 |
| Plekho2 | 1.51 | 1.65E-02 |
| Baiap2l1 | 1.51 | 4.39E-02 |
| Fam126a | 1.51 | 2.96E-02 |
| Pdgfb | 1.51 | 1.20E-02 |
| Zdhhc15 | 1.51 | 1.93E-02 |
| Pcsk6 | 1.51 | 7.77E-03 |
| Cx3cr1 | 1.51 | 4.22E-02 |
| Usp51 | 1.51 | 3.09E-02 |
| EG245297 | 1.51 | 5.90E-03 |
| Spc25 | 1.51 | 8.35E-03 |
| Ctso | 1.51 | 7.40E-03 |
| EG544848 | 1.51 | 3.42E-03 |
| Plod1 | 1.51 | 3.75E-02 |
| Auts2 | 1.51 | 3.54E-03 |
| Atl1 | 1.51 | 7.22E-03 |
| Prim2 | 1.51 | 1.57E-02 |
| Golm1 | 1.50 | 1.70E-02 |
| Atp1b1 | 1.50 | 9.42E-03 |
| Ly6e | 1.50 | 5.25E-04 |
| Hist2h4 | 1.50 | 3.30E-02 |
| Chn2 | 1.50 | 1.11E-04 |
| Cacnb2 | 1.50 | 1.94E-02 |
| Fhl1 | 1.50 | 1.49E-02 |
| Mmp16 | 1.50 | 1.12E-02 |
| Rhog | 1.50 | 3.64E-02 |
| Rasa3 | 1.50 | 1.51E-02 |
| Palmd | 1.50 | 5.92E-03 |
| Tmem204 | 1.50 | 2.06E-02 |
| Slc24a4 | -1.50 | 1.61E-03 |
| Cog7 | -1.50 | 2.15E-02 |
| Mxd1 | -1.50 | 4.09E-03 |
| Snora61 | -1.50 | 2.34E-03 |
| Enpp5 | -1.50 | 1.01E-02 |
| Frmd3 | -1.51 | 6.58E-03 |
| Cpeb1 | -1.51 | 2.18E-03 |
| Psat1 | -1.51 | 2.10E-03 |
| Carhsp1 | -1.51 | 1.58E-02 |
| Cap2 | -1.51 | 1.88E-03 |
| OTTMUSG00000014243 | -1.51 | 2.74E-03 |
| Axin2 | -1.51 | 9.69E-03 |
| Ftsjd1 | -1.51 | 2.84E-03 |
| Rab11fip1 | -1.51 | 2.96E-02 |
| Cars | -1.51 | 5.73E-03 |
| D230039L06Rik | -1.51 | 3.49E-02 |
| Oat | -1.51 | 6.37E-03 |
| Cyb5d2 | -1.51 | 4.76E-02 |
| Duxbl | -1.51 | 5.86E-03 |
| Mrs2 | -1.51 | 4.76E-04 |
| Fem1a | -1.52 | 7.93E-03 |
| Kif26b | -1.52 | 9.06E-03 |
| Vps8 | -1.52 | 9.43E-03 |
| Gss | -1.52 | 2.57E-02 |
| Mboat1 | -1.52 | 1.54E-03 |
| 5730507A09Rik | -1.52 | 3.79E-02 |
| Igsf9 | -1.52 | 1.82E-03 |
| Aldh18a1 | -1.52 | 1.96E-02 |
| Abcc4 | -1.52 | 1.24E-03 |
| Lsamp | -1.53 | 1.82E-02 |
| Greb1 | -1.53 | 5.06E-03 |
| Pigb | -1.53 | 3.18E-03 |
| Igfals | -1.53 | 1.02E-02 |
| E130018O15Rik | -1.53 | 6.20E-04 |
| EG433332 | -1.53 | 1.89E-02 |
| Sned1 | -1.53 | 2.93E-04 |
| 5430407P10Rik | -1.53 | 3.31E-02 |
| Cyb5d1 | -1.54 | 1.75E-03 |
| 1810032O08Rik | -1.54 | 3.76E-03 |
| A930024E05Rik | -1.54 | 7.91E-03 |
| Grpel2 | -1.54 | 1.12E-03 |
| Snx1 | -1.55 | 4.01E-02 |
| Pxdn | -1.55 | 1.96E-02 |
| Nlrc5 | -1.55 | 2.55E-02 |
| D930030D11Rik | -1.55 | 1.94E-03 |
| Fam18a | -1.55 | 2.03E-03 |
| Gm1604b | -1.55 | 6.08E-03 |
| Podn | -1.55 | 2.84E-02 |
| Lrrc66 | -1.55 | 3.19E-02 |
| Gprc5c | -1.55 | 1.80E-03 |
| Frap1 | -1.55 | 8.45E-04 |
| Gpr137b-ps | -1.56 | 4.99E-03 |
| Usp46 | -1.56 | 2.17E-02 |
| EG449630 | -1.56 | 1.28E-02 |
| Osgin2 | -1.57 | 7.78E-03 |
| Tex2 | -1.57 | 1.39E-02 |
| 9930104L06Rik | -1.57 | 9.90E-04 |
| Aak1 | -1.57 | 1.01E-02 |
| Osbpl3 | -1.57 | 2.00E-04 |
| Top1mt | -1.58 | 2.39E-02 |
| Hspb3 | -1.58 | 1.11E-02 |
| Ogn | -1.58 | 2.24E-02 |
| Pigg | -1.58 | 1.94E-02 |
| Ttc9 | -1.58 | 8.91E-04 |
| BC043934 | -1.58 | 8.32E-03 |
| Tirap | -1.58 | 4.44E-03 |
| Naaa | -1.58 | 4.88E-02 |
| Slc30a7 | -1.60 | 1.22E-04 |
| Rap2b | -1.60 | 2.24E-02 |
| Nkap | -1.60 | 1.16E-02 |
| Plekha2 | -1.61 | 5.23E-03 |
| Jag1 | -1.61 | 5.87E-03 |
| Efcab1 | -1.61 | 2.01E-02 |
| Filip1 | -1.62 | 4.53E-02 |
| Stac2 | -1.62 | 4.33E-03 |
| Lpin1 | -1.62 | 3.07E-03 |
| Slc1a4 | -1.62 | 4.16E-03 |
| EG667203 | -1.62 | 2.69E-02 |
| Fam55d | -1.62 | 4.22E-02 |
| Abca8b | -1.62 | 5.27E-05 |
| Il1rap | -1.63 | 7.11E-03 |
| Tmprss5 | -1.63 | 1.08E-02 |
| Snord49b | -1.63 | 3.32E-02 |
| Rilpl1 | -1.63 | 2.89E-02 |
| ENSMUSG00000055849 | -1.63 | 1.46E-03 |
| Morn4 | -1.63 | 2.58E-04 |
| Kcnq4 | -1.64 | 1.31E-02 |
| 2610018G03Rik | -1.64 | 1.26E-02 |
| Exdl2 | -1.64 | 9.89E-03 |
| Mxra8 | -1.64 | 3.27E-02 |
| Epb4.9 | -1.64 | 4.77E-02 |
| AI848100 | -1.64 | 1.02E-03 |
| P4ha1 | -1.65 | 9.57E-03 |
| Snord33 | -1.65 | 3.70E-02 |
| Myo5a | -1.65 | 3.84E-04 |
| Rnf123 | -1.66 | 3.03E-04 |
| 1110032E23Rik | -1.66 | 1.92E-03 |
| Upf3b | -1.66 | 2.22E-02 |
| Dtna | -1.66 | 5.45E-04 |
| 2610109H07Rik | -1.66 | 8.84E-03 |
| Gpr137b | -1.67 | 1.24E-02 |
| Lmln | -1.67 | 7.99E-05 |
| Ldlr | -1.67 | 2.33E-02 |
| Klhl5 | -1.67 | 8.38E-04 |
| Mtap7 | -1.67 | 6.52E-03 |
| Tdrd9 | -1.67 | 1.88E-02 |
| Col4a6 | -1.68 | 8.63E-04 |
| Mthfd1l | -1.68 | 7.24E-03 |
| Ap3b2 | -1.68 | 4.35E-03 |
| Zim1 | -1.68 | 3.65E-02 |
| Gbf1 | -1.69 | 2.39E-04 |
| Cdh1 | -1.69 | 1.41E-02 |
| Tmem132a | -1.69 | 1.63E-03 |
| Gck | -1.70 | 8.01E-03 |
| Edn1 | -1.70 | 2.65E-02 |
| Nxnl2 | -1.70 | 1.14E-03 |
| Zfp109 | -1.71 | 4.55E-03 |
| Adam12 | -1.71 | 1.56E-03 |
| Bcl2l13 | -1.71 | 4.58E-03 |
| Pkp2 | -1.72 | 5.52E-03 |
| Nrcam | -1.72 | 1.60E-02 |
| Ppm1f | -1.72 | 2.83E-04 |
| Raver2 | -1.72 | 2.58E-03 |
| Fgfr2 | -1.73 | 7.14E-04 |
| Cpeb3 | -1.73 | 1.94E-02 |
| Aldh1l1 | -1.73 | 5.01E-03 |
| Lama1 | -1.73 | 2.46E-03 |
| Klhl21 | -1.73 | 5.66E-03 |
| Kif6 | -1.73 | 2.87E-03 |
| Adamtsl4 | -1.74 | 3.85E-02 |
| Kcnab2 | -1.74 | 1.05E-02 |
| Zfp322a | -1.74 | 5.96E-03 |
| Slc7a2 | -1.75 | 2.24E-02 |
| Zfp365 | -1.75 | 1.94E-03 |
| Snx22 | -1.75 | 4.48E-02 |
| Scnn1b | -1.75 | 2.09E-02 |
| Stx11 | -1.75 | 2.08E-04 |
| Cadm1 | -1.76 | 1.22E-02 |
| Tdrkh | -1.76 | 5.79E-05 |
| Slc15a1 | -1.78 | 1.01E-02 |
| Kcnh1 | -1.78 | 1.35E-02 |
| Tom1l1 | -1.79 | 4.43E-02 |
| Adam17 | -1.79 | 3.58E-04 |
| Oca2 | -1.79 | 4.46E-03 |
| Dync1i1 | -1.80 | 3.49E-02 |
| Npal3 | -1.80 | 2.56E-03 |
| Itga3 | -1.80 | 9.81E-03 |
| Prkca | -1.80 | 5.99E-03 |
| Prkaa2 | -1.80 | 3.05E-04 |
| Eml2 | -1.80 | 5.21E-03 |
| Crim1 | -1.81 | 1.80E-03 |
| Cpeb2 | -1.82 | 2.01E-03 |
| Fzd1 | -1.83 | 2.75E-02 |
| Col4a5 | -1.83 | 2.58E-03 |
| Tuft1 | -1.84 | 1.86E-02 |
| Dock5 | -1.84 | 7.48E-04 |
| Lrrtm3 | -1.85 | 1.68E-02 |
| Tspan5 | -1.85 | 2.76E-03 |
| Gata3 | -1.85 | 1.82E-02 |
| Spnb1 | -1.86 | 2.67E-03 |
| Orai2 | -1.86 | 6.22E-03 |
| Prx | -1.88 | 1.95E-03 |
| Lnp | -1.89 | 6.84E-04 |
| Pde4d | -1.89 | 1.80E-03 |
| Fktn | -1.89 | 4.26E-02 |
| Katnal2 | -1.90 | 1.09E-02 |
| Clu | -1.91 | 2.13E-02 |
| Tmprss11e | -1.91 | 1.05E-03 |
| Lrrtm2 | -1.91 | 4.60E-03 |
| AI452195 | -1.91 | 5.95E-03 |
| Pftk2 | -1.91 | 2.35E-02 |
| Vit | -1.92 | 2.44E-03 |
| Cyp7b1 | -1.93 | 1.80E-02 |
| Snora69 | -1.94 | 4.23E-03 |
| Bhlhe41 | -1.96 | 1.70E-02 |
| Mapk10 | -1.98 | 2.24E-04 |
| Lrtm1 | -2.00 | 1.81E-02 |
| Camk1d | -2.03 | 7.58E-04 |
| Myo6 | -2.03 | 1.67E-04 |
| Trpm3 | -2.03 | 2.90E-03 |
| BC032265 | -2.03 | 2.90E-03 |
| Nup210l | -2.04 | 5.83E-03 |
| 1700040L02Rik | -2.04 | 2.02E-02 |
| Ngef | -2.05 | 4.81E-02 |
| Ank2 | -2.05 | 4.81E-05 |
| 9030625A04Rik | -2.05 | 6.79E-03 |
| Susd2 | -2.06 | 1.78E-03 |
| Daam2 | -2.07 | 6.14E-03 |
| Dync2h1 | -2.08 | 2.19E-04 |
| Add2 | -2.08 | 1.51E-02 |
| Rgs13 | -2.08 | 3.96E-02 |
| B3gnt5 | -2.08 | 1.00E-02 |
| Gpr160 | -2.08 | 3.34E-02 |
| OTTMUSG00000005065 | -2.08 | 2.08E-03 |
| Med12l | -2.08 | 1.24E-02 |
| Flrt2 | -2.09 | 1.69E-05 |
| Kynu | -2.10 | 3.47E-02 |
| Rnf113a1 | -2.10 | 3.42E-02 |
| Scn11a | -2.11 | 7.01E-03 |
| Itga6 | -2.12 | 1.51E-03 |
| Cp | -2.14 | 3.51E-03 |
| Map6d1 | -2.16 | 1.37E-03 |
| Jph1 | -2.17 | 2.14E-03 |
| Clrn1 | -2.18 | 3.08E-02 |
| Atp8a2 | -2.21 | 3.95E-03 |
| Mtnr1a | -2.22 | 4.90E-02 |
| Crybb3 | -2.22 | 3.87E-02 |
| Wnt5b | -2.23 | 1.06E-02 |
| Gm1604A | -2.23 | 6.52E-03 |
| Angpt1 | -2.26 | 5.05E-03 |
| Mafb | -2.27 | 4.21E-02 |
| Plcb1 | -2.27 | 5.81E-03 |
| Cbfa2t2 | -2.28 | 7.99E-04 |
| Mcc | -2.30 | 7.13E-04 |
| Myo7b | -2.30 | 2.54E-02 |
| Slc38a5 | -2.32 | 1.46E-02 |
| Sema5a | -2.32 | 2.87E-02 |
| Ssx2ip | -2.32 | 1.81E-02 |
| Fam169a | -2.33 | 3.30E-04 |
| Ndrg4 | -2.34 | 1.26E-02 |
| Sulf1 | -2.38 | 3.21E-03 |
| Slc26a11 | -2.40 | 1.05E-03 |
| Amph | -2.41 | 5.40E-03 |
| Akr1b8 | -2.44 | 1.47E-02 |
| Ggt1 | -2.45 | 3.49E-02 |
| Sorbs1 | -2.48 | 1.26E-02 |
| Hfe | -2.48 | 6.27E-03 |
| Dnajc6 | -2.49 | 1.32E-02 |
| BC030476 | -2.49 | 4.64E-02 |
| Vldlr | -2.52 | 4.74E-03 |
| Ednrb | -2.57 | 6.65E-03 |
| Slc16a12 | -2.60 | 1.32E-02 |
| ENSMUSG00000074175 | -2.60 | 1.06E-02 |
| Aox4 | -2.63 | 2.25E-03 |
| Zbtb8b | -2.64 | 7.86E-03 |
| Ccdc80 | -2.75 | 4.05E-02 |
| Cntn6 | -2.78 | 1.93E-02 |
| Slc7a11 | -2.85 | 2.43E-02 |
| Sec14l5 | -2.87 | 5.48E-04 |
| S1pr5 | -2.87 | 6.92E-03 |
| Myo18b | -2.98 | 1.20E-03 |
| Trim9 | -3.01 | 2.31E-02 |
| Spnb3 | -3.04 | 1.55E-02 |
| Lix1 | -3.12 | 1.35E-02 |
| Gadd45g | -3.13 | 9.45E-03 |
| Unc13c | -3.14 | 6.12E-04 |
| Tmem132b | -3.19 | 5.21E-04 |
| Trpc6 | -3.37 | 9.48E-04 |
| Mylk4 | -3.37 | 5.86E-04 |
| Slit3 | -3.43 | 3.03E-02 |
| Ptgfr | -3.46 | 5.54E-03 |
| Epn3 | -3.50 | 4.93E-03 |
| Spry3 | -3.58 | 8.03E-03 |
| Adamts18 | -3.62 | 4.01E-03 |
| Necab1 | -3.97 | 4.60E-02 |
| Cabp5 | -4.05 | 2.05E-02 |
| Grm8 | -4.06 | 2.40E-03 |
| Pdgfra | -4.38 | 2.33E-02 |
| Pygm | -5.61 | 2.85E-02 |
