# Supplementary material for: Pax6 Regulates Gene Expression in the Vertebrate Lens through miR-204
Source: PLoS Genet. 2013 Mar 14;9(3):e1003357. doi: 10.1371/journal.pgen.1003357 (PMC3597499; doi:10.1371/journal.pgen.1003357)
Supplement: Table S2 — List of oligonucleotide probes used for ChIP and EMSA. (DOCX) [file pgen.1003357.s010.docx]

**Table S2.**

| Probe | Sequence (5'→3') | Genomic coordinates (mouse mm9 assembly) |
| --- | --- | --- |
| Amplicon- A2F | GGGTACCTGTGCTCACCACT | chr19:22,525,042-22,525,126 |
| Amplicon- A2R | AACACACACCACGGTGTTCA | chr19:22,525,042-22,525,126 |
| Amplicon- A1F | CAAAGCCAGGAGAGAAGTTTG | chr19:22,524,578-22,524,697 |
| Amplicon- A1R | TGTGTGTGTGTGTGTGTCTCTG | chr19:22,524,578-22,524,697 |
| Amplicon NSR-F | GGGGAGGTGGTATTCTGGTT | chr19:22,523,967-22,524,079 |
| Amplicon NSR-R | AACAGGGTCAGGGAAACTCA | chr19:22,523,967-22,524,079 |
| P6CON F | ggatgcaatttcacgcatgagtgcctcgagggatccacgtcga | - |
| P6CON R | tcgacgtggatccctcgaggcactcatgcgtgaaattgcatcc | - |
| Trpm3.1F | catctaacttcacccttccattattccactcactaatgtg | chr19:22,524,766-22,524,805 |
| Trpm3.1R | cacattagtgagtggaataatggaagggtgaagttagatg | chr19:22,524,766-22,524,805 |
| Trpm3.2F | ggggatgcgttttattagagaactttctccctaaatgacatctgtg | chr19:22,524,877-22,524,922 |
| Trpm3.2R | cacagatgtcatttagggagaaagttctctaataaaacgcatcccc | chr19:22,524,877-22,524,922 |
| Trpm3.3F | taaatggatgccacatcctttaagcactatttaggcacga | chr19:22,524,991-22,525,030 |
| Trpm3.3R | tcgtgcctaaatagtgcttaaaggatgtggcatccattta | chr19:22,524,991-22,525,030 |
| Trpm3.4F | agaactctgtcatcctttaaaattgaacacacaaacacac | chr19:22,524,627-22,524,666 |
| Trpm3.4R | gtgtgtttgtgtgttcaattttaaaggatgacagagttct | chr19:22,524,627-22,524,666 |
