# Supplementary material for: Pax6 Regulates Gene Expression in the Vertebrate Lens through miR-204
Source: PLoS Genet. 2013 Mar 14;9(3):e1003357. doi: 10.1371/journal.pgen.1003357 (PMC3597499; doi:10.1371/journal.pgen.1003357)
Supplement: Table S3 — Selection of putative Pax6 binding sites. Identification of putative Pax6 binding sites within the genomic region which is bound by Pax6 based on ChIP assay (mm9- chr19:22,524,578- Chr19:22,525,126). The putative Pax6 binding motifs were selected based on the sites identified using SELEX assay (Xie and Cvekl 2011, Epstein et al., 1994). The sites with up to 3 mismatches compared to the motifs were selected using fuzznuc program (http://mobyle.pasteur.fr/cgi-bin/portal.py?#forms::fuzznuc). The patterns and number of mismatch that were identified for each of the putative binding sequences are presented. (DOCX) [file pgen.1003357.s011.docx]

**Table S3.**

| **reference** | **sequence** | Putative Pax6 binding sites |
| --- | --- | --- |
| Xie and Cvekl, 2011 | TTYACGCATSA | 1) PAI/β/L Selex (1-1) |
| Xie and Cvekl, 2011 | WTTYACGCATNA | 2) PAI/β/L Natural (1-2) |
| Xie and Cvekl, 2011 | GCNTAATTRNTTM | 3) PAI/β/L/RED Selex (3-1) |
| Xie and Cvekl, 2011 | TTNNNNCNTAATTRNT | 4) PAI/β/L/RED Natural (3-2) |
| Epstein et al., 1994 | ATTATTNACRCATSR | 5) HD-PD (P6CON) |

(Y-C/T; S-G/C; W-A/T ;R-A/G ;N-A/T/G/C)

| Trpm3.1F | catctaacttcacccttccattattccactcactaatgtg | chr19:22,524,766-22,524,805 |
| --- | --- | --- |

Start End Strand Pattern_name Mismatch Sequence

9 19 + pattern1 3 TTCACCCTTCC

24 35 + pattern2 3 TTCCACTCACTA

| Trpm3.2F | ggggatgcgttttattagagaactttctccctaaatgacatctgtg | chr19:22,524,877-22,524,922 |
| --- | --- | --- |

Start End Strand Pattern_name Mismatch Sequence

24 39 + pattern4 2 TTTCTCCCTAAATGAC

25 40 + pattern4 3 TTCTCCCTAAATGACA

25 36 - pattern2 3 ATTTAGGGAGAA

| Trpm3.3F | taaatggatgccacatcct**ttaagc*actatttagg****cacga* | chr19:22,524,991-22,525,030 |
| --- | --- | --- |

Start End Strand Pattern_name Mismatch Sequence

19 29 + pattern1 3 TTTAAGCACTA

30 40 + pattern1 2 TTTAGGCACGA

18 29 + pattern2 3 CTTTAAGCACTA

29 40 + pattern2 2 ATTTAGGCACGA

20 35 + pattern4 3 **TTAAGCACTATTTAGG**

26 40 + pattern5 3 *ACTATTTAGGCACGA*

24 36 - pattern3 2 GCCTAAATAGTGC

15 30 - pattern4 3 ATAGTGCTTAAAGGAT

| Trpm3.4F | agaactctg**tcatcctttaa**aattgaacacacaaacacac | chr19:22,524,627-22,524,666 |
| --- | --- | --- |

Start End Strand Pattern_name Mismatch Sequence

10 20 - pattern1 3 **TTAAAGGATGA**

10 21 - pattern2 3 TTTAAAGGATGA

12 27 - pattern4 3 TTCAATTTTAAAGGAT
