# Supplementary material for: Pax6 Regulates Gene Expression in the Vertebrate Lens through miR-204
Source: PLoS Genet. 2013 Mar 14;9(3):e1003357. doi: 10.1371/journal.pgen.1003357 (PMC3597499; doi:10.1371/journal.pgen.1003357)
Supplement: Table S4 — Primers used for detection of transcript levels of specific genes using q-PCR. (DOCX) [file pgen.1003357.s012.docx]

**Table S4.**

| **DNA oligo nucleotide** | **species** | **sequence** |
| --- | --- | --- |
| TBP | Mouse | Forward 5’ CTTCGTGCAAGAAATGCTGAAT 3’  Reverse 5’ CAGTTGTCCGTGGCTCTCTTATT 3’ |
| Hprt | Mouse | Forward 5 GCGATGATGAACCAGGTTATGA 3’  Reverse 5’ ATCTCGAGCAAGTCTTTCAGTCCT 3’ |
| Myo10 | Mouse | Forward 5’ AGAGATCCTGACGCCTCTCA 3’  Reverse 5’ ATGGCCAATGAGTCTCTGCTA 3’ |
| Sox11 | Mouse | Forward 5’ CAGCTTCAAGAACATCACCAAG 3’  Reverse 5’ GCTGGATGAGGAGGTGGAC 3’ |
| Elavl3 | Mouse | Forward 5’ CAGACCAAGACCATCAAGGTG 3’  Reverse 5’ GCCACTGACATACAGGTTGG 3’ |
| HPRT | Human | Forward 5’ TGGACAGGACTGAACGTCTTGC 3’  Reverse 5’ TTATAGCCCCCCTTGAGCACA 3’ |
| CPN8 | Human | Forward 5’ TGC AAA ACT GCC TCC AGA TG 3’  Reverse 5’ GTA CAG ATT TCA GAC TCC TG 3’ |
| SATB2 | Human | Forward 5’ TTT AGC CAG CTG GTG GAG AC 3’  Reverse 5’ GCG TCG GGT GCA TCT GTC AC 3’ |
| HCN2 | Human | Forward 5’ GCT CAC AAC ACT CAG AGA CC 3’  Reverse 5’ TCA ATG TGT TCA GTG AAC CC 3’ |
| NFIA | Human | Forward 5’ CTACAGCATGAGTCCAGGAG 3’  Reverse 5’ TCACCAGGACTGTCCATTTC 3’ |
| MYO10 | Human | Forward 5’ GTCCTCAACAGCCTCTGCTC 3’  Reverse 5’ GTTTTGAATGGCACTGGACC 3’ |
| FBN2 | Human | Forward 5’ CTTGGCACATCTGGTTGTTG 3’  Reverse 5’TGTCAGAATGGTGGACGTTG 3’ |
| SOX11 | Human | Forward 5’GGAGCTGAGCGAGATGATCG3’  Reverse 5’GAACACCAGGTCGGAGAAGT 3’ |
| olHprt | Medaka | Forward 5 ‘ TCTGGAGAGGGTGTACATCC 3’  Reverse 5’GATGTAGTCCAACAGGTCGG 3’ |
| olMeis2 | Medaka | Forward 5’ CCATGGTCATGCCGATAGG 3’  Reverse 5’ TATGAGTGGAATGGGGATGAA 3’ |
| olSox11 | Medaka | Forward 5’ AAG AAG AGC GGC TCC AAG TC 3’  Reverse 5’ GGT AGT CCT CCT CGT AGT CG 3’ |
| olTrpm1 | Medaka | Forward 5’ AGCTCTCCAAAGCGATGGAG 3’  Reverse 5’ GGCTGTTGGAGAAGCATATC 3’ |
| olFbn2 | Medaka | Forward 5’ CTG CAG ACG AGG CTT CAT CC 3’  Reverse 5’ GTA GCC TCG TGG ACA AGT GC 3’ |
| olMyo10 | Medaka | Forward 5’ ATG GCG CCA TCA TGC ACA AC 3’  Reverse 5’ AGC TGG TAA GGG TTA ACT GC 3’ |
| olNfia | Medaka | Forward 5’ TGA GTC CAG GGG CCA TGA GG 3’  Reverse 5’ CTA CCT GGG GAG CGA CCT TG 3’ |
| olElavl3 | Medaka | Forward 5’ GGT AGC ATT GGA GAG ATT GAG 3’  Reverse 5’ CCG TTA AGC GTG TTG ATG GC 3’ |
