# Supplementary material for: Pax6 Regulates Gene Expression in the Vertebrate Lens through miR-204
Source: PLoS Genet. 2013 Mar 14;9(3):e1003357. doi: 10.1371/journal.pgen.1003357 (PMC3597499; doi:10.1371/journal.pgen.1003357)
Supplement: Table S5 — Sequences employed for functional studies of miR-204 in cell culture and in fish embryos. (DOCX) [file pgen.1003357.s013.docx]

**Table S5.**

|  | Sequence (5'→3') | Catalog number | Company |
| --- | --- | --- | --- |
| Scramble miR Mimic  (Neu2a) | AGUACUGCUUACGAUACGGUU | AM17110 | Ambion |
| hsa-miR-204 Mimic  (Neu2a) | UUCCCUUUGUCAUCCUAUGCCU | AM17000 | Ambion |
| hsa-miR-204 mimic  H36CE and fish | UUCCCUUUGUCAUCCUAUGCCU | C-300563-05-0005 | DHARMACON |
| ctrl cel-miR-67 mimic  H36CE and fish | UCACAACCUCCUAGAAAGAGUAGA | # CN-001000-01-05 | DHARMACON |
| ctrl cel-miR-67 inhibitor  H36CE | UCUACUCUUUCUAGGAGGUUGUGA | # IN-001005-01-20 | DHARMACON |
| hsa-miR-204 inhibitor  H36CE | AGGCAUAGGAUGACAAAGGGAA | IH-300563-07-0005 | DHARMACON |
| Mo-miR-204  fish | TTGATTCCAGGCATAGGATGACAAAGGGAAG | custom | GENE TOOLS |
| mm-Mo-miR-204  fish | TTGATTCCAGGCATAGGATGACAAAGGGAAG | custom | GENE TOOLS |
