# Supplementary material for: Pax6 Regulates Gene Expression in the Vertebrate Lens through miR-204
Source: PLoS Genet. 2013 Mar 14;9(3):e1003357. doi: 10.1371/journal.pgen.1003357 (PMC3597499; doi:10.1371/journal.pgen.1003357)
Supplement: Table S6 — The protein sequences of the Trpm1 and Trpm3 orthologues from various species for phylogenetic analysis of the Trpm1 and Trpm3 genes (Figure S6). (DOCX) [file pgen.1003357.s014.docx]

**Table S6.**

> NP_001238949.1 -hTrpm1

MSSFKRGSLKSSTSGSQKGQKSWIEKTFCKRECIFVIPSMKDSNRCCCGQFTNQHIPPLPSATPSKNEEESKQVETQPEKWSVAKHTQSYPTDSYGVLEFQGGGYSNKAMYIRVSYDTKPDSLLHLMVKDWQLELPKLLISVHGGLQNFEMQPKLKQVFGKGLIKAAMTTGAWIFTGGVSTGVISHVGDALKDHSSKSRGRVCAIGIAPWGIVENKEDLVGKDVTRVYQTMSNPLSKLSVLNNSHTHFILADNGTLGKYGAEVKLRRLLEKHISLQKINTRLGQGVPLVGLVVEGGPNVVSIVLEYLQEEPPIPVVICDGSGRASDILSFAHKYCEEGGIINESLREQLLVTIQKTFNYNKAQSHQLFAIIMECMKKKELVTVFRMGSEGQQDIEMAILTALLKGTNVSAPDQLSLALAWNRVDIARSQIFVFGPHWPPLGSLAPPTDSKATEKEKKPPMATTKGGRGKGKGKKKGKVKEEVEEETDPRKIELLNWVNALEQAMLDALVLDRVDFVKLLIENGVNMQHFLTIPRLEELYNTRLGPPNTLHLLVRDVKKSNLPPDYHISLIDIGLVLEYLMGGAYRCNYTRKNFRTLYNNLFGPKRPKALKLLGMEDDEPPAKGKKKKKKKKEEEIDIDVDDPAVSRFQYPFHELMVWAVLMKRQKMAVFLWQRGEESMAKALVACKLYKAMAHESSESDLVDDISQDLDNNSKDFGQLALELLDQSYKHDEQIAMKLLTYELKNWSNSTCLKLAVAAKHRDFIAHTCSQMLLTDMWMGRLRMRKNPGLKVIMGILLPPTILFLEFRTYDDFSYQTSKENEDGKEKEEENTDANADAGSRKGDEENEHKKQRSIPIGTKICEFYNAPIVKFWFYTISYLGYLLLFNYVILVRMDGWPSLQEWIVISYIVSLALEKIREILMSEPGKLSQKIKVWLQEYWNITDLVAISTFMIGAILRLQNQPYMGYGRVIYCVDIIFWYIRVLDIFGVNKYLGPYVMMIGKMMIDMLYFVVIMLVVLMSFGVARQAILHPEEKPSWKLARNIFYMPYWMIYGEVFADQIDLYAMEINPPCGENLYDEEGKRLPPCIPGAWLTPALMACYLLVANILLVNLLIAVFNNTFFEVKSISNQVWKFQRYQLIMTFHDRPVLPPPMIILSHIYIIIMRLSGRCRKKREGDQEERDRGLKLFLSDEELKRLHEFEEQCVQEHFREKEDEQQSSSDERIRVTSERVENMSMRLEEINERETFMKTSLQTVDLRLAQLEELSNRMVNALENLAGIDRSDLIQARSRASSECEATYLLRQSSINSADGYSLYRYHFNGEELLFEDTSLSTSPGTGVRKKTCSFRIKEEKDVKTHLVPECQNSLHLSLGTSTSATPDGSHLAVDDLKNAEESKLGPDIGISKEDDERQTDSKKEETISPSLNKTDVIHGQDKSDVQNTQLTVETTNIEGTISYPLEETKITRYFPDETINACKTMKSRSFVYSRGRKLVGGVNQDVEYSSITDQQLTTEWQCQVQKITRSHSTDIPYIVSEAAVQAEHKEQFADMQDEHHVAEAIPRIPRLSLTITDRNGMENLLSVKPDQTLGFPSLRSKSLHGHPRNVKSIQGKLDRSGHASSVSSLVIVSGMTAEEKKVKKEKASTETEC

> NP_001034193.2 -mmTrpm1

MGSMRKMSSSFKRGSIKSSTSGSQKGQKAWIEKTFCKRECIFVIPSTKDPNRCCCGQLTNQHIPPLPSGAPSTTGEDTKQADTQSGKWSVSKHTQSYPTDSYGILEFQGGGYSNKAMYIRVSYDTKPDSLLHLMVKDWQLELPKLLISVHGGLQSFEMQPKLKQVFGKGLIKAAMTTGAWIFTGGVSTGVVSHVGDALKDHSSKSRGRLCAIGIAPWGMVENKEDLIGKDVTRVYQTMSNPLSKLSVLNNSHTHFILADNGTLGKYGAEVKLRRQLEKHISLQKINTRLGQGVPVVGLVVEGGPNVVSIVLEYLKEDPPVPVVVCDGSGRASDILSFAHKYCDEGGVINESLRDQLLVTIQKTFNYSKSQSYQLFAIIMECMKKKELVTVFRMGSEGQQDVEMAILTALLKGTNASAPDQLSLALAWNRVDIARSQIFVFGPHWPPLGSLAPPVDTKATEKEKKPPTATTKGRGKGKGKKKGKVKEEVEEETDPRKLELLNWVNALEQAMLDALVLDRVDFVKLLIENGVNMQHFLTIPRLEELYNTRLGPPNTLHLLVRDVKKSNLPPDYHISLIDIGLVLEYLMGGAYRCNYTRKSFRTLYNNLFGPKRPKALKLLGMEDDEPPAKGKKKKKKKKEEEIDIDVDDPAVSRFQYPFHELMVWAVLMKRQKMAVFLWQRGEECMAKALVACKLYKAMAHESSESELVDDISQDLDNNSKDFGQLAVELLDQSYKHDEQVAMKLLTYELKNWSNSTCLKLAVAAKHRDFIAHTCSQMLLTDMWMGRLRMRKNPGLKVIMGILIPPTILFLEFRTYDDFSYQTSKENEDGKEKEEENVDANADAGSRKGDEENEHKKQRSIPIGTKICEFYNAPIVKFWFYTISYLGYLLLFNYVILVRMDGWPSPQEWIVISYIVSLALEKIREILMSEPGKLSQKIKVWLQEYWNITDLVAISMFMVGAILRLQSQPYMGYGRVIYCVDIILWYIRVLDIFGVNKYLGPYVMMIGKMMIDMLYFVVIMLVVLMSFGVARQAILHPEEKPSWKLARNIFYMPYWMIYGEVFADQIDLYAMEINPPCGENLYDEEGKRLPPCIPGAWLTPALMACYLLVANILLVNLLIAVFNNTFFEVKSISNQVWKFQRYQLIMTFHDRPVLPPPMIILSHIYIIIMRLSGRCRKKREGDQEERDRGLKLFLSDEELKKLHEFEEQCVQEHFREKEDEQQSSSDERIRVTSERVENMSMRLEEINERENFMKTSLQTVDLRLSQLEELSGRMVSALENLAGIDRSDLIQARSRASSECEATYLLRQSSINSADGYSLYRYHFNGEELLFEEPALSTSPGTAFRKKTYSFRVKDEDAKSHLDQPSNLHHTPGPSPPATPGRSRLALEGPLSTELRPGSDPGISAGEFDPRADFKSTEAAPSLNAAGVTGTQLTVESTDSHPLRESKLVRYYPGDPNTYKTMKSRSFVYTEGRKLVRGLSNWSAEYSSIMDQAWNATEWRCQVQRITRSRSTDIPYIVSEAASQDELEDEHRGSLLDPQISRSALTVSDRPEKENLLSVKPHQTLGFPCLRSRSLHGRPRSAEPAPSKLDRAGHASSTSNLAVMSVVPEGQNTQQEKRSAETEC

> NP_001032823.1 -RnTrpm1

MGSMRKMSSSFKRGSIKSSTSGSQKGQKAWIEKTFCKRECIFVIPSTKDPNRCCCGQLTNQHIPPLPSVT

PSSTAEDTKQGDAQSGKWSVSKHTQSYPTDSYGILEFQGGGYSNKAMYIRVSYDTKPDSLLHLMVKDWQL

ELPKLLISVHGGLQSFEMQPKLKQVFGKGLIKAAMTTGAWIFTGGVSTGVVSHVGDALKDHSSKSRGRLC

AIGIAPWGMVENKEDLVGKDVTRVYQTMSNPLSKLSVLNNSHTHFILADNGTLGKYGAEVKLRRQLEKHI

SLQKINTRLGQGVPVVGLVVEGGPNVVSIVLEYLREDPPVPVVVCDGSGRASDILSFAHKYCDEGGVINE

SLRDQLLVTIQKTFNYSKSQSHQLFAIIMECMKKKELVTVFRMGSEGQQDVEMAILTALLKGTNVSAPDQ

LSLALAWNRVDIARSQIFVFGPHWPPLGSLAPPVDTKVAEKEKKPPTATTKGRGKGKGKKKGKVKEEVEE

ETDPRKIELLNWVNALEQAMLDALVLDRVDFVKLLIENGVNMQHFLTIPRLEELYNTRLGPPNTLHLLVR

DVKKSNLPPDYHISLIDIGLVLEYLMGGAYRCNYTRKSFRTLYNNLFGPKRPKALKLLGMEDDEPPAKGK

KKKKKKKEEEIDIDVDDPAVSRFQYPFHELMVWAVLMKRQKMAVFLWQRGEECMAKALVACKLYKAMAHE

SSESELVDDISQDLDNNSKDFGQLAVELLDQSYKHDEQVAMKLLTYELKNWSNSTCLKLAVAAKHRDFIA

HTCSQMLLTDMWMGRLRMRKNPGLKVIMGILIPPTILFLEFRSYDDFSYQTSKENEDGKEKEEENVDANA

DAGSRKGDEENEHKKQRSIPIGTKICEFYNAPIVKFWFYTISYLGYLLLFNYVILVRMDGWPSPQEWIVI

SYIVSLALEKIREILMSEPGKLSQKIKVWLQEYWNITDLVAISMFMVGAILRLQNQPYMGYGRVIYCVDI

ILWYIRVLDIFGVNKYLGPYVMMIGKMMIDMLYFVVIMLVVLMSFGVARQAILHPEEKPSWKLARNIFYM

PYWMIYGEVFADQIDRKTRIHIYAMEINPPCGENLYDEEGKRLPPCIPGAWLTPALMACYLLVANILLVN

LLIAVFNNTFFEVKSISNQVWKFQRYQLIMTFHDRPVLPPPMIILSHIYIIVMRLSGRCRKKREGDQEER

DRGLKLFLSDEELKKLHEFEEQCVQEHFREKEDEQQSSSDERIRVTSERVENMSMRLEEINERENFMKAS

LQTVDLRLSQLEELSGRMVGALENLAGIDRSDLIQARSRASSECEATYLLRQSSINSADGYSMYRYHFNG

EELLFEEPALSTSPGTVFRKKTCSFRVKEEDVKPHLDQPSSLHHTPGPSPPATPGRSRLALDGPLSTELR

PGLDPGISAGELDPRADFKSAEVAPSLNTANVASTQLTVESTVSHPLRESKLARYYPGDLNTYKTMKSRS

FVYSEGRKLVRGLSNWGAEYSSIMDQTWNSAEWRCQVQRITRSRSTDIPYIVSEAASQDEFEDEHRESLL

APQISRSALTVSDRPEKENLLSVKPHQTLGFPCLRSRSLHGHPRSAKPSPSKLDRAGHASSTSNLAVMSD

APEGQNTQQEKGNPETEC

> XP_002696651.1 -BtTrpm1

MGAHVLLMWTPPLKGLALLCQPPAWPEEEAERGQLHPLGPALGQGQKAWIEKTFCKRECIFVIPSTKDPN

RCCCGQLTNQHIPPLPSVTASKNGEENKLVEVHPEKWSVGKHTQSYPTDSYGILEFQGGGYSNKAMYIRV

SYDTKPDSLLHLMVRDWQLELPKLLISVHGGLQNFEMQPKLKQVFGKGLIKAAMTTGAWIFTGGVSTGVI

SHVGDALKDHSSKSRGRVCAIGIAPWGIVENKEDLVGRDVTRVYQTMSNPLSKLSVLNNSHTHFILADNG

TLGKYGAEVMLRRQLEKHISLQKINTRLGQGVPLVGLVVEGGPNVVSIVLEYLREEPPVPVVVCDGSGRA

SDILSFAHKYSEEGGIINDSLRDQLIVTIQKTFNYNKTQSHQLFAMIMECMKKKELVTVFRMGSEGQQDI

EMAILTALLKGTNASAPDQLSLALAWNRVDIARSQIFVFGPRWPPLGSLAPPTDSKATEKEKKPPAATTK

GGRGKGKGKKKGKVKEEVEEETDPRKLELLNWVNALEQAMLDALVLDRVDFVKLLIENGVNMQHFLTIPR

LEELYNTRLGPPNTLHLLVRDVKKGNLPPDYHISLIDIGLVLEYLMGGAYRCNYTRKSFRTLYNNLFGPK

RPKALKLLGMEDDEPPAKGKKKKKKKKEEELDIDVDDPAVSRFQYPFHELMVWAVLMKRQKMAVFLWQRG

EESMAKALVACKLYKSMAHESSESELVDDISQDLDNNSKDFGQLAVELLDQSYKHDEQIAMKLLTYELKN

WSNSTCLKLAVAAKHRDFIAHTCSQMLLTDMWMGRLRMRKNPGLKVIMGILLPPTILFLDFRTYDDFYQT

SKENEDGKEKEEENMDVNADASSRKGDEENEHKKQRSVPIGTKICEFYNAPIVKFWFYTISYLGYLLLFN

YVILVRMERWPCLQEWIVISYILSLALEKIREILMSEPGKLSQKIKVWLQEYWNITDLVAISMFMIGAIL

RLQKQPYMGYGRVIYCVDIIFWYIRVLDIFGVNKYLGPYVMMIGKMMIDMLYFVVIMLVVLMSFGVARQA

ILHPDEEPSWKLARNIFYMPYWMIYGEVFADQIDLYAMEINPPCGDNQYDEEGKRLPPCIPGAWLTPAIM

ACYLLVANILLVNLLIAVFNNTFFEVKSISNQVWKFQRYQLIMTFHDRPVLPPPMIILSHIYIIIMRLSR

RCRKKREGDQDEQDRGLKLFLSDEELKRLHEFEEQCVREHFQEKEDEQQSSSEERIRVTCERVENMSMRL

EEINERENFMKTSLQTVDLRLSQLEELSNRMVNALENLAGIDKSDLIQTRSRASSECDATFLLRRSSISS

ADGYSLYRYHFNGDELLYEDTSFCMPPGAASRKKAGSFRGKEEKDQRTYLAPERQGSLRLPPSTGAPASP

DRGQLASDNCKSSSEPKSGPDTGISAENDERQVDTKKEEMTSPNLSQTDAIHGEDKSDFQKSTQLTVETT

KVESTISYPLEETKTSHYYPDETFSACETLKSRSFVYSRGRKVVGGANNWGTAGDPTCPSTVQQWTTEWK

YEVQKIMRSRSTEIPSFASEAALQAEHCGHFTDKEDENCVLKTAPQISRLSVTDRTDKENLLSVKTHQTL

GFPSSRSKSLHGRPRSVKALQGNLHRPGHASSVSNLVVVSGITMEDPKVQQEKTSSETEC

> XP_425066.2 -ggTrpm1

MKGRGGKFTGSLRRMSSSFKRTSFKGSASGSQKGQKAWIEKTFSKRECIYVIANNKDISRCCCGQLITQH

IPPPPSTTANKNGEETKQVEAQPEKWSVSKHTQTYPTDAYGNLEFQGGGHSNKAMYIRVSYDTKPDSLLH

LMVKDWQLELPKLLISVHGGLQNFEMQPKLKQVFGKGLIKAAMTTGAWIFTGGVSTGVIRHVGDALKDHS

SKSRGRICAIGIAPWGIVENKEDLIGKDVTRVYQTMSNPLSKLSVLNSSHTHFILADNGTLGKYGAEVKL

RRQLEKHISLQKINTRLGQGVPVVGLIVEGGPNVISIVLECLREEPPLPVVICDGSGRASDILSFAHKYS

EEGGIISESLRDQLLVTIQKTFNYSRNQAHQLFIILMECMKKKELITVFRMGSEGQQDIEMSILTALLKG

TNASAPDQLSLALAWNRVDIARSQIFVFGHHWPPLGSLTAGDGTAPEKEKKSPAVQTKAARGKGKGKKKG

GKVKEEPEEETDPRKLELLNWVNSLEQAMLDALVLDRVDFVKLLIENGVNMQHFLTIPRLEELYNTRLGP

PNTLHLLVRDVKKGNLPPDYHISLIDIGLVLEYLMGGAYRCNYTRKSFRTLYNNLFGPKRPKALKLLGME

DDEPPTKGKKKKKKKEEEIDIDVDDPEVSRFQYPFHELMLWAVLMKRQKMALFLWQRGEETMAKALVACK

LYKSMAHESSESELVDDISQDLDNNSKDFGQLAVELLDQSYKHDEQIAMKLLTYELKNWSNSTCLKLAVA

AKHRDFIAHTCSQMLLTDMWMGRLRMRKNPGLKVIMGILFPPTILFLEFRSYDDYSYQTSRENEEGKEKE

EENVDANVDTGSRKGDEENGNKKQNSLPIGTKIYEFYNAPIVKFWFYTISYLGYLMLFNYIILVRMERWP

SVQEWIVISYIVTLALEKVREILMSEPGKLSQKVKVWLQEYWNITDLVAISVFMIGAILRLQNQPYMGYG

RVIYCVDIIFWYIRVLDIFGVNKYLGPYVMMIGKMMIDMLYFVVIMLVVLMSFGVARQAILHPDEEPSWR

LARNIFYMPYWMIYGEVFADQIDLYAMEINPPCGDNLYDEDGKRLPPCIPGAWLTPAIMACYLLVANILL

VNLLIAVFNNTFFEVKSISNQVWKFQRYQLIMTFHDRPVLPPPMIIFSHLYIIIKRVCCRCKKREGDQDE

RDRGLKLFLNDEELKKLYEFEEQCVEEYFQEKEDEQQSSNDERIRVTSERVENMSMRLEEVNEREHFMKA

SLQTVDLRLSQLEELSGRMVNALEKLAGVDKSELTYTRSRASSECDAAYLLRQSSVNSSDGYSMYRYHVG

GDELAYDDSTTPMSPAIGSRKKAHSVGTKEDGADPRMLVPEHHTSLHYTSSANAVTPADYSTSALDIAQN

VSSPHSGSGGLGDENQGIFKKDEMPPQSLNQMDVIMNRQSVPRPDFQNTQLKVERTKLEATISYPLDKSK

AMRYFPPETFSACQTTMTKSRSFIFAQGGKLVGGVNNWTTEYSTIMDQVCPSTIEQWATEWKYEVEQQLS

QERPPEYPGIISEAERQAEQKQLQMDTDDDSDVGEAGISASYASMPATVKTQTENLLSVKPERTSGFPSV

RSKSLHSHSRKAKSIKDKLNRPGHATSVTNLVVAFGSATEEQKARQEIASTETEC

> XP_002934962.1 -XtTrpm1

MEEKGGGEGGENEKPSRILEVTQRCWHPISSGSLTTSWQSTEEARQHSSSTAFSVKQRRRTSEGQKAWIE

KTFSKRECIYVLANSKDSNRCCCGQLLTQHIPVPQNATGNKNGEECKQLEAVPEKWSISKHTQLSPTDAF

GILEFQGGGHSNKAMYIRVSYDSKPDSLLHLMVKEWQLELPKLLISVHGGLQNFEMQPKLKQVFGKGLIK

AAMTTGAWIFTGGVSTGVIRHVGDALKDHSSKSRGRICAIGIAPWGIVENKEDLIGKDVTKPYQTMSNPL

SKLSVLNSSHTHFILADNGTLGKYGAEVKLRRQLEKHISLQKINTRLGQGVPVVALIVEGGPNVITIVLE

CLREEPPVPVVVCDGSGRASDIMSFAHKYSEEGGIISESLRDQLLVTIQKTFNYSRNQAHQLLVVLMECM

KKKELITVFRMGSEGQQDIEMAILTALLKGTNASAPDQLSLALAWNRVDIARSQIFVFGHHWPPLGSLTA

ADGTTQDKDKKSPAAQPKATRGKGKGKKKGKGKEEPAEETDPRKIELLNWVSSLEQAMMDALVLDRVDFV

KLLIENGVSMQRFLTIPRLEELYNTRLGPPNTLHYIVRDVKKGNLPPDYHVSLIDIGLVLEYLMGGAYRC

HYTRKTFRTLYNNLFGPKRPKALKLLGMEDDEPPTKGKKKKKKKEEEIDMDVDDPEVSRFKYPFHELMVW

AVLLKRQKMALFLWQRGEETMAKALVACKLYKSMAHESSESELVDDISQDLDNNSKDFGQLALELLDQSY

KHDEQVAMKLLTYELKNWSNSTCLKLAVAAKHRDFIAHTCSQMLLTDMWMGRLRMRKNPGLKVIMGILFP

PTILFLEFRSQDDLSFHTSKEYEEGKEKEDENMDGNADTTSRKGDEENGNRKQKSIPIGTKIYEFYNAPI

VKFWFYTISYLAYLMLFNYVILVKMERWPSVQEWVVISYIVTLALEKVREILMSEPGKLSQKVKVWLQEY

WNITDLVAISVFLIGAILRLQNQPYMGYGRVIYCVDIIFWYIRVLDIFGVNKYLGPYVMMIGKMMIDMLY

FVIIMLVVLMSFGVARQAILHPDEEPSWRLARNIFYMPYWMIYGEVFADQIDLYAMEINPPCGENLYDED

GKRLPPCIPGAWLTPALMACYLLVANILLVNLLIAVFNNTFFEVKSISNQVWKFQRYQLIMTFHDRPVLP

PPMIIFSHLYIILKRICCRCKKIQEGDQDERDRGLKLFLNDEELKKLYEFEEQCVEEYFQEKEDEQQSSN

DERIRVTSERVENMSMRLEEVNEREHFMKASLQTVDLRLSQLEELSGRMVNALERLAGIDKSELINERSR

ASSVCDAAFLLRQGSVNSSDGTSVYKYHVETDEVTCDESTGSISPALGLRRKAYSFGMRTEKGEQDNKAQ

LVPRQASLRYASSANAVATKDANKVTLGIVSKRPISCVDIWIPECDKKETINESPSTQHLSQTDIVTNCQ

SEGEVTEVSKSEFANSHLTVERTKLEATISFPLERSKAMKYYSGQSFNTCQTTMTKSRSFVFTEGGKLVG

GVNNWTEYSTIIDQVSPTSLEEWTDQWNNQGDSPRKQSPELATIISEAEIQAELKPLLTDTEDDSDIGET

GNSASYPCTERTETKNLLSVQLDRTCGFSSPRSKSLHSHTGKSRSLIDKLERPGHASSVMNIVVACGSVT

EDTKIKQENASTETEC

> XP_003440349.1 -DrTrpm1a

MGQKVWIEKTFHKRECNHIFPAKDPTRCACGYLVAQHTAVANKPSEENQLVQVEPPQEKWSVVRHTQLTPTDAYGIIEFQGGGFINKAMVSNVSHDTKPDSLLHLMVKEWQLELPTLLISVHGGLQNFDLQPKLKQVFGKGLIKAAVTTGAWIFTGGVSTGVIRHVGDALKDHSSKSRGKVCAIGIAPWGILENKEDLIGKDVNKPYQAISNPLSKLAVLNNSHSHFILSDNGTCGKYGAEVKLRRQLEKHISLQKINTRLGQGVPVVCLIVEGGPNVISIALESLREEPPVPVVVCDGSGRASDIISFAHKYSEVEGLVNEDAKEQLLVTIQKTFNYNKMQSGQILLMVLECMKKRELITVFRMGAEGQDDIEMAILTALLKGTNASAPDQLSLALAWNRVDIARNQIFVYGHNLPPASALASAITISAPSQEKQKGATQRNKGKARGKKGKGGKAKPEQPEETDPRKLELLNWVNSLEQAMMDALVLDRTDFVKLLIENGVNIHHFLTIPRLEELYNTELNSNNTLHILVRDVKKGNLPPDYQITLIDIGLVVEYLMGGAYRCNYTRKNFRALYNNLYGLKRPKALKLLGMEDDEPRDIDVDDPEVSRFHYPFHELMVWAVLMKRQKMALFLWQRGEEGMAKALVACKLLKGMAHESSQSEMVDDISQDLDNNSKEFGTLAYELLDQSYKHDEQLAMKLLTYELKNWSNSTCLKLAVAAKHRDFIAHTCSQMLLTDMWMGSLRMGKNPGLKVILSLIFPPFILLLDFRLGDDTSHQVAVADEDKKTKDDERSTRDANADAASKKGDEEDGNKKMRRIPIGTKIFEFYNAPFTKFWFNTISYLGYLMLYNYVVLVKMERWPSLQEWIVISYIITLGLEKVRQILMSEPGKLKQKINVWLEEYWNITDLVAISVFLLGLLLRLQNEPYMGYGRVIYCVDIIFWYIRVLDIFGVNKYLGPYVMMIGKMMIDMLYFVVIMLVVLMSFGVARQAILHPDEEPTWRLARNIFYMPYWMIYGEVFADSIDLYAMEINPPCGENLYDEDGKKLPPCIPGAWLTPAIMACYLLVANILLVNLLIAVFNNTFFEVKSISNQVWKFQRYQLIMTFHDRPVLPPPLIILSHLYILFRKLCCRCSKKKEGELDEKDKGLKLILTPDELKMLYEFEEQCVEEYFREKEDEQLSSNDERIRVTNERVENMSMRLEEVNERENTMKASLQTVDLRLSQLEDINGRMVNALERLVGIDHSELTRSRSTASSICDPSSLQRHSSINSADGYSLYRYYLGVDERPPEE

> XP_003437779.1 -DrTrpm1b

MPTDSYGIIEFQGGGHINKAMYIRVSYDTKPDNLLHLMVKDWQLELPTLLISVHGGLQNFDLQPKLKQVFGKGLIKAAVTTGAWIFTGGVSTGVIRHVGDALKDHSSKSRGKVCAIGIAPWGIVENKEDLIGRDVTRPYQTMSNPLSKLSVLNNSHSHFILADNGTHGKYGAEVRLRRQLEKHISLQKINTRLGQGVPLVCLILEGGPNVISIVLESLREDPPVPVVVCDGSGRASDIISFAHKYSEQDGLVNDSVRDQLLVTIQKTFNYNRNQAQQIYLMVMECMKKRELITVFRTASEGQQDIEMAILTALLKGTNASAPDQLSLALAWNRVDIARSQIFVHGQHWPPAGSLPTSSTGQQDKPKSPIATRVSKGKPARGKKGKGAKSKPEPPEETDPRKLELLNWVNSLEQAMMDALVLDRVDFVKLLIENGVNIHHFLTIPRLEELYNTRLGPINTLHFVVRDVKKGNLPPDYQITLIDIGLVLEYLMGGAYRCNYTRKSFRTLYNNLYGLKRPKALKLLGMEDDDPRPKGKKKMKKKKEEEIDIDVDDPEVSRFQYPFHELMVWAVLMKRQKMALFLWQRGEEAMAKALVACKLYKAMAHESSRSELVDDISQDLDNNSKDFGQLAYELLDQSYKHDEHMAMKLLTYELKNWSNSTCLKLAVAAKHRDFIAHTCSQMLLTDMWMGCLRVGKSNGLKVILGIIFPPAILLLDFRTGDDLSYQNSKDKEEVKDKDDDTKSTKDGTVSMDATSKKGDEEDGKKKQKRLPVGKKIYYFFNAPYTKFWFNTTAYLVYLMLYNYIILVKMERWPSLQEWIVISYIITLGLEKVRQILMSEPGKLKQKINVWLEEYWNITDLAAITTFLMGLLLRLQNEPYMGYGRVIYCVDIIFWYIRVLDIFGVNKYLGPYVMMIGKMMIDMLYFVVIMLVVLMSFGVARQAILHPDEEPTWRLARNIFYMPYWMIYGEVFADSIDLYAMEINPPCGENMYDEDGKKLPPCIPGAWLTPAIMACYLLVANILLVNLLIAVFNNTFFEVKSISNQIWKFQRYQLIMTFHDRPVLPPPLIIFSHIYIVLKRLCCRCRKKQEGELDDRDHGLKLTLSAEELKSLYEFEEQNVEEYFREKEDEVQSSNDERIRITSQRVENMSMRLEEVNEREHTMKASLQTVDLRLAQLEEFSGRMMHALERLAGIDRCDLVRTRSGSSMAVDQSGLLRRGSVTSADGYSLYRWHLDAEERGDEVRGQGTERRSSIGITDLNPQHASSYGPTLDVLPLRQRTHSSSSVDILISPCESFPPQDGGQNPQSPTRSSCQPTKDARALLESRIDTAVSHPLERAQSLRQYPNEAQNNSLSHENRSQSGTLYVSMAQSKLSSPGNTWASEPYGLQDQANRSPTLGRWPPRFDYKVHPSPFGLSPKTSSEKLRQVDDQDKEDTSKESKRTNSHQAGSEEKNKDEDEIQANNESTKTEEVDDKREKLTDSDRLYVSEDRMYPALRSKSLNANPRKVKAAGDVLDKPRAASSVRDLAEAFEVNTNDYRPSRERSGTQT

> XP_003440349.1 -olTRPM1

MNKAMYIRVSYDTKPDNLLHLMVKDWQLELPTLLISVHGGLQNFDLQPKLKQVFGKGLIKAAVTTGAWIFTGGVNTGVIRHVGDALKDHSSKSRGKVCAIGIAPWGILENKEDLIGKDVTRPCQSMANPLSKLAVLNNSHSHFILTDNGTCGKYGSEVKLRRLLEKHISLQKINTRLGQGVPLVCLIVEGGPNVISIALESLRDEPPIPVVICDGSGRASDIISFAHKFSEDGGLVNDDVRDQLLVTIQKTFNYSKSQSQQILLMVMECMKKRELITVFRMGSEGQQDIEMAILTALLKGTNASAADQLSLALAWNRVDIARNHIFVYGHNLPVSYSVANTTTSGAAAQEKPKSPASAPRSKARPKKGKGKGKAKPEPPEETDPRKLELIRWVNSLEQAMMDALVLDRVDFVKLLLENGVNIHHFLTIPRLEELYNTKLGPANTLHVVVRDVKKGNLPPDYQITLIDIGLVLEYLMGGAYRSNYTRKAFRNLYNTLYGLKRPKALKLLGMEDDEPRTKGKKKAKKKKEEEVEIDVDDPEVCRFKYPFHELMLWAVLLKRQKMALFLWQRGEEAMAKALVACKLYKGMAHECSESELVDDISQDLENNSKEFGQLAYELLDQSYKHDEQVAMKLLTYELVNWSNSTCLKLAVAAKQRDFIAHTCSQMLLTDMWMGCLRIGKSPGLKVILGIIFPPMILLLDFRIGDDASYQTPGGKSEGKDKDEDTKDPNTDATSRKGDEEEGSTKIRKVPIGKRIFEFYDAPFTKFWFNTISYLGYLMLYNYIILVKMERWPSIQEWTVISYILTLGTEKVRQILMSEPGKLKQKISVWLEDYWNITDLVAICTFLFGMMLRLQNEPYLGYGRVIYCIDIIFWYIRVLDIFGVNKYLGPYVMMIGKMMIDMMYFVVIMLVVLMSFGVARQAILHPDEEPTWRLARNIFYMPYWMIYGEVFADSIDLYAMEINPPCGDQLYDEDGKKLPPCIPGAWLTPAIMACYLLVANILLVNLLIAVFNNTFFEVKSISNQVWKFQRYQLIMTFHDRPILPPPLIILSHLYILFSRLFRRCVKKKQEGELDEKDRGLKLRLNPEELKSLYEFEEQCVEEYFRERDDEKQSSSDERIKVTSERVENMSMRLEEVNERENTMKASLQTVDLRLAQLEDIHGRMATALEKLAGLDKLELTRTFSRNSSVCDPSSLLRQGSINSADGYSLYRFHMDMEEFASKQKDTDEKSG

> NP_996829.3 -hTRPM3

MYVRVSFDTKPDLLLHLMTKEWQLELPKLLISVHGGLQNFELQPKLKQVFGKGLIKAAMTTGAWIFTGGVNTGVIRHVGDALKDHASKSRGKICTIGIAPWGIVENQEDLIGRDVVRPYQTMSNPMSKLTVLNSMHSHFILADNGTTGKYGAEVKLRRQLEKHISLQKINTRCLPFFSLDSRLFYSFWGSCQLDSVGIGQGVPVVALIVEGGPNVISIVLEYLRDTPPVPVVVCDGSGRASDILAFGHKYSEEGGLINESLRDQLLVTIQKTFTYTRTQAQHLFIILMECMKKKELITVFRMGSEGHQDIDLAILTALLKGANASAPDQLSLALAWNRVDIARSQIFIYGQQWPVGSLEQAMLDALVLDRVDFVKLLIENGVSMHRFLTISRLEELYNTRHGPSNTLYHLVRDVKKGNLPPDYRISLIDIGLVIEYLMGGAYRCNYTRKRFRTLYHNLFGPKRPKALKLLGMEDDIPLRRGRKTTKKREEEVDIDLDDPEINHFPFPFHELMVWAVLMKRQKMALFFWQHGEEAMAKALVACKLCKAMAHEASENDMVDDISQELNHNSRDFGQLAVELLDQSYKQDEQLAMKLLTYELKNWSNATCLQLAVAAKHRDFIAHTCSQMLLTDMWMGRLRMRKNSGLKVILGILLPPSILSLEFKNKDDMPYMSQAQEIHLQEKEAEEPEKPTKEKEEEDMELTAMLGRNNGESSRKKDEEEVQSKHRLIPLGRKIYEFYNAPIVKFWFYTLAYIGYLMLFNYIVLVKMERWPSTQEWIVISYIFTLGIEKMREILMSEPGKLLQKVKVWLQEYWNVTDLIAILLFSVGMILRLQDQPFRSDGRVIYCVNIIYWYIRLLDIFGVNKYLGPYVMMIGKMMIDMMYFVIIMLVVLMSFGVARQAILFPNEEPSWKLAKNIFYMPYWMIYGEVFADQIDPPCGQNETREDGKIIQLPPCKTGAWIVPAIMACYLLVANILLVNLLIAVFNNTFFEVKSISNQVWKFQRYQLIMTFHERPVLPPPLIIFSHMTMIFQHLCCRWRKHESDPDERDYGLKLFITDDELKKVHDFEEQCIEEYFREKDDRFNSSNDERIRVTSERVENMSMRLEEVNEREHSMKASLQTVDIRLAQLEDLIGRMATALERLTGLERAESNKIRSRTSSDCTDAAYIVRQSSFNSQEGNTFKLQESIDPAGEETMSPTSPTLMPRMRSHSFYSVNMKDKGGIEKLESIFKERSLSLHRATSSHSVAKEPKAPAAPANTLAIVPDSRRPSSCIDIYVSAMDELHCDIDPLDNSVNILGLGEPSFSTPVPSTAPSSSAYATLAPTDRPPSRSIDFEDITSMDTRSFSSDYTHLPECQNPWDSEPPMYHTIERSKSSRYLATTPFLLEEAPIVKSHSFMFSPSRSYYANFGVPVKTAEYTSITDCIDTRCVNAPQAIADRAAFPGGLGDKVEDLTCCHPEREAELSHPSSDSEENEAKGRRATIAISSQEGDNSERTLSNNITVPKIERANSYSAEEPSAPYAHTRKSFSISDKLDRQRNTASLRNPFQRSKSSKPEGRGDSLSMRRLSRTSAFQSFESKHN

> AEE80504.1 -mmTRPM3

MPGPWGTVYFLGTAQICSFLSSRWNLEGVMNQTDASRPLNWTIRKLCHAAFLPSVRLLKAQKSWIERAFYKRECVHIIPSTKDPHRCCCGRLIGQHVGLTPSISVLQNEKNESRLSRNDIQSEKWSISKHTQLSPTDAFGTIEFQGGGHSNKAMYVRVSFDTKPDLLLHLMTKEWQLELPKLLISVHGGLQNFELQPKLKQVFGKGLIKAAMTTGAWIFTGGVNTGVIRHVGDALKDHASKSRGKICTIGIAPWGIVENQEDLIGRDVVRPYQTMSNPMSKLTVLNSMHSHFILADNGTTGKYGAEVKLRRQLEKHISLQKINTRIGQGVPVVALIVEGGPNVISIVLEYLRDTPPVPVVVCDGSGRASDILAFGHKYSEEGGLINESLRDQLLVTIQKTFTYTRTQAQHLFIILMECMKKKELITVFRMGSEGHQDIDLAILTALLKGANASAPDQLSLALAWNRVDIARSQIFIYGQQWPVGSLEQAMLDALVLDRVDFVKLLIENGVSMHRFLTISRLEELYNTRHGPSNTLYHLVRDVKKREYPGFGWIYFKGNLPPDYRISLIDIGLVIEYLMGGAYRCNYTRKRFRTLYHNLFGPKRPKALKLLGMEDDIPLRRGRKTTKKREEEVDIDLDDPEINHFPFPFHELMVWAVLMKRQKMALFFWQHGEEAMAKALVACKLCKAMAHEASENDMVDDISQELNHNSRDFGQLAVELLDQSYKQDEQLAMKLLTYELKNWSNATCLQLAVAAKHRDFIAHTCSQMLLTDMWMGRLRMRKNSGLKVILGILLPPSILSLEFKNKDDMPYMTQAQEIHLQEKEPEEPEKPTKEKDEEDMELTAMLGRSNGESSRKKDEEEVQSRHRLIPVGRKIYEFYNAPIVKFWFYTLAYIGYLMLFNYIVLVKMERWPSTQEWIVISYIFTLGIEKMREILMSEPGKLLQKVKVWLQEYWNVTDLIAILLFSVGMILRLQDQPFRSDGRVIYCVNIIYWYIRLLDIFGVNKYLGPYVMMIGKMMIDMMYFVIIMLVVLMSFGVARQAILFPNEEPSWKLAKNIFYMPYWMIYGEVFADQIDPPCGQNETREDGKTIQLPPCKTGAWIVPAIMACYLLVANILLVNLLIAVFNNTFFEVKSISNQVWKFQRYQLIMTFHERPVLPPPLIIFSHMTMIFQHVCCRWRKHESDQDERDYGLKLFITDDELKKVHDFEEQCIEEYFREKDDRFNSSNDERIRVTSERVENMSMRLEEVNEREHSMKASLQTVDIRLAQLEDLIGRMATALERLTGLERAESNKIRSRTSSDCTDAAYIVRQSSFNSQEGNTFKLQESIDPAGEETISPTSPTLMPRMRSHSFYSVNVKDKGGIEKLESIFKERSLSLHRATSSHSVAKEPKAPAAPANTLAIVPDSRRPSSCIDIYVSAMDELHCDIEPLDNSMNILGLGEPSFSALAPSTTPSSSAYATLAPTDRPPSRSIDFEDLTSMDTRSFSSDYTHLPECQNPWDTDPPTYHTIERSKSSRYLATTPFLLEEAPIVKSHSFMFSPSRSYYANFGVPVKTAEYTSITDCIDTRCVNAPQAIADRATFPGGLGDKVEDLSCCHPEREAELSHPSSDSEENEARGQRAANPISSQEAENADRTLSNNITVPKIERANSYSAEEPNVPYAHTRKSFSISDKLDRQRNTASLRNPFQRSKSSKPEGRGDSLSMRRLSRTSAFHSFESKHN

> NP_001178491.1 -RnTrpm3

MGKKWRDAGEMERGCSDREGSAESRRRSRSASRGRFAESWKRLSSKQGSTKRSGLPAQQTPAQKSWIERAFYKRECVHIIPSTKDPHRCCCGRLIGQHVGLTPSISVLQNEKNESRLSRNDIQSEKWSISKHTQLSPTDAFGTIEFQGGGHSNKAMYVRVSFDTKPDLLLHLMTKEWQLELPKLLISVHGGLQNFELQPKLKQVFGKGLIKAAMTTGAWIFTGGVNTGVIRHVGDALKDHASKSRGKICTIGIAPWGIVENQEDLIGRDVVRPYQTMSNPMSKLTVLNSMHSHFILADNGTTGKYGAEVKLRRQLEKHISLQKINTRIGQGVPVVALIVEGGPNVISIVLEYLRDTPPVPVVVCDGSGRASDILAFGHKYSEEGGLINESLRDQLLVTIQKTFTYTRTQAQHLFIILMECMKKKELITVFRMGSEGHQDIDLAILTALLKGANASAPDQLSLALAWNRVDIARSQIFIYGQQWPVGSLEQAMLDALVLDRVDFVKLLIENGVSMHRFLTISRLEELYNTRHGPSNTLYHLVRDVKKGNLPPDYRISLIDIGLVIEYLMGGAYRCNYTRKRFRTLYHNLFGPKRPKALKLLGMEDDIPLRRGRKTTKKREEEVDIDLDDPEINHFPFPFHELMVWAVLMKRQKMALFFWQHGEEAMAKALVACKLCKAMAHEASENDMVDDISQELNHNSRDFGQLAVELLDQSYKQDEQLAMKLLTYELKNWSNATCLQLAVAAKHRDFIAHTCSQMLLTDMWMGRLRMRKNSGLKVILGILLPPSILSLEFKNKDDMPYMTQAQEIHLQEKEPEEPEKPTKEKDEEDMELTAMLGRNNGESSRKKDEEEVQSRHRLIPVGRKIYEFYNAPIVKFWFYTLAYIGYLMLFNYIVLVKMERWPSTQEWIVISYIFTLGIEKMREILMSEPGKLLQKVKVWLQEYWNVTDLIAILLFSVGMILRLQDQPFRSDGRVIYCVNIIYWYIRLLDIFGVNKYLGPYVMMIGKMMIDMMYFVIIMLVVLMSFGVARQAILFPNEEPSWKLAKNIFYMPYWMIYGEVFADQIDRKQVYDSHTPKSAPCGQNETREDGKTIQLPPCKTGAWIVPAIMACYLLVANILLVNLLIAVFNNTFFEVKSISNQVWKFQRYQLIMTFHERPVLPPPLIIFSHMTMIFQHVCCRWRKHESDPDERDYGLKLFITDDELKKVHDFEEQCIEEYFREKDDRFNSSNDERIRVTSERVENMSMRLEEVNEREHSMKASLQTVDIRLAQLEDLIGRMATALERLTGLERAESNKIRSRTSSDCTDAAYIVRQSSFNSQEGNTFKLQESIDPAGEETMSPTSPTLMPRMRSHSFYSVNVKDKGGIEKLESIFKERSLSLHRATSSHSVAKEPKAPAAPANTLAIVPDSRRPSSCIDIYVSAMDELHCDIDPLDNSMNILGLGEPSFSALAPSTAPSSSAYATLAPTDRPPSRSIDFEDLTSMDTRSFSSDYTHLPECQNPWDTDPPMYHTIERSKSSRYLATTPFLLEEAPIVKSHSFMFSPSRSYYANFGVPVKTAEYTSITDCIDTRCVNAPQAIADRATFPGGLGDKVEDLSCCHPEREAELSHPSSDSEENEARGRRAANPISSQETENADRTLSNNITVPKIERANSYSAEEPSAPYAHTRKSFSISDKLDRQRNTASLRNPFQRSKSSKPEGRGDSVSMRRLSRTSAFHSFESKHN

> XP_003582586.1 -BtTrpm3

MQVRKQQLELDMEQQTGSKIGKGITVFRMGSEGHQDIDLAILTALLKGANASAPDQLSLALAWNRVDIARSQIFIYGQQWPVGSLEQAMLDALVLDRVDFVKLLIENGVSMHRFLTISRLEELYNTRHGPSNTLYHLVRDVKKGNLPPDYRISLIDIGLVIEYLMGGAYRCNYTRKRFRTLYHNLFGPKRPKALKLLGMEDDVPLRRGRKTTKKREEEVDIDLDDPEINHFPFPFHELMVWAVLMKRQKMALFFWQHGEEAMAKALVACKLCKAMAHEASENDMVDDISQELNHNSRDFGQLAVELLDQSYKQDEQLAMKLLTYELKNWSNATCLQLAVAAKHRDFIAHTCSQMLLTDMWMGRLRMRKNSGLKVILGILLPPSILSLEFKNKDDMPYMTQAQEIHLQEKEQEEPEKPTKEKDEEDMELTAMLGRNNGESSRKKDEEEVQSRHRFIPLGRKIYEFYNAPIVKFWFYTLAYIGYLMLFNYIVLVKMERWPSTQEWIVISYIFTLGIEKMREILMSEPGKLLQKVKVWLQEYWNVTDLIAILLFSVGMILRLQDQPFRSDGRVIYCVNIIYWYIRLLDIFGVNKYLGPYVMMIGKMMIDMMYFVIIMLVVLMSFGVARQAILFPNEEPSWKLAKNIFYMPYWMIYGEVFADQIDRKQVYDSHTPKSAPCGQNETREDGKIIQLPPCKTGAWIVPAIMACYLLVANILLVNLLIAVFNNTFFEVKSISNQVWKFQRYQLIMTFHERPVLPPPLIIFSHMTMIFQHLCCRWRKHESDPDERDYGLKLFITDDELKKVHDFEEQCIEEYFREKDDRFNSSNDERIRVTSERVENMSMRLEEVNEREHCMKASLQTVDIRLAQLEDLIGRMATALERLTGLERAESNKIRSRTSSDCTDAAYIVRQSSFNSQEGNTFKLQESIDPAGEETLSPTSPTLMPRIRSHSFYSVNMKDKGGIEKLESLFKERSLSLHRATSSHSVAKESKAPAAPANTLAIVPDSRRPSSCIDIYVSAMDELHCDIDPLDNSMNILGLGEPSFSAPVPSAAPSSSAYATLAPTDRLPSRSTDFEDITSMDTRSFSSDYTHIPECQNPWDTDPPMYHTIERSKSSRYLATXEYTSITDCIDTRCVNAPQVIADRTTFLGGLGGKVEESLCCHPEREAELSHPSSDGEENEAKGRRATITMPPQEGDNSDRTLSNNITVPKIERANSYSAEEPSTPYAHTRKSFSISDKLDRQRNTASLRNPFQRSKSSKPEGRGDSLSMRRLSRMSAFHSFESKHN

> XP_424831.3 -ggTRPM3

MNQTDAPRPLNWTIRKLCHAAFLPSVRLLKAQKSWIERAFYKRECVHIVPSTKDPHRCCCGRLIGQHVGLTPSISIIQNEKNESRLTRNDIQSEKWSISKHTQLSPTDAFGTIEFQGGGHSNKAMYVRVSFDTKPDLLLHLMTKEWQLELPKLLISVHGGLQNFELQPKLKQVFGKGLIKAAMTTGAWIFTGGVNTGVIRHVGDALKDHASKSRGKICTIGIAPWGIVENQEDLIGKDVVRPYQTMSNPMSKLTVLNSMHSHFILADNGTTGKYGAEVKLRRQLEKHISLQKINTRIGQGVPVVALIVEGGPNVISIVLEYLRDTPPVPVVICDGSGRASDILAFGHKYSEEGGLINESLRDQLLVTIQKTFTYTRTQAQHLFIILMECMKKKELITVFRMGSEGHQDIDLAILTALLKGANASAPDQLSLALAWNRVDIARSQIFIYGQQWPVGSLEQAMLDALVLDRVDFVKLLIENGVSMHRFLTISRLEELYNTRHGPSNTLYHLVRDVKKGNLPPDYRISLIDIGLVIEYLMGGAYRCNYTRKRFRTLYHNLFGPKRPKALKLLGMEDDVPLRRGRKTTKKREEEVDIDLDDPEINHFPFPFHELMVWAVLMKRQKMALFFWQHGEEAMAKALVACKLCKAMAHEASENDMVDDISQELNHNSRDFGQLAVELLDQSYKQDEQLAMKLLTYELKNWSNATCLQLAVAAKHRDFIAHTCSQMLLTDMWMGRLRMRKNSGLKVILGILLPPSILSLEFKNKDDMPYMSQANEIHLQEKEPEEPEKPVKEKEEEDMELTAMLGRGNGESSRKKEEEEVQSRHRLIPVGRKIYEFYNAPIVKFWFYTLAYIGYLMLFNYIVLVKMDRWPSTQEWIVISYIFTLGIEKMREILMSEPGKLLQKVKVWLQEYWNVTDLIAILLFSVGMVLRLQDLPLRSDGRVIYCVNIIYWYIRLLDIFGVNKYLGPYVMMIGKMMIDMMYFVIIMLVVLMSFGVARQAILFPNEEPSWKLAKNIFYMPYWMIYGEVFADQIDPPCGQNETREDGKIIQLPPCKTGAWIVPAIMACYLLVANILLVNLLIAVFNNTFFEVKSISNQVWKFQRYQLIMTFHERPVLPPPLIIFSHMTMIFQHLCCRWRKHESDPDERDYGLKLFITEDELKKVHDFEEQCIEEYFREKDDRFNSSNDERIRVTSERVENMAMRLEEVNEREHCMKASLQTVDIRLAQLEDMIGRMVTALEKLTGIERGEATKIRSRTSSDCTDAAYIVRQSSFNSQEGNTYKLQESIDPTGEESMSPTSPTITPRMRSHSFYATNMKDKCGLEKFESIFKERSPSLHRASSSHSLAKEGKVPLAPTSTLSIAPDSRRPSSCIDIYVSAMDEMHSDTEPLDSSINILGTGEVALQAHIASSILANSAYISSTPGEKISGRSLDYEETSGIETRAFSSDYSQITECQIPWDSDPPLYHTLERSKSSRYLATTPFILEETPIVKSHSFMFSPSRSYFSSLGVPVKTAEYTSITDCIDTRCVSAPQTIAERASFPGSLGVKVEDLGCCHPEREAELSHPSSDHEDTEAKEKKGIPLSPQDSNAARTLTNSIPLPKIERANSYSAEESNMLYAQHTRKSYSISDKLDRQRNATSLRNPFQRSKSSRPESRGDNLSMRRLSRTGAFRSFESKHS

> XP_002935641.1 -XtTrpm3

MAQKSWIERAFHKRECVHIIPSTKDPHRCCCGRLIGQHVGLTPSISIIQNEKNEGRHRNDIQSEKWSIGKHTQLSPTDAFGTIEFQGGGHSNKAMYLYLGYNVQDCIYILVHVAMYLQLSRIQPSPHLRLRNGQLSPRLSQVVAFCKNYIKQKEKNKKWQFTLKRKKGVIRHVGDSLKDHASKSRGKICTIGIAPWGIVENQEDLVGKDVVRPYQTMSNPMSKLTVLNSMHSHFILADNGTTGKYGAEVKLRRQLEKHISLQKINTICQSQTSLLSILSVSYWGFRSSSSIGIGQGVPVVALIVEGGPNVISIVLEYLRDNPPVPVVVCDGSGRASDILAFGHKYSEEGGLINESLRDQLLVTIQKTFTYTRTQAQHLFIILMECMKKKELITVFRMGSEGHQDIDLAILTALLKDYTGANASAPDQLSLALAWNRVDIARSQIFIYGQQWPVGSLEQAMLDALVLDRVDFVKLLIENGVSMHRFLTISRLEELYNTRHGPSNTLYHLVRDVKKGNLPPDYRISLIDIGLVIEYLMGGAYRCNYTRKRFRTLYHNLFGPKRPKALKLLGMEDDIPIRSGRKTTKKREEEIDIDLDDPEINHFPFPFHELMVWAVLMKRQKMALFFWQHGEEAMAKALVACKLCKAMAHEASENDMVDDISQELNHNSREFGQLAVELLDQSYKQDEQLAMKLLTYELKNWSNATCLQLAVAAKHRDFIAHTCSQMLLTDMWMGRLRMRKNSSLKVILGILLPPSILSLEFKNKDEMPYMSQANEMDLQEKEVEEPDKTVKEKEEDDMELTAMLARGNGDSSRKKDEDEHPRHRLIPIGRKIYEFYNAPIVKFWFFTMAYMGYLMLFNYIVLVKMDRWPSMQEWIVISYIFTLGIEKMREILMSEPGKLLQKFKVWLQEYWNLADLVSILLFSIGMVLRLQEQPFSSYGRVIYCVNIIYWYIRLLDIFGVNKYLGPYVMMIGKMMIDMMYFVIIMLVVLMSFGVARQAILFPSEEPSWNLAKNIFYMPYWMIYGEVFADQIDPPCGYNVTGEDGKIIQLPPCKTGAWIVPAIMACYLLVANILLVNLLIAVFNNTFFEVKSISNQVWKFQRYQLIMTFHERPVLPPPLIIFSHFTMICKHLCCRWRKRELDQDERDYGLKLFITEDDLKKVHDFEEQCIEEYFREKDDRFNSSNDERIRVTAERVENMSMRLEEVNEREHFMKASLQTVDIRLAQLEEMLGRMATAMERLTGVERGETNKARSRTSSDCTDAAYIVRQSSFNSQEGNTYKLHDIIDPVGEEPLSPTSPNLTPRMRSQSFYSSNLRDKGGIERFEGFFKERTLNLHRAISSHSISKDAKPPSSPLNSLSVVPDSRRPSSCIDIYVSAMDEAQFCLDPGESSMNVACTGDPMGDIGSIGQGCSSSTHCGRSCSRNPSYEESIPMDPFTFPQEITGIPISQNPWDTESSLCHTLERSKSSRFLATAPFILEETPIVKSQTFMFSPCRSYYNNLGVPVKTAEYTSITDCIDTRCVSNTPQATAERSTSPGCVREKVEDVSSCHPEREAELSHPSSDNEDNDEEVRNKCTLTSLSAHESSSSRTLYNNIPFPKIERANSYSAEEPSMTYSHTKKSYSISDKLDRQRTTASLRNPFQRSKSSRPESRGDNLSMRRLSRTAAFRSFESKHS

> XP_694167.4 -DrTrpm3

MAQKSWIERAFSKRECVHIIVSTKDHHRCCCGRLIGQHVGLPPSISSNQNEKSERVPKNDSLSEKWSISKHTQLSPTDAFGTIEFQGGGHSNKAMYVRVSYDTKPDLLLHLMTKEWQLELPKLLISVHGGLQNFELQPKLKQVFGKGLIKAAMTTGAWIFTGGVNTGVIRHVGDALKDHASKSRGKICTIGIAPWGIVENQEDLVGKDVVRPYQTMSNPLSKLTVLNSLHSHFILADNGTTGKYGAEVKLRRQLEKHISLQKINTRIGQGVPVVALIVEGGPNVISIVLEYLRDTPPVPVVVCDGSGRASDILAFGHKYSEEGGIINESLRDQLLVTIQKTFTYSRTQAQHLFIILMECMKKKELITVFRMGSEGHQDIDLAILTALLKGANASAPDQLSLALAWNRVDIARSQIFIYGQQWPVGSLEQSMLDALVLDRVDFVKLLIENGVSMHRFLTLSRLEELYNTRHGPSNTLYHLVRDVKKGNLPPDYRISLIDIGLVIEYLMGGAYRCNYTRKRFRTLYHNLFGPKRPKALKLLGMEDDMPIRRGRQKTTRKREEEVDIDLDDPEINHFPFPFHELMVWAVLMKRQKMALFFWQHGEEAMAKALVACKLCKAMAHEASENDMVDDISQELNQNSREFGQLAVELLDQSYKQDEQMAMKLLTYELKNWSNATCLQLAVAAKHRDFIAHTCSQMLLTDMWMGRLRMRKNSGLKVILGLLLPPSILSLEFKNKDEMSYMPQDQDTYLQEKDVDEPEKQAKEKEEEDMEFTVRSYCETQYNSVAMLGNVSSEASRKKQVEEVQNRHRLIPLGRKIYEFYNAPIVKFWFHTKVHDCHRLIPVGRKIKVQDRNLLILVGHKMYEFYNNPFIKFWFCMVNSIIYYTKTNCTLAYVGYLMLFNYIVLVKMDLWPSPQEWIVIAYIFTNGIEKMREILMSEPGKLLQKVKVWLQEYWNITDLMAILIFSIGMVLRLQDPPLMSYGRVIYCVNIIYWYIRLLDIFGVNKYLGPYVMMIGKMMIDMMYFVIIMLVVLMSFGVARQAILNPNEDPSWMLARNIFFMPYWMIYGEVFADQIDPPCGQNITTEEGVVMPLPPCKTGAWIVPAIMACYLLVANILLVNLLIAVFNNTFFEVKSISNQVWKFQRYQLIMTFHERPVLPPPLIIFSHITMVLKHLCCRWRKHDEDERDYGLKLFITEDELKKVHDFEEQCMEEYFREKDDRFNSSNDERIRVTSERVENMAMRLEEVNEREHFMKASLQTVDIRLAQMEEMIGRIAVALERVAGMDRGEVNKARSRTSSDCTDTNYILRQSSFNSQEGNSYRLQESLEQGGDESISPTSPTALAPRVRSHSFYVSHSSKDRSGADRGEGFFKDKLFSLHRANSSQSVSSGAGPKESKPTPLNTLSVQQQLRPSSCIDIYVSASEDVPPTESFLDSVRTVPTLARDSSLHSEIMEAVLSGGRDCSGRAGGSERQSDGTVLFEDSAAADLSLCSAHLLPDSLPPWDLDPSPPPSAGVLERSKSSRFLSTAGPLFLDEPHLVKSHSLMFTSRGYYGGMGVQVKAAEYTSITDCIDTRCVSTPYPVPERSDSPGGSFTFDKPQDLGVSHPERDAELSHAESDLEEPAEGSGDTCKVGQSSSSGVIGADLGLGLALGPFCSPISRLERANSCSSSEESHSNIYSRKSFSISERMDKGRGSSRNPFQKARTGARLEGKTDSLSMRKMAKPSAFRSFDSRHNYT

> XP_003452698.1 -olTrpm3

MYVRVSYDTKPDLLLHLMTKEWQLDLPKLLISVHGGLQNFELQPKLKQVFGKGLIKAAMTTGAWIFTGGVNTGVIRHVGDALKDHASKSRGKICTIGIAPWGIVENQEDLVGKDVVRPYQTMSNPLSKLTVLNSLHSHFILADNGTTGKYGAEVRLRRQLEKHISLQKINTRCIHFFHRYSNMYYFMLNTCQSQRTGIGQGVPVVALIVEGGPNVISIVLEYLRDTPPVPVVVCDGSGRASDILAFGHKYSEEGGIINESLRDQLLVTIQKTFTYSRTQAQHLFIILMECMKKKELITVFRMGSEGHQDIDLAILTALLKGANASAPDQLSLALAWNRVDIARSQIFIYGQQWPVGSLEQAMLDALVLDRVDFVKLLIENGVSVHRFLTLSRLEELYNTRHGPSNTLYHLVRDVKKGNLPPDYRISLIDIGLVIEYLMGGAYRCNYTRKRFRTLYHNLFGPKRPKALKLLGMEDDMPIRRGRQKTTRKREEEVDIDLDDPEINHFPFPFHELMVWAVLMKRQKMALFFWQHGEEAMAKALVACKLCKAMAHEASENDMVDDISQELNHNSREFAQLAVELLDQSYKQDEQMAMKLLTYELKNWSNATCLQLAVAAKHRDFIAHTCSQMLLTDMWMGRLRMRKNSGLKVILGLLLPPSILSLEFKNKDEMSYMPQDQEAYLKSKGKEKKKSSNKYGVKKISDLIAMLGKVATDTSRKKDVEEVQSRHRLIPLGRKIYEFYNAPIVKFWFHTLAYVGYLMLFNYIVLVKMDLWPSPQEWIVIAYIFTNGIEKMREILMSEPGKLLQKVKVWLQEYWNITDLMAILIFSVGMVLRLQEPPFMSYGRVIYCINIIYWYIRLLDIFGVNKYLGPYVMMIGKMMIDMMYFVIIMLVVLMSFGVARQAILNPNEDPSWMLARNIFFMPYWMIYGEVFADQIDPPCGQNITTDDGVVVALPACKTGAWIVPAIMACYLLVANILLVNLLIAVFNNTFFEVKSISNQVWKFQRYQLIMTFHERPVLPPPLIIFSHITMVLKHLCCRWRKHDDDERDYGLKLFITEDELKKVHDFEEQCIEEYFREKDDRFHSSNDERIRVTSESIRVENMAMRLEEVNEREHFMKASLQTVDIRLAQMEELIGRIAVALERVTGVERVEVGKARSRTSSDCTDSAYILRQAECQDTAYILRQSSFNSTEGNTYRLQEALEGTAEGSMSPPSPTTTGTRARSHSFYVGGGRGAERVRGPEGAESFFKERSLSLHRANSSQSVSSAAAPKESKPLPLATLSVSQQHRPSSCIDIYVSTSEEVGPAEVFLDSLRVIPPLQREASLQSDIMETVLPEGRGFGSTATSGLGDRHSEGGVGSSGTTGAMFDDSAAADLSLCSAHLLPDTTLPPWDMEPSPPPSAGLLERSKSSRYLSTSGTAFLDEPPLVKSHSLMFTPRGCYGGLGAGVQVKATEYTSITDCIDTRCVSAPYTPAECSHSPGGSTSFPFDKPSDISSSYPEREAELSHTESDPEDPEDMIPASDTHRHSGLGGPSTAPLCSHFSRLERANSCSSDDSHPSLTRAPPHREKLSVSERMERGRGLAGPRNPFLRSKSGARPEKTESLAIRKLAAPSAFRSFDRQNY

* The phylogenetic tree in Phylip format

((BtTrpm1:0.232188,(((ggTrpm1:0.001000,XtTrpm1:0.246488):0.027724,(((((ggTRPM3:0.001000,(RnTrpm3:0.001000,mmTRPM3:0.216762):0.027242):0.030636,(BtTrpm3:0.076757,hTRPM3:0.124201):0.047542):0.008072,XtTrpm3:0.259791):0.039904,(olTrpm3:0.057260,DrTrpm3:0.229584):0.020138):0.211074,((DrTrpm1a2:0.001000,olTRPM1:0.293172):0.116804,DrTrpm1b7:0.219232):0.026877):0.072475):0.034905,(mmTrpm1:0.001000,RnTrpm1:0.226402):0.067729):0.017480):0.108188,hTrpm1:0.108188);

Distance Matrix 1 2 3 4 5 6 7 8 9 10 11 12 13 14 15 16 17

1 hTrpm1 0.000 0.147 0.149 0.137 0.197 0.256 0.424 0.384 0.467 0.578 0.535 0.532 0.746 0.521 0.562 0.560 0.591

2 mmTrpm1 0.147 0.000 0.037 0.193 0.219 0.280 0.415 0.394 0.460 0.576 0.528 0.523 0.746 0.520 0.558 0.561 0.594

3 RnTrpm1 0.149 0.037 0.000 0.200 0.221 0.284 0.419 0.400 0.464 0.580 0.531 0.523 0.745 0.521 0.559 0.562 0.596

4 BtTrpm1 0.137 0.193 0.200 0.000 0.211 0.245 0.439 0.397 0.481 0.584 0.524 0.521 0.752 0.519 0.569 0.572 0.602

5 ggTrpm1 0.197 0.219 0.221 0.211 0.000 0.181 0.426 0.375 0.470 0.569 0.515 0.512 0.738 0.514 0.555 0.550 0.579

6 XtTrpm1 0.256 0.280 0.284 0.245 0.181 0.000 0.447 0.405 0.488 0.585 0.519 0.510 0.750 0.522 0.568 0.567 0.595

7 DrTrpm1a2 0.424 0.415 0.419 0.439 0.426 0.447 0.000 0.424 0.231 0.633 0.604 0.600 0.784 0.587 0.613 0.618 0.642

8 DrTrpm1b7 0.384 0.394 0.400 0.397 0.375 0.405 0.424 0.000 0.384 0.530 0.568 0.565 0.709 0.554 0.583 0.582 0.548

9 olTRPM1 0.467 0.460 0.464 0.481 0.470 0.488 0.231 0.384 0.000 0.600 0.650 0.647 0.759 0.636 0.662 0.660 0.607

10 hTRPM3 0.578 0.576 0.580 0.584 0.569 0.585 0.633 0.530 0.600 0.000 0.163 0.161 0.295 0.185 0.225 0.318 0.244

11 mmTRPM3 0.535 0.528 0.531 0.524 0.515 0.519 0.604 0.568 0.650 0.163 0.000 0.058 0.385 0.099 0.210 0.280 0.358

12 RnTrpm3 0.532 0.523 0.523 0.521 0.512 0.510 0.600 0.565 0.647 0.161 0.058 0.000 0.366 0.116 0.212 0.282 0.360

13 BtTrpm3 0.746 0.746 0.745 0.752 0.738 0.750 0.784 0.709 0.759 0.295 0.385 0.366 0.000 0.406 0.440 0.524 0.489

14 ggTRPM3 0.521 0.520 0.521 0.519 0.514 0.522 0.587 0.554 0.636 0.185 0.099 0.116 0.406 0.000 0.188 0.259 0.334

15 XtTrpm3 0.562 0.558 0.559 0.569 0.555 0.568 0.613 0.583 0.662 0.225 0.210 0.212 0.440 0.188 0.000 0.297 0.354

16 DrTrpm3 0.560 0.561 0.562 0.572 0.550 0.567 0.618 0.582 0.660 0.318 0.280 0.282 0.524 0.259 0.297 0.000 0.297

17 olTrpm3 0.591 0.594 0.596 0.602 0.579 0.595 0.642 0.548 0.607 0.244 0.358 0.360 0.489 0.334 0.354 0.297 0.000
